# Single-dose ^177^Lu-PSMA-617 followed by maintenance pembrolizumab in patients with metastatic castration-resistant prostate cancer: an open-label, dose-expansion, phase 1 trial

**DOI:** 10.1016/S1470-2045(23)00451-5

**Published:** 2023-11

**Authors:** Rahul Aggarwal, Stephanie Starzinski, Ivan de Kouchkovsky, Vadim Koshkin, Rohit Bose, Jonathan Chou, Arpita Desai, Daniel Kwon, Samuel Kaushal, Lauren Trihy, Medini Rastogi, Robin Ippisch, Maya Aslam, Terence Friedlander, Felix Feng, David Oh, Alexander Cheung, Eric Small, Michael Evans, Lawrence Fong, Thomas A Hope

**Affiliations:** Helen Diller Family Comprehensive Cancer Center, University of California San Francisco, San Francisco, CA, USA; Helen Diller Family Comprehensive Cancer Center, University of California San Francisco, San Francisco, CA, USA; Helen Diller Family Comprehensive Cancer Center, University of California San Francisco, San Francisco, CA, USA; Helen Diller Family Comprehensive Cancer Center, University of California San Francisco, San Francisco, CA, USA; Helen Diller Family Comprehensive Cancer Center, University of California San Francisco, San Francisco, CA, USA; Helen Diller Family Comprehensive Cancer Center, University of California San Francisco, San Francisco, CA, USA; Helen Diller Family Comprehensive Cancer Center, University of California San Francisco, San Francisco, CA, USA; Helen Diller Family Comprehensive Cancer Center, University of California San Francisco, San Francisco, CA, USA; Helen Diller Family Comprehensive Cancer Center, University of California San Francisco, San Francisco, CA, USA; Helen Diller Family Comprehensive Cancer Center, University of California San Francisco, San Francisco, CA, USA; Helen Diller Family Comprehensive Cancer Center, University of California San Francisco, San Francisco, CA, USA; Department of Radiology and Biomedical Imaging, University of California San Francisco, San Francisco, CA, USA; Department of Radiology and Biomedical Imaging, University of California San Francisco, San Francisco, CA, USA; Helen Diller Family Comprehensive Cancer Center, University of California San Francisco, San Francisco, CA, USA; Helen Diller Family Comprehensive Cancer Center, University of California San Francisco, San Francisco, CA, USA; Helen Diller Family Comprehensive Cancer Center, University of California San Francisco, San Francisco, CA, USA; Helen Diller Family Comprehensive Cancer Center, University of California San Francisco, San Francisco, CA, USA; Helen Diller Family Comprehensive Cancer Center, University of California San Francisco, San Francisco, CA, USA; Department of Radiology and Biomedical Imaging, University of California San Francisco, San Francisco, CA, USA; Helen Diller Family Comprehensive Cancer Center, University of California San Francisco, San Francisco, CA, USA; Department of Radiology and Biomedical Imaging, University of California San Francisco, San Francisco, CA, USA

## Abstract

**Background:**

Checkpoint inhibitors have been shown to have limited activity in patients with metastatic castration-resistant prostate cancer. We aimed to determine whether a single dose of lutetium-177 [^177^Lu]-prostate-specific membrane antigen (PSMA)-617 (^77^Lu-PSMA-617) followed by maintenance pembrolizumab was safe and could induce durable clinical benefit.

**Methods:**

We did an open-label, dose-expansion, phase 1 study at the University of California, San Francisco (San Fransisco, CA, USA). Eligible patients were men aged 18 years or older with progressive metastatic castration-resistant prostate cancer who had an Eastern Cooperative Oncology Group performance status of 0 or 1, had progression on one or more androgen signalling inhibitors, and at least three PSMA-avid lesions on ^68^Ga-PSMA-11 positron emission tomography. In part A, patients were enrolled sequentially to one of three schedules in which a single dose of ^177^Lu-PSMA-617 (7·4 GBq) was given intravenously 28 days before (schedule 1), concomitant with (schedule 2), or 21 days after (schedule 3) the start of maintenance intravenous pembrolizumab (200 mg every 3 weeks). In part B, 25 patients were enrolled using the recommended phase 2 schedule. The primary endpoint in part A was determination of the recommended phase 2 schedule, and in part B, the objective response rate. The analysis set included all patients who received at least one dose of pembrolizumab or ^177^Lu-PSMA-617. This study is registered with ClinicalTrials.gov, NCT03805594.

**Findings:**

Between Aug 8, 2019 and May 7, 2022, 43 male patients were enrolled (n=18 part A [six patients per schedule]; n=25 part B), with a median follow-up of 16·5 months (IQR 12·2–21·9). Schedule 1 was selected as the recommended phase 2 schedule for part B, on the basis of safety and feasibility of administration observed in part A. In part B, 14 (56%; 95% CI 35–76) of 25 patients had a confirmed objective response. Two (5%) of 43 patients had a treatment-related adverse event of grade 3 or worse (grade 3 arthritis in schedule 2, grade 3 pneumonitis in schedule 3). One serious adverse event (one death due to aspiration pneumonia) and no treatment-related deaths were observed.

**Interpretation:**

A single priming dose of ^177^Lu-PSMA-617 followed by pembrolizumab maintenance was safe and had encouraging preliminary activity in patients with metastatic castration-resistant prostate cancer.

**Funding:**

Prostate Cancer Foundation, National Cancer Institute, Novartis Pharmaceuticals, and Merck.

## Introduction

Immune checkpoint inhibition has poor efficacy in prostate cancer, in part due to an immunosuppressive microenvironment and low tumour mutational burden relative to other malignancies.^1-5^ Attempts to improve outcomes with combination regimens including androgen signalling or poly(ADP-ribose) polymerase PARP inhibition have been largely unsuccessful with regard to additive or synergistic activity.^6,7^

Radiotherapy has the potential to enhance anti-tumour effects of immune checkpoint blockade via multiple mechanisms, including stimulating antigen presentation, releasing proinflammatory signals from dying tumour cells, and enhancing the diversity of the intratumoral T-cell receptor repertoire.^8-15^ A phase 3 study in patients with metastatic castration-resistant prostate cancer given a single dose of external beam radiation followed by ipilimumab found that a subset of patients had durable disease control and long-term survival with extended follow-up.^16,17^

Lutetium-177 [^177^Lu]-prostate-specific membrane antigen (PSMA)-617 (^177^Lu-PSMA-617) is a β-particle-emitting radiopharmaceutical that has been found to prolong survival in patients with metastatic castration-resistant prostate cancer who have received previous taxane chemotherapy and one or more androgen signalling inhibitors.^18^ Targeted radioligand therapy might enhance the effectiveness of immune checkpoint inhibition by enhancing priming of an immune response or resetting the immunosuppressive tumour microenvironment to enhance effector function.^19^ We postulated that if ^177^Lu-PSMA-617 induced anti-tumour immunity, the immune effectors recruited to the sites of metastasis might be ablated with repeated doses of radioligand therapy administered on a fixed schedule. We therefore aimed to investigate the activity of a single priming dose of targeted radioligand therapy combined with maintenance immune checkpoint inhibition in patients with metastatic castration-resistant prostate cancer.

## Methods

### Study design and participants

We did an open-label, dose-expansion, phase 1 study of a single priming dose of ^177^Lu-PSMA-617 in combination with maintenance pembrolizumab in patients with metastatic castration-resistant prostate cancer at the University of California, San Francisco (San Francisco, CA, USA). In part A, three schedules of priming ^177^Lu-PSMA-617 were assessed. Dose expansion in phase 1b was subsequently evaluated using the recommended phase 2 schedule.

Eligible patients were men aged 18 years or older, with an Eastern Cooperative Oncology Group performance status of 0 or 1, who had histologically confirmed metastatic castration-resistant prostate cancer with progression at study entry, as per the Prostate Cancer Working Group 3 (PCWG3) criteria.^20^ Patients were required to have progression on one or more second-generation androgen signalling inhibitor (abiraterone, enzalutamide, darolutamide, or apalutamide) before study entry, measurable disease by Response Evaluation Criteria in Solid Tumours (RECIST) 1.1 criteria,^21^ and three or more positron emission tomography (PET)-avid lesions on ^68^Ga-PSMA-11 PET screening (PET-avid lesions initially defined as those with uptake higher than mediastinal blood pool; the protocol was subsequently amended after the enrolment of the first 30 patients to require that positive lesions had higher uptake on PSMA PET than that of the liver, as per established criteria.^22^ Patients could have received previous taxane chemotherapy for the treatment of castration-sensitive disease, but not for metastatic castration-resistant prostate cancer. No molecular selection criteria were applied. For full list of inclusion and exclusion criteria, please see the study protocol (appendix).

The study protocol was approved by the ethics committees at the University of California San Francisco and regulatory authorities. All patients provided written informed consent, and the study adhered to the Declaration of Helsinki and Good Clinical Practice guidelines.

### Procedures

In part A of the study, patients were enrolled sequentially to one of three schedules in which a single priming dose of ^177^Lu-PSMA-617 (7·4 GBq [200 mCi]) was given intravenously 28 days before (schedule 1), concomitant with (schedule 2), or 21 days after (schedule 3) the start of maintenance intravenous pembrolizumab (200 mg every 3 weeks). Dose-limiting toxicities were defined in treatment cycles 1 and 2 (ie, the first two 3-week treatment cycle with pembrolizumab) as non-haematological treatment-related adverse events of grade 3 or worse, grade 4 thrombocytopenia (or grade 3 thrombocytopenia with clinically significant bleeding), grade 4 neutropenia lasting for more than 5 consecutive days, or grade 3 or worse febrile neutropenia. In the dose-expansion part B of the study, patients were enrolled using the recommended phase 2 schedule. All patients continued maintenance pembrolizumab until radiographical progression as per PCWG3 criteria, unequivocal clinical progression, or unacceptable toxicity.

No dose reductions of ^177^Lu-PSMA-617 were permitted. Dose interruptions, but not reductions, of pembrolizumab were permitted for adverse events of grade 3 or worse or intolerable adverse events of grade 2 or worse. Repeat dosing of ^177^Lu-PSMA-617 was not permitted while patients were receiving pembrolizumab, but was allowed per treating provider discretion after disease progression on pembrolizumab. Treatment interruption in the absence of progression on pembrolizumab was permitted after 35 cycles of treatment or a complete response for at least 6 months.

Gender and ethnicity data were obtained by patient self-report. Clinical and laboratory assessments in phase 1b of the study were done on days 1, 8, and 15 of cycle 1, and on day 1 of every cycle thereafter. Clinical assessment included assessment of adverse events, graded using Common Terminology Criteria for Adverse Events (CTCAE; version 4.03), assessment of vital signs, and a focused physical exam. Laboratory assessments included complete blood count with differential, electrolytes, liver, and thyroid function tests.

Tumour response and progression monitoring was performed using whole-body technetium-99m bone scintography and cross-sectional imaging (CT or MRI) of the chest, abdomen, and pelvis at baseline and every 9 weeks until confirmed progression by RECIST (version 1.1) and PCWG3 criteria. Percentage change in tumour size from baseline was calculated as the sum of longest diameter of target lesions as per RECIST 1.1 criteria. Scans were assessed by investigators and were not centrally reviewed. Patients who discontinued study treatment for reasons other than progression continued to have restaging scans performed per protocol. A repeat ^68^Ga-PSMA-11 PET scan was optional at the time of disease progression. After progression, all patients entered long-term follow-up until death or withdrawal from study.

Somatic and germline mutation status was ascertained for all patients using a Clinical Laboratory Improvement Amendments-approved assays (Invitae Common Hereditary Cancers Panel [Invitae Laboratories, San Francisco, CA, USA]; FoundationOne CDx and FoundationOneLiquid CDx [Foundation Medicine, Cambridge, MA, USA]; UCSF500 [University of California, San Francisco]).

Whole blood was collected at baseline before the initiation of study treatment (cycle 1, day 1) and serially on study treatment (cycle 2, day 1; cycle 3, day 1; cycle 6, day 1; cycle 9, day 1). Changes in the blood immune response was assessed using mass cytometry by time-of-flight (CyTOF). Processing instructions for CyTOF are described in the appendix (pp 1-3-7). Metastatic tumour biopsies were obtained before and after the priming dose of ^177^Lu-PSMA-617 to assess infiltrating immune cell subsets.

### Outcomes

The primary endpoint in part A was to determine the recommended phase 2 dose schedule of the treatment combination, based on the occurrence of dose-limiting toxicities, aggregate safety data, and feasibility of administration. The primary endpoint in part B was objective response rate (ORR) per investigator assessment by RECIST (version 1.1) criteria using cross-sectional imaging of the chest, abdomen and pelvis obtained at 9-week intervals, confirmed by repeat measurement. Secondary endpoints evaluated in the overall cohort were safety and dose-limiting toxicities (as defined above), median duration of response, the proportion of patients who had a decrease in prostate-specific antigen (PSA) of 50% or more (PSA50) from baseline, median PSA progression-free survival, median radiographic progression-free survival, 6-month radiographic progression-free survival, median time to symptomatic skeletal related event (SSRE), and median overall survival. Progression-free survival was defined as the time from the start of protocol therapy to radiographical progression as per PCWG3 criteria, clinical progression, or death, whichever occurred first; PSA progression was defined as time to date of first PSA measurement meeting criteria for progression by PCWG3; time to SSRE was defined as the time to first occurrence of symptomatic fracture, surgery or radiation to the bone, or spinal cord compression. Duration of response was defined as time from date of first objective response on imaging until the date of progression. Overall survival was defined as time from date of first treatment on study to death from any cause.

Prespecified exploratory endpoints were lesion-specific response by uptake on screening ^68^Ga-PSMA-11 PET, percentage change from baseline in T-cell repertoire, including circulating T-cell subsets and tumour infiltrating lymphocytes, tumour programmed death-ligand 1 (PD-L1) expression, descriptive characterisation of patterns of uptake on PSMA PET at the time of progression, the association between alterations in genes contained within the homologous recombination repair pathway (*BRCA1, BRCA2, ATM, PALB2, BRIP1, CDK12, CHEK2, FANCL, RAD51B, RAD51C, RAD51D*, and *RAD54L*), tumour mutational burden, and microsatellite status with clinical outcomes including response rate and progression free survival, and the association between tumour dosimetry with objective response. Analyses pertaining to changes in infiltrating immune cell subsets and PD-L1 expression by immunohistochemistry in paired metastatic biopsies, and the analysis of the association between tumour dosimetry and response, are ongoing and will be published elsewhere.

### Statistical analysis

In part A, six patients were enrolled per schedule to obtain sufficient safety data to determine the recommended phase 2 schedule, without an a-priori assumption of the optimal schedule. In part B, using a historical control objective response rate with pembrolizumab monotherapy in metastatic castration-resistant prostate cancer of 10%, and an alternative hypothesis whereby objective response rate would be 40%, we calculated that a sample size of 25 patients would provide 93% power to detect a difference of this magnitude in response rate with a two-sided level of significance of 0·05.

All patients who received at least one dose of protocol therapy (ie, one dose of pembrolizumab or ^177^Lu-PSMA-617) were included in the analysis of primary, secondary, and safety endpoints. Summary statistics were used to describe baseline patient and treatment characteristics. The recommended phase 2 schedule was determined based on the occurrence of dose-limiting toxicities and aggregate safety and feasibility data. Objective response was defined as the best response by RECIST criteria from the start of treatment until disease progression, confirmed by repeat measurement at least 4 weeks later. The proportion of patients in part B with confirmed objective response was reported descriptively with 95% CIs. The median duration of response, median progression-free survival, 6-month progression-free survival rate, median PSA progression-free survival, median time to SSRE, and median overall survival were estimated using the Kaplan-Meier method. Patients who withdrew from study participation for reasons unrelated to study participation before an event were censored for the time-to-event analyses; censoring was non-informative. Adverse events were tabulated by schedule using CTCAE (version 4.03).

A prespecified interim safety analysis was performed in part B after the enrolment of six patients; study accrual proceeded since the dose-limiting toxicity rate of 33% was not exceeded.

The association between maximal percentage change in measurable soft tissue lesions and uptake on baseline PSMA PET was performed by fitting a linear mixed model with repeated measures (appendix p 2). Patterns of uptake on PSMA PET at baseline and at progression were graphically represented. The t-value (degrees of freedom [df]) were computed for each coefficient. A p value of less than 0·05 was considered to indicate statistical significance.

We did post-hoc analyses to determine PSA90 response rate in the overall cohort and in part B, the PSA50 response rate; ORR in patients with visceral metastases and with soft tissue lesions with low or no expression of PSMA; PSA50 response rate on post-progression treatment with ^177^Lu-PSMA-617; objective response, PSA50 response, and progression-free survival by treatment schedule.

All statistical analyses were done using R (version 4.3.1). The study is registered with ClinicalTrials.gov, NCT03805594.

### Role of the funding source

The funders had no role in study design, data collection, data analysis, data interpretation, or writing of the report.

## Results

**Figure 1:**
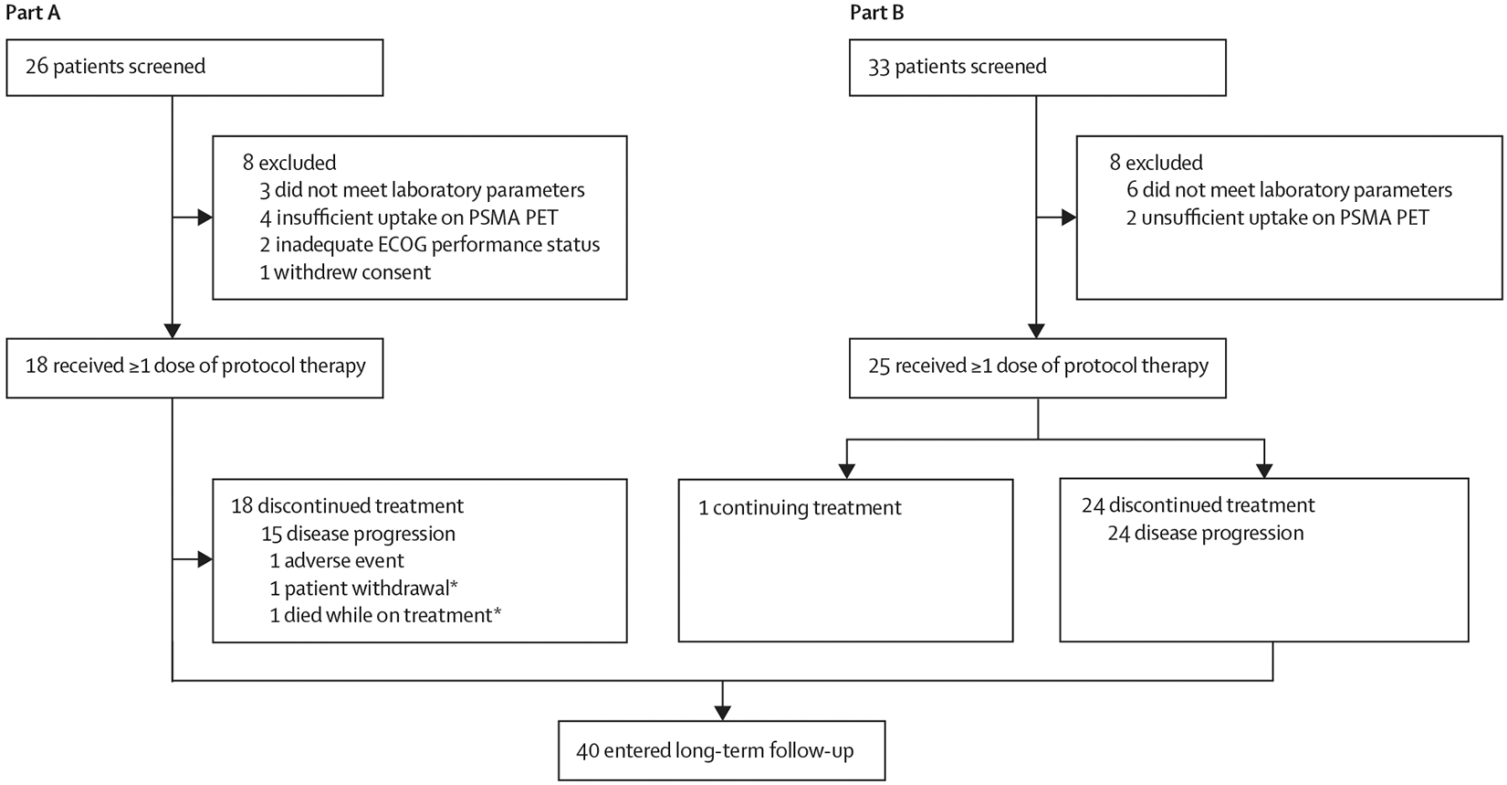
Trial profile PSMA=prostate-specific membrane antigen. PET=positron emission tomography. ECOG=Eastern Cooperative Oncology Group. *One patient who withdrew from the study and one patient who died on treatment did not enter long-term follow-up.

Between Aug 8, 2019, and May 7, 2022, 59 male patients were screened, of whom 43 were enrolled (18 patients in part A; 25 patients in part B) and received one dose of protocol therapy (figure 1). The median follow-up was 16·5 months (IQR 12·2–21·9). Baseline characteristics are shown in table 1. The median duration of treatment in the overall study cohort was 4·8 months (range 1·1–21·0; appendix p 8).

In part A of the study, no dose-limiting toxicities were observed. Schedule 1 (^177^Lu-PSMA-617 followed by initiation of pembrolizumab maintenance after 28 days) was chosen for part B of the study on the basis of the absence of any treatment-related grade 3 or worse adverse events nor dose-limiting toxicities in this cohort, compared with one grade 3 treatment-related adverse event in the schedule 2 cohort (grade 3 inflammatory arthritis) and schedule 3 cohort (grade 3 pneumonitis). Schedule 1 also demonstrated easier feasibility of administration.

**Figure 2:**
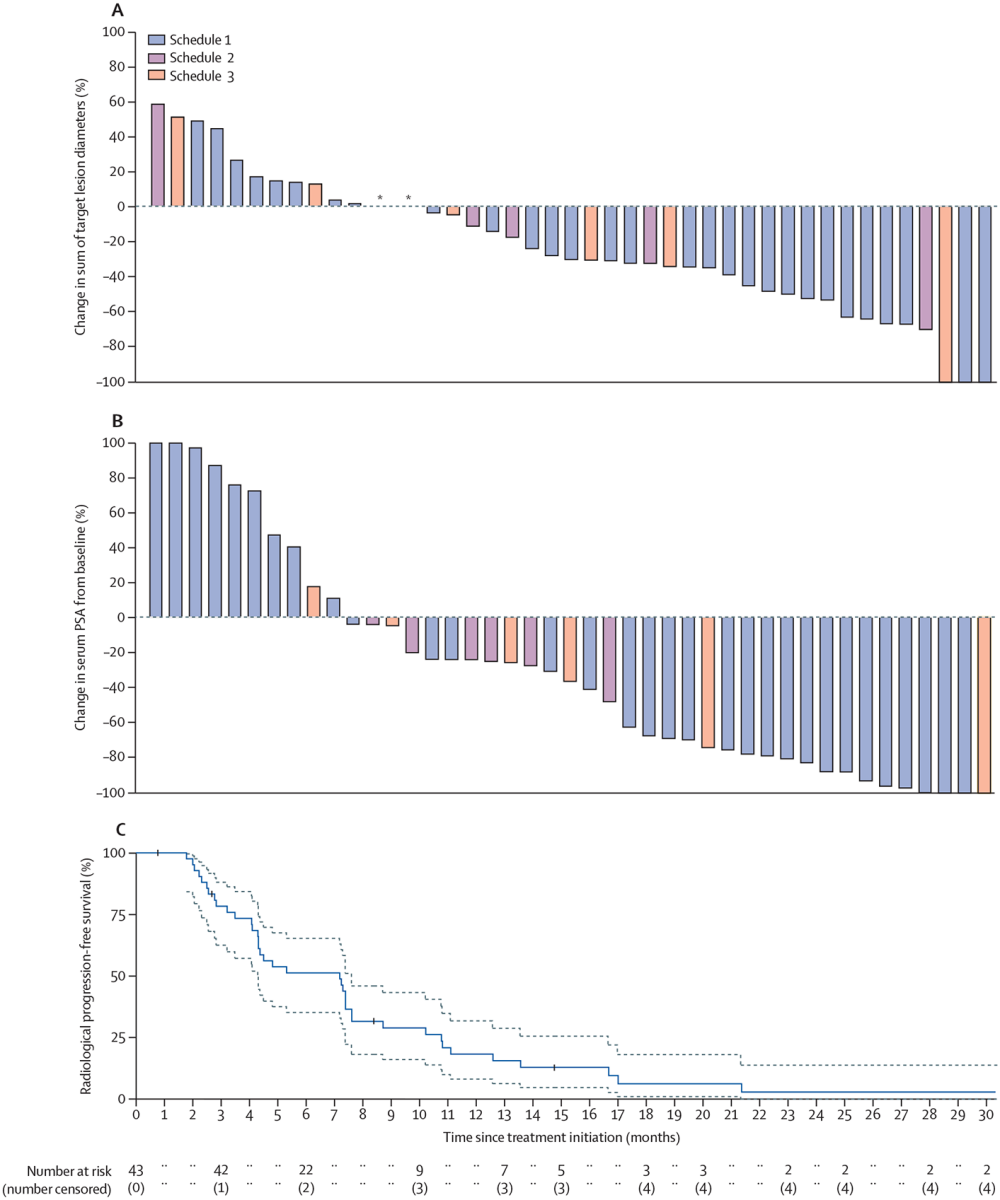
Maximum change in tumour size (A) and serum PSA (B) from baseline and radiographical progression-free survival (C) Maximal percentage change in tumour size was calculated as the sum of longest diameter of target lesions as per RECIST 1.1 criteria. In part A, one patient with 100% reduction in size of target lesions had non-target lesions present on follow-up imaging and thus was deemed to have partial response. In part C, grey lines show 95% CI lower and upper bounds. PSA=prostate-specific antigen. RECIST=Response Evaluation Criteria in Solid Tumours. *Two patients with no change in size of target lesions compared with baseline.

In part B, 14 (56%; 95 CI% 35–76) of 25 patients had a confirmed objective response (figure 2A). 14 (56%; 35–76) of 25 participants had a PSA50 response and four (16%; 5–36) had a PSA90 response (figure 2B).

In the overall study cohort, two (5%) of 43 patients had a grade 3 or worse treatment-related adverse event (grade 3 inflammatory arthritis in schedule 2 [n=1] and grade 3 pneumonitis in schedule 3 [n=1]). The most common treatment-related adverse events are shown in table 2. The most common treatment-related adverse events of any grade were fatigue (60%), nausea (40%), and joint pain (33%).

One serious adverse event (death due to aspiration pneumonia), no treatment-related serious adverse events, and no treatment-related deaths were observed. Overall, sixteen (37%) of 43 patients died; 15 were due to disease progression and one death was due to aspiration pneumonia. No delayed immune-related adverse events were observed that occurred more than 30 days after the final dose of pembrolizumab.

In the overall cohort (n=43), best overall response included two patients (5%) with complete response, 20 (47%) patients with confirmed partial response, 12 (28%) of patients with stable disease, and nine (21%) patients with progressive disease (figure 2A). The median duration of response was 8·1 months (range 6·0–10·0). Eleven (26%; 95% CI 14–41) of 43 patients had a durable objective response for longer than 6 months after the single priming dose of ^177^Lu-PSMA-617. Among the 11 patients with visceral metastases (liver metastases [n=4] n=4 lung [n=4], and other [n=3]), five (45%; 17–77) patients had an objective response, including two of four patients with liver metastases, and one of four patients with lung metastases.

19 (44%; 95% CI 29–59) of 43 patients had a PSA50 response and seven (16%; 5–27) of 43 patients had a PSA90 response (figure 2B). 38 (88%) of 43 patients had a PSA progression event; the median PSA progression-free survival was 3·5 months (95% CI 2·1–4·9).

39 (91%) of 43 patients had a radiographical progression event. The median progression-free survival was 6·9 months (95% CI 3·9–7·0), and 22 (51%; 35–67) of 43 patients were alive and progression free at 6 months (figure 2C). No marked differences were identified in tumour regression or PSA50 response (figure 2), nor significant differences in radiographical progression-free survival, by treatment schedule (data not shown). 14 (33%) of 43 patients had a symptomatic skeletal related event; the median time to SSRE was 24·7 months (20·7–not reached [NR]).

Subsequent anti-cancer therapies are summarised in the appendix (p 4). 16 (37%) of 43 patients died during follow-up. The median overall survival was 28·2 months (95% CI 20·4–40·0).

All patients who had an objective response had tumours that were microsatellite stable and had a low mutational burden on targeted somatic genomic sequencing (data not shown). Three patients harboured somatic pathogenic mutations in the homologous recombination deficiency pathway (*BRCA2, BRCA1*, and *PALB2*), of whom two (67%) had both PSA50 and objective response.

Seven patients had one or more PSMA-negative soft tissue lesion on PET at enrolment. Three (43%) of seven patients had clinical benefit (two with stable disease for >6 months, one with confirmed objective response).

**Figure 3:**
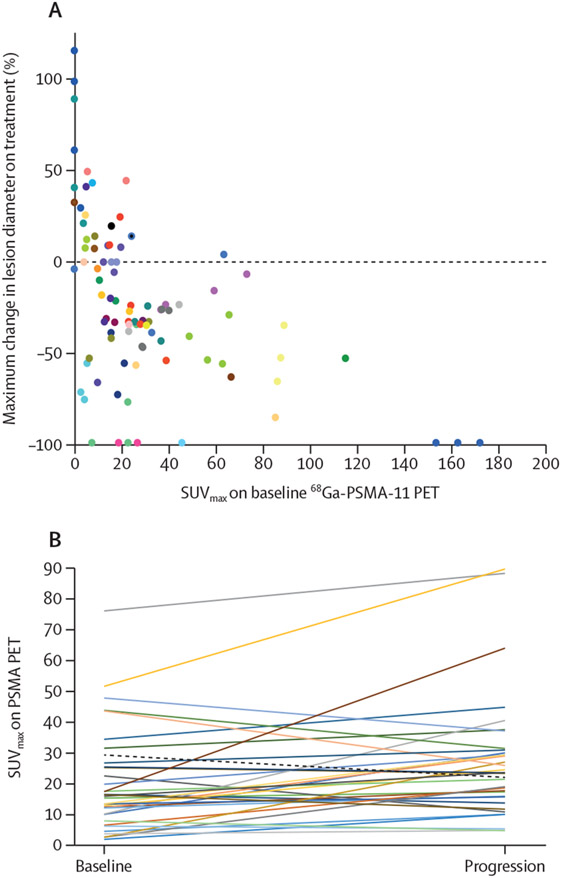
Percentage change in lesion diameter on conventional imaging by uptake on baseline PSMA PET (A) and patterns of uptake at baseline and at progression on PSMA PET (B) The association between maximum percentage change in measurable soft tissue lesions and uptake on baseline PSMA PET was investigated by fitting a linear mixed model to predict the maximal percentage change in the diameter of soft tissue lesions by cross-sectional imaging with baseline SUV_max_ on PSMA PET. Each colour represents lesions from individual patients enrolled in the study. PSMA=prostate-specific membrane antigen. PET=positron emission tomography. SUV_max_=maximum standardised uptake value.

91 evaluable metastatic soft tissue lesions were identified. Baseline maximum standardised uptake value (SUV_max_) on PSMA PET negatively correlated with percentage change in size of target lesions by cross-sectional imaging (β=−5·17×10^−3^, 95% CI [−8·69×10^−3^ to −1·64×10^−3^], t(48)=−2·95, p=0·01; standardised β coefficient=−0·39, 95% CI −0·66 to −0·12]; figure 3A). Among the subset of measurable soft tissue lesions with a SUV_max_ of 20 or less, 13 (29%; 16 to 44) of 45 lesions decreased by more than 30% in maximal diameter by cross-sectional imaging.

Follow-up PSMA PET scans were obtained at the time of radiographical or clinical progression in 18 (42%) of 43 patients, with a total of 34 evaluable soft tissue lesions. At progression, follow-up PSMA PET imaging showed PSMA expression was lower than at baseline (mean SUV_max_ at baseline 28·9; SUV_max_ at progression 21·7; p=0·02; figure 3B).

Eight patients received at least one dose of ^177^Lu-PSMA-617 as post-protocol therapy after progression on maintenance pembrolizumab, with a median interval of 19·2 months (range 13·1–36·1) between the first and second doses of ^177^Lu-PSMA-617. Five (63%; 95% CI 24–91) of eight patients had a PSA50 response and median progression-free survival was not reached (95% CI 4·5 months–NR) in the post-hoc analysis.

**Figure 4:**
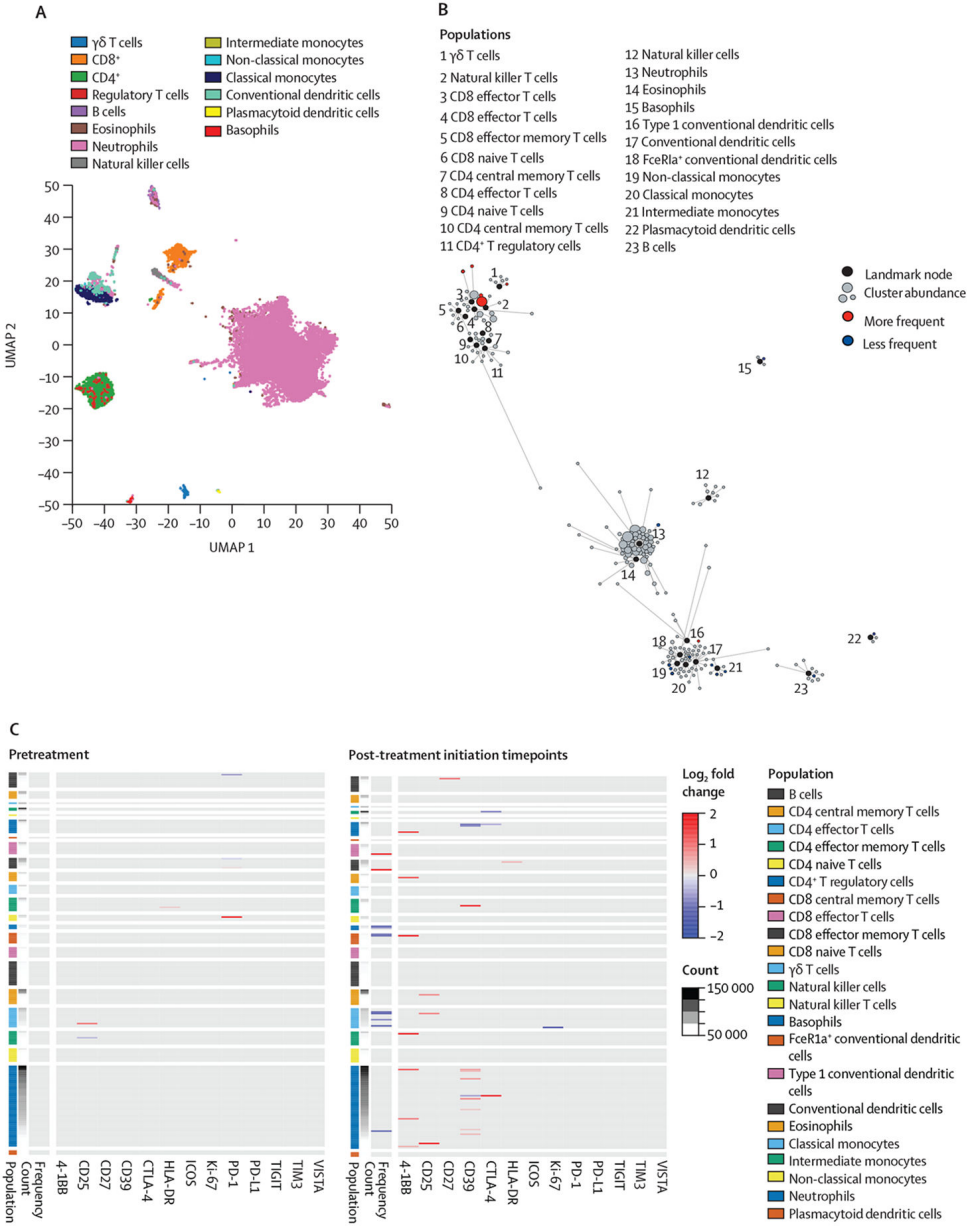
Immunomodulatory effects differ by clinical response Serial peripheral blood mononuclear cells obtained from patients were assessed by mass cytometry. (A) A UMAP plot of cell populations assessed from whole blood samples obtained from the full cohort. (B) Statistical scaffolding of the relationships between cell clusters identified in samples obtained from patients with a response and those who had no response to treatment. (C) Heatmaps summarising log_2_-fold changes resulting from statistical scaffold analysis of cell cluster frequency and functional markers (4-1BB, CD25, CD27, CD39, CTLA-4, HLA-DR, ICOS, Ki-67, PD-1, PD-L1,TIGIT, TIM3 and VISTA) are shown for pre-treatment (left panel) and at all on-treatment timepoints (right panel) in patients with a response and those with no response. Each bar represents a cell cluster that has been labeled according to the nearest landmark node and ordered by cell count abundance. The colour coding represents clusters that showed a significant difference (p<0·05) in the log_2_ fold change in patients with a response versus no response. The colour intensity is proportional to the log_2_ fold change and is capped at 2 and −2. UMAP=uniform manifold approximation and projection. 4-1BB=CD137. CTLA-4=cytotoxic T-lymphocyte-associated protein 4. HLA-DR=human leukocyte antigen DR. ICOS=inducible T-cell co-stimulator. TIGIT=T-cell immunoreceptor with Ig and ITIM domains. TIM3=T-cell immunoglobulin and mucin domain-containing protein 3. VISTA=V-domain immunoglobulin suppressor of T-cell activation.

An exploratory analysis of circulating immune cell subsets from patients enrolled in part A (n=18) showed that pembrolizumab reduced PD-1 staining in T-cell populations (appendix p 9). At all post-treatment timepoints, patients who had an objective response had higher cell cluster frequencies of CD8^+^ effector cells, CD8^+^ effector memory cells, γδT cells, and natural killer T cells than non-responders (figure 4A, B). Patients who had an objective response had lower cell cluster frequencies of basophils, FceR1a^+^ conventional dendritic cells, classical monocytes, neutrophils, and plasmacytoid dendritic cells than did non-responders (figure 4C).

## Discussion

We assessed three dosing schedules of a single priming dose of ^177^Lu-PSMA-617 relative to initiation of maintenance pembrolizumab in patients with metastatic castration resistant prostate cancer. Administration of ^177^Lu-PSMA-617 followed by maintenance pembrolizumab was selected as the recommended phase 2 schedule. Using this approach, significant anti-tumour activity was observed with 56% of patients a subset of whom had durable response, and a favourable toxicity profile with minimal haematological effect of treatment and a manageable immune related adverse events.

The patient population included in this study had molecularly unselected metastatic castration-resistant prostate cancer without genomic evidence of tumours with a high mutational burden or microsatellite instability, a setting in which responses are expected in fewer than 10% of patients given pembrolizumab alone.^3^ Supporting the potential effect of immunological priming with targeted radioligand therapy, we found that clinical responders had an increase in circulating CD8 T-cell populations with a concomitant decrease in immunosuppressive myeloid cells when compared with non-responders.

The combination is further supported by the favourable results from the PRINCE study,^23^ in which ^177^Lu-PSMA-617 was administered for up to six cycles in combination with pembrolizumab. In this study, the objective response rate was 70% and median progression-free survival was 11·2 months, potentially an improvement compared with ^177^Lu-PSMA-617 given at the same dose or schedule without concomitant immunotherapy.^18^ There are important differences between PRINCE and the current study, including repeated versus single priming dosing of ^177^Lu-PSMA-617, stringent selection criteria based on dual PSMA and fluorodeoxyglucose PET imaging, and inclusion of patients without measurable soft tissue disease in the PRINCE study.^23^ However, the aggregate results from both non-randomised studies provide preliminary support for the combination of targeted radioligand therapy plus immune checkpoint inhibition in patients with metastatic castration-resistant prostate cancer.

The current study design differs substantially from the phase 3 VISION study.^18^ Compared with patients in the current study, the enrolled patients in the VISION trial with metastatic castration-resistant prostate cancer were more heavily pre-treated, including at least one androgen signalling inhibitor and one or two previous lines of taxane chemotherapy. Additionally, in VISION up to six successive doses of ^177^Lu-PSMA-617 were administered. Despite these important differences, and with the caveats of cross-trial comparisons, the objective response rate was similar to that reported in VISION. However, progression-free survival was numerically longer in VISION than in the current study, perhaps owing to the use of repeated doses of ^177^Lu-PSMA-617. In contrast, the safety data from the current study indicate the potential for reduced toxicity compared with repeated doses of radioligand therapy.

The optimal dose and schedule for immunogenic priming with ^177^Lu-PSMA-617 remains to be defined. In the current study, no substantial differences were identified with regard to changes in circulating immune cell subsets between the three schedules evaluated; however, the small sample size of each cohort precludes definitive evaluation. The administration of radiation followed by immune checkpoint inhibition is consistent with the approach applied in multiple previous studies in both prostate and other solid tumour malignancies.^13,16^ We chose to use the approved dose of ^177^Lu-PSMA-617 of 7·4 Gbq; however, the optimal dose of targeted radioligand treatment to elicit an immunogenic response remains to be elucidated.

There is substantial heterogeneity in PSMA expression and uptake on PSMA PET in the metastatic castration-resistant prostate cancer setting, which narrows the scope of who might benefit from targeted radioligand therapy.^18,24-26^ In the current study, we used a more liberal PET selection strategy than previous studies of ^177^Lu-PSMA-617 (VISION and PRINCE) and observed anti-tumour activity in lesions with low or negative uptake. These data provide support for the hypothesis that the combination radioimmunotherapeutic strategies might extend therapeutic benefit in patients with low surface antigen expression.

Our study had limitations including the non-randomised design, which precludes the ability to ascertain the individual contribution of pembrolizumab and ^177^Lu-PSMA-617. The expected outcomes following a single dose of ^177^Lu-PSMA-617 alone have not been prospectively evaluated, and it is plausible that durable responses would be observed following a single dose without maintenance pembrolizumab. Stringent selection based on dual PSMA and FDG PET imaging might represent a more optimal approach to exclude patients who are less likely to respond to ^177^Lu-PSMA-617, and we mighty have observed greater anti-tumour activity had such an approach been employed. We did not acquire serial PSMA PET during study treatment; understanding the temporal evolution of PSMA expression over time might be helpful in delineating resistance mechanisms. Considering that this was a molecularly unselected patient population, it remains to be investigated whether greater activity might be observed in genomically-selected subsets of patients with metastatic castration-resistant prostate cancer.

A phase 2 study is planned combining maintenance pembrolizumab with repeated doses of ^177^Lu-PSMA-617 delivered at variable intervals dependent on PSA and PSMA PET-defined progression to prime and re-prime the anti-tumour response (NCT05766371).

## Supplementary Material

Supplementary Appendix

## Figures and Tables

**Table 1:** Baseline characteristics

	Participants (n=43)
Age, years	71 (64–77)
Race	
White	35 (81%)
Black	5 (12%)
Asian	2 (5%)
Native American	1 (2%)
Previous androgen signalling inhibitor therapy	
Abiraterone	12 (30%)
Second generation androgen receptor antagonist (enzalutamide, apalutamide, or darolutamide)	9 (18%)
Both	22 (51%)
Previous docetaxel for castration-sensitive disease	6 (14%)
Visceral metastases	
Any organ	11 (30%)
Liver	4 (9%)
Homologous recombination deficiency mutation status (germline or somatic)	
Wildtype	37 (86%)
Mutant	3 (7%)*
Unknown	3 (7%)
Microsatellite status	
Stable	43 (100%)
High	0
Laboratory parameters at study entry	
PSA, ng/mL	31·8 (12·7–108)
Alkaline phosphatase, U/L	96 (66–170)
Albumin, g/L	3·8 (3·7–4·1)
Haemoglobin, g/dL	12·4 (11·1–13·2)
ECOG performance status	
0	16 (37%)
1	27 (63%)
Type of progression at study entry†	
PSA progression only	10 (23%)
Radiographical progression with or without PSA progression	33 (77%)

Data are median (IQR), or n (%). RECIST=Response Evaluation Criteria in Solid Tumours. ECOG=Eastern Cooperative Oncology Group. PSA=prostate-specific antigen. *One patient had a *BRCA1* mutation, one had a *BRCA2 mutation*, and one patient had a *PALB2* mutation. †According to Prostate Cancer Working Group 3 criteria.

**Table 2:** Treatment-emergent adverse events

	Part A: schedule 1 (n=6)				Part A: schedule 2 (n=6)				Part A: schedule 3 (n=6)				Part B (n=25)				Overall study cohort (n=43)			
	Grade 1–2	Grade 3	Grade 4	Grade 5	Grade 1–2	Grade 3	Grade 4	Grade 5	Grade 1–2	Grade 3	Grade 4	Grade 5	Grade 1–2	Grade 3	Grade 4	Grade 5	Grade 1–2	Grade 3	Grade 4	Grade 5
Fatigue	1 (17%)	0	0	0	1 (17%)	0	0	0	4 (67%)	0	0	0	20 (80%)	0	0	0	26 (60%)	0	0	0
Nausea	2 (33%)	0	0	0	2 (33%)	0	0	0	4 (67%)	0	0	0	9 (36%)	0	0	0	17 (40%)	0	0	0
Joint pain or arthritis	2 (33%)	0	0	0	1 (17%)	1 (17)	0	0	1 (17%)	0	0	0	9 (36%)	0	0	0	13 (30%)	1 (2%)	0	0
Decreased appetite	2 (33%)	0	0	0	2 (33%)	0	0	0	3 (50%)	0	0	0	3 (12%)	0	0	0	10 (23%)	0	0	0
Xerostomia	2 (33%)	0	0	0	1 (17%)	0	0	0	1 (17%)	0	0	0	6 (24%)	0	0	0	9 (21%)	0	0	0
Diarrhoea	0	0	0	0	2 (33%)	0	0	0	2 (33%)	0	0	0	5 (20%)	0	0	0	7 (16%)	0	0	0
Hypothyroidism	1 (17%)	0	0	0	0	0	0	0	0	0	0	0	5 (20%)	0	0	0	6 (14%)	0	0	0
Rash	1 (17%)	0	0	0	0	0	0	0	0	0	0	0	1 (4%)	0	0	0	3 (7%)	0	0	0
Pruritus	0	0	0	0	1 (17%)	0	0	0	1 (17%)	0	0	0	1 (4%)	0	0	0	3 (7%)	0	0	0
Dry eyes	0	0	0	0	0	0	0	0	0	0	0	0	3 (12%)	0	0	0	3 (7%)	0	0	0
Vomiting	0	0	0	0	1 (17%)	0	0	0	0	0	0	0	1 (4)	0	0	0	2 (5%)	0	0	0
Pneumonitis	0	0	0	0	0	0	0	0	0	1 (17%)	0	0	1 (4%)	0	0	0	1 (2%)	1 (2%)	0	0
Thrombocytopenia	1 (17%)	0	0	0	0	0	0	0	0	0	0	0	0	0	0	0	1 (2%)	0	0	0
Anaemia	0	0	0	0	1 (17%)	0	0	0	0	0	0	0	0	0	0	0	1 (2%)	0	0	0
Neutropenia	0	0	0	0	1 (17%)	0	0	0	0	0	0	0	0	0	0	0	1 (2%)	0	0	0

Data are n (%).

## Data Availability

Aggregate clinical data will be made available on ClinicalTrials.gov. De-identified individual participant data, together with a data dictionary defining each field in the set, will be made available to other researchers on request to the corresponding author. Trial documentation including the protocol are available in the appendix. The University of California, San Fransisco supports wider dissemination of information from the research it conducts and increased cooperation between investigators. Trial data are obtained, managed, stored, shared, and archived according to The University of California, San Fransisco institutional guidelines to ensure the enduring quality, integrity, and utility of the data. Formal requests for data sharing are to be made to the corresponding author (RA) describing the nature of the proposed research and extent of data requirements. Data recipients are required to enter a formal data sharing agreement, which describes the conditions for release and requirements for data transfer, storage, archiving, publication, and intellectual property. Requests are reviewed by the study investigators in terms of scientific merit and ethical considerations, including patients’ consent. Data sharing is undertaken if proposed projects have a sound scientific or patients’ benefit rationale, as agreed by the study investigators. Restrictions relating to patients’ confidentiality and consent will be limited by aggregating and anonymising identifiable patients’ data. Additionally, all indirect identifiers that could lead to deductive disclosures will be removed in line with The University of California, San Fransisco data sharing policy.

## Research in context

### Evidence before this study

We searched PubMed for articles published between Jan 1, 2000, and March 1, 2023, for preclinical and clinical studies on the combination of priming doses of radiation plus immunotherapy in prostate cancer and other cancers using the search terms: "cancer" AND "radiation" AND "immunotherapy". No language restrictions were applied. Preclinical studies have shown that the combination of radiation with immunotherapy can enhance anti-tumour efficacy in multiple cancer models. A phase 3 study in patients with metastatic castration-resistant prostate cancer using a single dose of external beam radiation followed by ipilimumab demonstrated that a subset of patients had durable disease control and long-term survival with extended follow-up.

### Added value of this study

To our knowledge, this is the first phase 1 trial to assess the activity of a single priming dose of targeted radioligand therapy coupled with immune checkpoint inhibition in patients with metastatic prostate cancer. We observed durable objective responses in a subset of patients, in whom we observed upregulation of circulating T-cell subsets and downregulation of immunosuppressive myeloid precursors, including patients with tumours with low expression of prostate-specific membrane antigen.

### Implications of all the available evidence

The current study provides robust clinical support for the immunogenic effect of radiation and provides the impetus to further evaluate immune checkpoint inhibition in patients with metastatic prostate cancer as a maintenance treatment following priming with targeted radioligand therapy. The ability to re-treat with lutetium-177 [^177^Lu]-PSMA-617 at disease progression might provide an opportunity to re-prime antitumor immunity and extend the duration of response, and would need further investigation.
