# Supplementary material for: Single-dose 177Lu-PSMA-617 followed by maintenance pembrolizumab in patients with metastatic castration-resistant prostate cancer: an open-label, dose-expansion, phase 1 trial
Source: Lancet Oncol. Author manuscript; Available in PMC 2023 Nov 23. (PMC10667020; doi:10.1016/S1470-2045(23)00451-5)
Supplement: Supplementary Appendix [file NIHMS1943506-supplement-Supplementary_Appendix.pdf]

Supplementary Methods

Whole blood samples were exposed to red blood cell lysis buffer, stained with cisplatin, washed, fixed with paraformaldehyde (PFA), and cryopreserved. Samples were thawed in batch, barcoded with palladium isotopes and mixed for multiplex assessment by mass cytometry by time-of-flight (CyTOF). The combined barcoded samples were stained extracellularly and intracellularly with heavy metal-labeled antibodies in preparation for acquisition by CyTOF. Stained cells were then washed and analyzed on a mass cytometer (Helios, Fluidigm). Acquired data was pre-processed using the premessa R-package (github.com/ParkerICI/premessa) to utilize bead-base normalization, bead removal, de-barcode samples and edit the panel used for staining. PBMCs that were singlets, live (intercalator+ cisplatin-), CD45+, and RBC- and platelet-free (CD61- CD235ab-) were extracted as new FCS files by manual gating in Cytobank (Beckman Coulter) (**Figure 4A**), and subsequently analyzed by unsupervised clustering into 200 clusters and statistical scaffold R package (https://github.com/SpitzerLab/statisticalScaffold) to assess for significant changes in the immune landscape in response to treatment or based on patient clinical responses (Spitzer et al Cell 2017). Clusters were arranged around landmark nodes, which are individual cell types conventionally manually gated and extracted as fcs files in Cytobank, based on similarity in force-directed scaffold maps. Cluster frequencies and boolean expression for functional markers for each cluster were passed through the Significance Across Microarrays algorithm and results were formulated into the Scaffold maps for visualization. For scaffold heatmaps, fold change, significance, cell count, and nearest landmark node data were extracted from the outputs of the Scaffold analysis

using a custom script. The heat map was created in R using pheatmap (https://CRAN.R-project.org/package=pheatmap), with log2 fold change capped at -2 to 2. Mass cytometry data followed the gating schema is shown in **Supplemental Figure 1** for all analyses.

A linear mixed model was utilized (estimated using REML and nlminb optimizer) to predict the maximal percent change in the diameter of soft tissue lesions by cross-sectional imaging with baseline SUV_{max} on PSMA PET. The model included baseline SUV_{max} as random effect (formula: ~Baseline.SUV | Patient.ID). The model's total explanatory power was substantial (conditional $R^2 = 0.81$) and the part related to the fixed effects alone (marginal R^2) was 0.17. Standardized parameters were obtained by fitting the model on a standardized version of the dataset. 95% confidence intervals and p-values were computed using a Wald t-distribution approximation.

Supplementary Table 1

Supplemental Table 1

Heavy metal tags and antibodies for CyTOF panel

Metal tag:	Antibody Target:	Catalog #:	Manufacturer:
	VISTA-PE	18946S	Cell Signaling Technology
89Y	CD45	304002	Biolegend
113In	CD61	336402	Biolegend
113In	CD235ab	3066020	Biolegend
139La	CD33	303402	Biolegend
140Ce	CD15	323002	Biolegend
141Pr	CD3	300402	Biolegend
142Nd	CD19	302202	Biolegend
144Nd	CD11b	3011302	Biolegend
145Nd	CD4	3002502	Biolegend
146Nd	CD8a	555384	Biolegend
147Sm	CD11c	337202	Biolegend
148Nd	CD14	301802	Biolegend
149Sm	CD127	351302	Biolegend
150Nd	FceRIa	334602	Biolegend
151Eu	CD123	3026002	Biolegend
152Sm	γδTCR	20210406	UCSF-PFCC
153Eu	CD45RA	304102	Biolegend
154Sm	TIM3	345010	Biolegend
155Gd	TIGIT	16-9500-82	Invitrogen
156Gd	PD-L1 (CD274)	329716	Biolegend
158Gd	CD27	356401	Biolegend
159Tb	CD137 (4-1BB)	309802	Biolegend
160Gd	Tbet	644802	Invitrogen
161Dy	CD152 (CTLA-4)	2053204	Invitrogen
162Dy	FoxP3	14-4776-37	Invitrogen
163Dy	CD31	303102	Biolegend
164Dy	CD39	328202	Biolegend
165Ho	PE	801827	Fluidigm
166Er	CD141 (BDCA3)	344102	Biolegend
167Er	CCR7 (CD197)	353256	Biolegend
168Er	Ki-67	350502	Biolegend
169Tm	CD25	555430	Biolegend
170Er	BDCA1	331502	Biolegend
171Yb	CD68	333802	Biolegend
172Yb	CD163	333602	Biolegend
173Yb	ICOS	313512	Biolegend
174Yb	HLA-DR	307602	Biolegend
175Lu	PD-1	329902	Biolegend
176Yb	CD56	559043	BD Biosciences
209Bi	CD16	302002	Biolegend

Heavy metals purchased from Fluidigm

Staining reagents

Reagent:	Catalog #:	Manufacturer:
autoMACS Running Buffer	130-091-221	Miltenyi Biotec
Cell-ID Intercalator-Ir (500uM)	201192B	Fluidigm
Human TruStain FcX	422302	Biolegend
Maxpar 10x Barcode Perm Buffer	201057	Fluidigm
Maxpar Perm-S Buffer	201066	Fluidigm
Paraformaldehyde (16%)	15710	Electron Microscopy Sciences

Supplementary Table 2: Subsequent Systemic Anti-Cancer Therapy

Therapy (%)	Study Cohort (N = 43)
Docetaxel	28 (65)
Cabazitaxel	14 (33)
Carboplatin	13 (30)
Enzalutamide	7 (16)
^{177}Lu-PSMA-617	7 (16)
None	6 (14)
Abiraterone	5 (12)
Olaparib	2 (5)
Radium-223	2 (5)
Sipuleucel-T	2 (5)
Darolutamide	1 (2)

Supplementary Figure Legends

Supplementary Figure 1. Gating schema used for mass cytometry data analysis. The gating strategy used to remove residual beads, dead cells and cellular debris acquired from samples. The strategy was used to identify key cell populations that were used for downstream analysis with manual gating for functional markers and for landmark nodes used for statistical scaffold analysis.

Supplementary Figure 2. Duration of treatment and patient disposition. The three patients with mutations in the homologous recombination repair pathway are indicated in the left column.

Supplementary Figure 3. Differential immune modulation is seen across three treatment schedules with combination treatment. Serial PBMCs from patients were assessed by mass cytometry. (A) Statistical scaffold was used to compare the cell frequencies pre vs post treatment timepoints is shown. Landmark nodes are shown in black. Clusters with significantly increased abundances are shown in red while clusters with decreased abundance are shown in blue. (B) Heatmaps summarizing $\log_2$ fold changes resulting from statistical scaffold analysis of cell cluster frequency (left most column) and functional markers (4-1BB, CD25, CD27, CD39, CTLA-4, HLA-DR, ICOS, Ki-67, PD-1, PD-L1, TIGIT, TIM3 and VISTA) when comparing pretreatment timepoint (TP1) vs timepoint 2 (TP2, upper row) and vs timepoint (TP3, lower row) are shown for the 3 arms. (C) Gating analysis of the analysis for PD-1 on CD4+ (upper row) and CD8+ T cells (lower row), are shown from a representative sample from each arm between pretreatment (TP1) and follow up treatment timepoint (TP2).

Supplementary Figure 4. CD39 expression is differentially modulated on neutrophils between responders and non-responders with treatment. Flow cytometry analysis for CD39 expression on neutrophils (gated on CD3-, CD15+ and CD16+) are shown from a representative responder (top row) and a representative non-responder (bottom row) across study timepoints.

Supplementary Figure 1

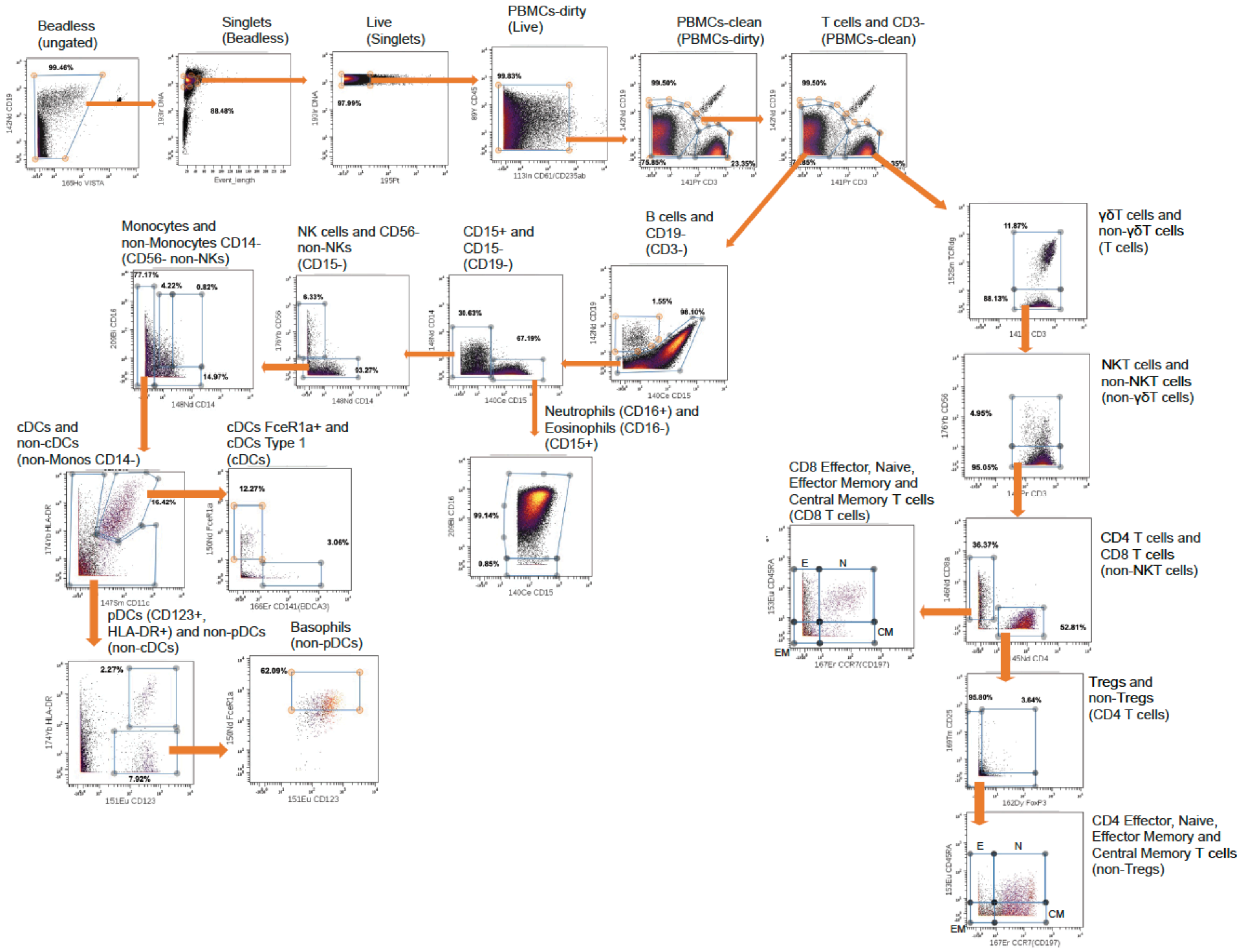

Supplementary Figure 2

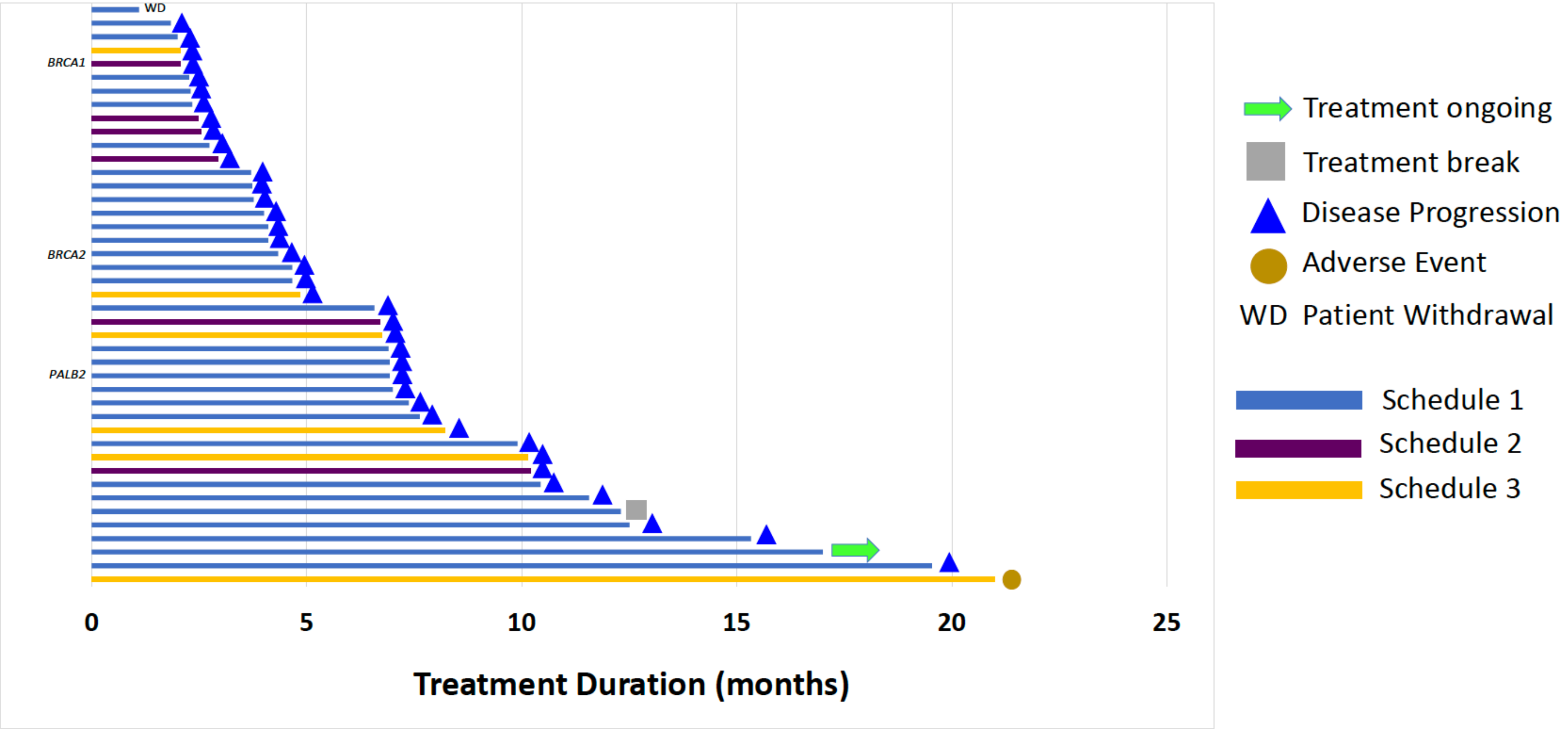

Supplementary Figure 3

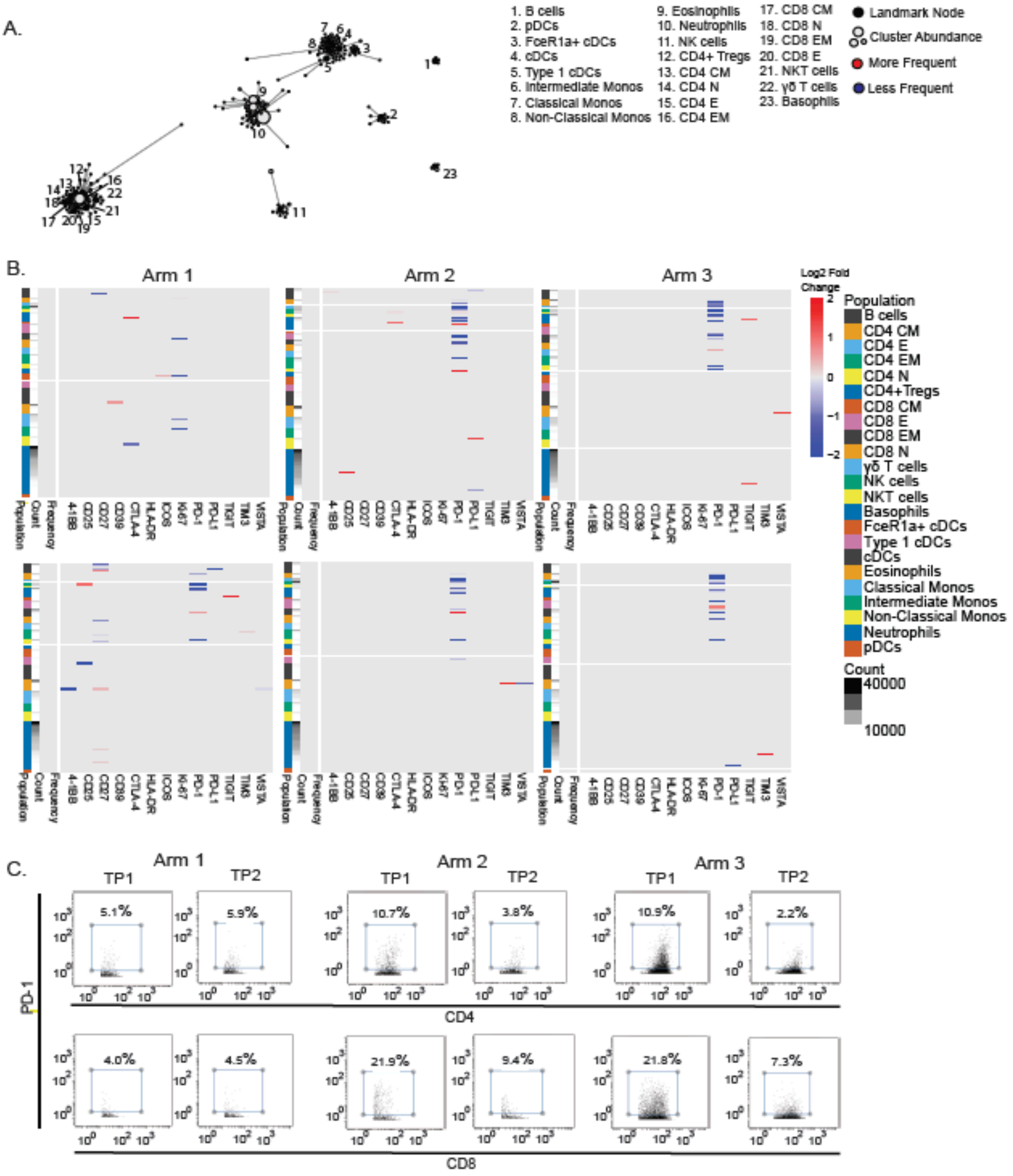

Supplementary Figure 4

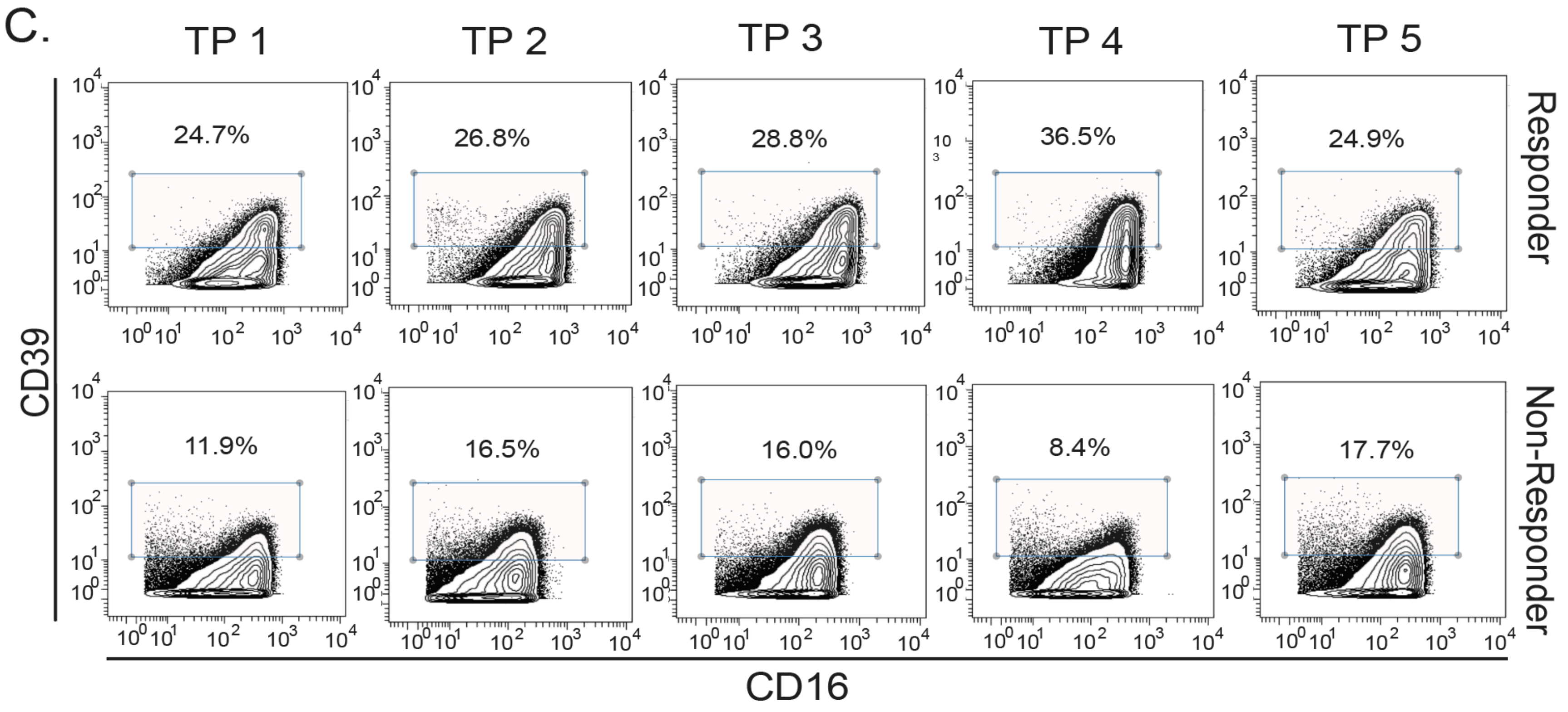

Immunogenic Priming with PSMA-Targeted Radioligand Therapy in Advanced Prostate Cancer: A Phase 1b Study of ^{177}Lu-PSMA-617 in Combination with Pembrolizumab

Protocol Number: CC# 185511

Study Drugs:
^{177}Lu-PSMA-617
Pembrolizumab

Version Number: 2.0

Version Date: 04/19/2021

IND Number: 139715

Principal Investigator
Rahul Aggarwal, MD
University of California San Francisco
550 16th Street
San Francisco, CA 94158
Telephone: 415-353-9278
E-mail: Rahul.Aggarwal@ucsf.edu

Co-Principal Investigator (Sponsor-Investigator)
Thomas Hope, MD
University of California San Francisco
185 Berry Street, Lobby 6, Suite 350
San Francisco, CA 94132
Telephone: 415-353-9437
E-mail: Thomas.Hope@ucsf.edu

Revision History

Version 1.0	04/11/2018
Version 1.1	05/03/2018
Version 1.2	06/19/2018
Version 1.3	09/08/2018
Version 1.4	10/01/2018
Version 1.5	05/06/2019
Version 1.6	11/21/2019
Version 1.7	01/03/2020
Version 1.8	06/01/2020
Version 1.9	10/06/2020
Version 2.0	04/19/2021

Protocol Signature Page

Protocol No.: 185511 **Version Date:** 04/19/2021

1. I agree to follow this protocol version as approved by the UCSF Protocol Review Committee (PRC), Institutional Review Board (IRB), and Data Safety Monitoring Committee (DSMC).
2. I will conduct the study in accordance with applicable IRB requirements, Federal regulations, and state and local laws to maintain the protection of the rights and welfare of study participants.
3. I certify that I, and the study staff, have received the requisite training to conduct this research protocol.
4. I have read and understand the information in the Investigators' Brochure (or Manufacturer's Brochure) regarding the risks and potential benefits. I agree to conduct the protocol in accordance with Good Clinical Practices (ICH-GCP), the applicable ethical principles, the Statement of Investigator (Form FDA 1572), and with local regulatory requirements. In accordance with the FDA Modernization Act, I will ensure the registration of the trial on the www.clinicaltrials.gov website.
5. I agree to maintain adequate and accurate records in accordance with IRB policies, Federal, state and local laws and regulations.

UCSF Principal Investigator / Study Chair

____
Printed Name

____________________________________ _______________
Signature Date

Abstract

Title	Immunogenic Priming with ^{177}Lu-PSMA Targeted Therapy in Advanced Prostate Cancer: A Phase 1b Study
Patient population	Patients with metastatic castration resistant prostate cancer (mCRPC) will be enrolled. All patients must have measurable disease by RECIST 1.1 criteria, with at least 3 lesions which are PSMA-avid on ^{68}Ga-PSMA-11 PET imaging.
Rationale for Study	Despite recent advances in the treatment of mCRPC, including the approval of novel agents such as sipuleucel-T, abiraterone acetate, enzalutamide, and radium-223, patients with mCRPC have a poor prognosis and many eventually succumb to their disease. Patients with abiraterone and/or enzalutamide refractory mCRPC represents a particularly challenging subgroup of patients who have limited effective treatment options other than chemotherapy.<br><br>Prostate-Specific Membrane Antigen (PSMA) is a cell surface protein overexpressed on most prostate cancer cells, especially in advanced stage disease. The safety and efficacy of ^{177}Lu radiolabeled PSMA has been reported in several studies, including a recent prospective phase 2 study (Hofman et al. 2017). In patients with PSMA-avid metastatic disease, the PSA response proportion and objective response rate by RECIST 1.1 range from 40-60%, in heavily pre-treated patients. However the response duration is short, and therefore novel treatment strategies are needed to maintain and extend the duration of response achieved.<br><br>We propose to utilize immune checkpoint inhibition with pembrolizumab as a means to maintain anti-tumor response. We leverage a robust body of literature indicating that radiotherapy induces an immunogenic response, in the form of induction of infiltrating T cells, broadening of the T cell repertoire, and induction of PD-L1 expression, facilitating subsequent treatment with immune checkpoint inhibitor.<br><br>In the current study we will obtain the preliminary safety and efficacy data of ^{177}Lu-PSMA-617 radioligand therapy (RLT) in combination with pembrolizumab in patients with metastatic castration resistant prostate cancer. Favorable results with respect to toxicity and preliminary evidence of efficacy would form the basis for a subsequent definitive randomized study of the treatment combination in PSMA-expressing metastatic castration-resistant prostate cancer.
Primary Objectives	**Part A:** To determine the recommended phase 2 dose and schedule of ^{177}Lu-PSMA-617 in combination with pembrolizumab in patients with mCRPC.<br><br>**Part B (Dose Expansion):** To determine the objective response rate by RECIST 1.1 criteria.

Secondary Objectives	1. To characterize the safety profile of the combination<br>2. To determine the median duration of response by RECIST 1.1 criteria.<br>3. To determine the proportion of patients who experience ≥ 50% decline from baseline in serum PSA.<br>4. To determine the median PSA progression-free survival.<br>5. To determine the median time to symptomatic skeletal related event.<br>6. To determine the 6 month radiographic progression-free survival rate and median radiographic progression-free survival.<br>7. To determine the median overall survival.
Correlative Objectives	1. To assess the lesion-specific response rate by baseline PSMA avidity on ^{68}Ga-PSMA-11 PET.<br>2. To quantify the change from baseline in T cell repertoire, circulating T cell subsets, tumor infiltrating lymphocytes, and tumor PD-L1 expression by immunohistochemistry after one priming dose of Lu-PSMA RLT.<br>3. To explore the relationship between timing of the ^{177}Lu-PSMA-617 priming dose with initiation of pembrolizumab with respect to immunologic, safety, and efficacy outcomes.<br>4. To descriptively characterize the patterns of uptake on ^{68}Ga-PSMA-11 PET at the time of disease progression.<br>5. To explore relationship between tumor genomic profile with clinical outcomes including response rate and progression-free survival.<br>6. To explore the relationship between tumor dosimetry with objective response.

Study Design	This is a single-center Phase 1b open-label, study of PSMA-targeted radionuclide therapy with ^{177}Lu-PSMA-617 in combination with pembrolizumab in patients with mCRPC who have previously progressed on at least one prior androgen pathway inhibitor (e.g. abiraterone, enzalutamide, apalutamide). Patients must have at least three metastatic lesions which are PSMA-avid on baseline ^{68}Ga-PSMA-11 PET imaging.<br><br>In **Part A** of the study, patients will be enrolled sequentially into one of three treatment schedules with respect to timing of ^{177}Lu-PSMA-617 in relation to first dose of pembrolizumab (N = 6 patients per schedule):<br><br>**Schedule 1)** Priming dose of ^{177}Lu-PSMA-617 administered on Cycle 1 Day 1 followed by pembrolizumab 200 mg IV q 21 days starting Cycle 2 Day 1.<br><br>**Schedule 2)** Priming dose of ^{177}Lu-PSMA-617 administered concurrently with initiation of pembrolizumab on Cycle 1 Day 1.<br><br>**Schedule 3)** Priming dose of ^{177}Lu-PSMA-617 administered on Cycle 1 Day 1 following initiation of pembrolizumab on Cycle 1 Day -21.<br><br>The dose-limiting toxicity window will be Cycle 1 + Cycle 2 (i.e. 6 weeks following C1D1 administration of Lu-PSMA-617 across) in all three Schedule cohorts.<br><br>In Part A of the study, 6 patients will be enrolled to Schedule 1, then 6 patients to Schedule 2, and finally 6 patients will be enrolled to Schedule 3.<br><br>The starting dose of ^{177}Lu-PSMA-617 will be 7.4 +/- 15% GBq, which is the recommended phase 3 dose of the agent. If the DLT frequency attributable to Lu-PSMA exceeds 33% at any point after 6 evaluable patients have been enrolled in Part A, Dose Level -1 of ^{177}Lu-PSMA-617 will be investigated (4 Gbq +/- 15%) for the remainder of patients enrolled on study.<br><br>Using the data obtained in Part A, the recommended phase 2 dose and schedule of ^{177}Lu-PSMA-617 will be determined by the Principal Investigator using a combination of safety, efficacy, and immunologic effects of treatment from the first two cycles (and additional cycles of treatment when available).<br><br>In **Part B** of the study, an additional 25 patients will be enrolled at the recommended phase 2 dose and schedule to obtain additional safety, efficacy, and immunologic data pertaining to the treatment combination.

Number of patients	Approximately 18 patients will be enrolled during Part A of the study. An additional 25 patients will be enrolled in Part B.<br><br>Total sample size: 43<br><br>Part A: N ~ 18 patients (6 patients per Schedule; 3 Schedules to be evaluated)<br><br>Part B: N ~ 25 patients
Duration of Therapy	Patients will be treated until disease progression per PCWG3/RECIST 1.1 criteria, confirmed by repeat scans performed ≥ 4 weeks later, unacceptable toxicity, or patient withdrawal, whichever occurs first.
Duration of Follow up	Patients will undergo safety follow up visit approximately 30 days and 90 days following end of treatment visit.
Duration of study	The study is anticipated to accrue 2-3 patients per month for an estimated accrual period of 21 months. The study is expected to reach completion for analysis of primary and secondary endpoints approximately 36 months from first patient enrolled.
Study Drugs	^{177}Lu-PSMA-617 is a PSMA-targeted radioligand that will be produced under Current Good Manufacturing Practice (CGMP) regulations. The priming dose of ^{177}Lu-PSMA-617 will be 7.4 +/- 15% GBq, which is the recommended phase 3 dose of the agent.<br><br>The planned dose schedules to be evaluated in Part A of the study include:<br><br>**Schedule 1)** Priming dose of ^{177}Lu-PSMA-617 administered on Cycle 1 Day 1 followed by pembrolizumab 200 mg IV q 21 days starting Cycle 2 Day 1.<br><br>**Schedule 2)** Priming dose of ^{177}Lu-PSMA-617 administered concurrently with initiation of pembrolizumab on Cycle 1 Day 1.<br><br>**Schedule 3)** ^{177}Lu-PSMA-617 administered on Cycle 1 Day 1 following initiation of pembrolizumab on Cycle 1 Day -21.<br><br>The cumulative safety data, including safety data beyond Cycle 2 where available, immunologic priming effects, along with efficacy and pharmacodynamic data, will be utilized to determine the recommended schedule of ^{177}Lu-PSMA-617 in combination with pembrolizumab.

Safety Assessments	Safety will be assessed by reviewing adverse events (AEs), laboratory evaluations, and by physical examination. The NCI CTCAE v4.0 will be used. All AEs and serious AEs (SAEs) that occur on study therapy or within 30 days after the last dose of study therapy will be recorded on case report forms (CRFs).<br><br>Patients will be assessed for dose-limiting toxicities during Cycle 1 and 2, defined as any of the following AEs occurring during Cycle 1 or 2 of the dose-escalation period:<br><br>• Any non-hematologic treatment-related AE ≥ Grade 3, with the exceptions of Grade 3 nausea, vomiting, diarrhea, constipation, fever, fatigue, skin rash, or non-clinically significant laboratory abnormality that resolves to Grade ≤ 2 within 72 hours with optimal medical management.<br><br>• Grade 4 thrombocytopenia, or Grade 3 thrombocytopenia with Grade > 1 bleeding or requirement for platelet transfusion.<br><br>• Grade 4 neutropenia lasting for > 5 consecutive days<br><br>• Grade ≥ 3 febrile neutropenia.

Study Schema

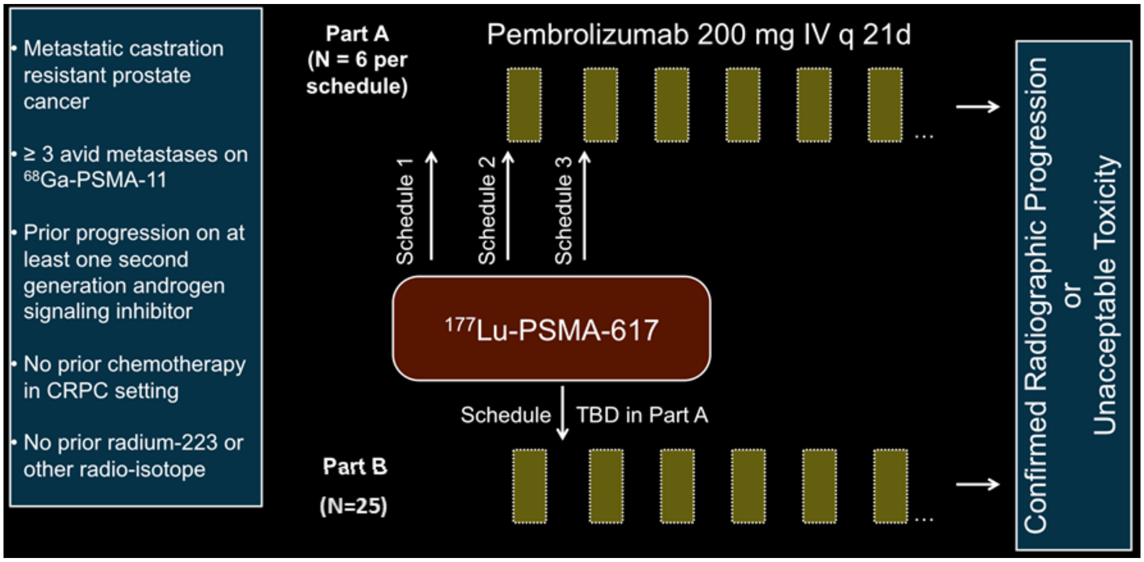

Table of Contents

Abstract 3

Study Schema 7

List of Abbreviations 10

1 Introduction 13

1.1 Background 13
1.2 PSMA and PSMA ligands 13
1.7 Study Rationale 16

2 Objectives and Endpoints of the Study 19

2.1 Objectives 19
Primary Objectives 19
Secondary Objectives 19
Correlative Objectives 19
2.2 Endpoints 20
2.2.1 Primary Endpoint 20
2.2.2 Secondary Endpoints 20

3 Study Design 20

3.1 Characteristics 20
3.2 Randomization 21
3.3 Eligibility Criteria 21
3.4 Duration of Study Treatment 25
3.5 Subject Replacement 25
3.6 Study Timeline 25
3.6.1 Primary Completion 25

4 Study Treatment 26

4.1 ^{177}Lu-PSMA-617 26
4.1.1 Chemical and physical characteristics 26
4.1.2 Drug preparation 27
4.1.3 Patient preparation 28
4.1.4 Concomitant hydration (renal protection) 28
4.1.5 Method of administration 28
4.1.6 Dose Limiting Toxicities and Criteria for Dose De-Escalation 29
4.1.7 Contraindications to ^{177}Lu-PSMA-617 29
4.1.8 Adverse reactions to ^{177}Lu-PSMA-617 29
4.1.9 Drug interactions 30
4.1.10 Warnings and precautions 30
4.1.11 Overdosage 31
4.1.12 Packaging and Labeling 31
4.1.13 Handling of Medication 31
4.2 Pembrolizumab 31
4.3 Packaging and Labeling Information 32
4.4 Clinical Supplies Disclosure 32
4.5 Storage and Handling Requirements 32
4.6 Returns and Reconciliation 32

4.7 Administration 32
4.8 Dose modification for Pembrolizumab 32
5 Study Procedures 42
5.1 Discontinuation of Therapy 43
5.2 Study Calendar: Schedule 1 (^{177}Lu-PSMA-617 prior to Pembrolizumab) 45
5.3 Study Calendar – Schedule 2 (Concurrent Dosing) 47
5.4 Study Calendar – Schedule 3 (^{177}Lu-PSMA-617 following Pembrolizumab) 49
6 Reporting and Documentation of Results 51
6.1 Evaluation of efficacy (or Activity) 51
6.1.1 Definitions 51
6.2 Evaluation of Safety 51
6.3 Definition of Adverse Events 51
6.3.1 Adverse Event 51
6.3.2 Adverse Reaction 51
6.4 Recording of an Adverse Event 54
6.5 Follow-up of Adverse Events 54
6.6 Expedited Reporting 54
7 Measurement of Response 57
8 Statistical Considerations 61
8.1 Sample Size Justification 61
8.2 Accrual 61
8.3 Interim Analyses 61
8.4 Analytic Plan 61
8.4.1 Analytic Plan for Primary Endpoints 61
8.4.2 Analytic Plan for Secondary Endpoints 62
9 Study Management 64
9.1 Pre-study Documentation 64
9.2 Institutional Review Board Approval 64
9.3 Informed Consent 64
9.4 Changes in the Protocol 65
10 Protection of Human Subjects 65
10.1 Protection from Unnecessary Harm 65
10.2 Protection of Privacy 65
References 66
Appendix 1 Performance Status Criteria 70
Appendix 2 Data and Safety Monitoring Plan for a Phase 1 Study 71
Appendix 3 UCSF Policy/Procedure for Required Regulatory Documents for a UCSF Investigator-Initiated Oncology Clinical Trials with an Investigator held Investigational New Drug (IND) 73
Appendix 4 Radioprotection Precautions for Patients Treated with ^{177}Lu-PSMA 75
Appendix 5 Recommended Precautions for Patients Treated with ^{177}Lu-PSMA 76
Appendix 6 National Cancer Institute Common Terminology Criteria for

Adverse Events..79

List of Abbreviations

5-HT3	Serotonin type-3
ADT	androgen deprivation therapy
AE	adverse event
ALT	alanine aminotransferase
ANC	absolute neutrophil count
AR	androgen receptor
AST	aspartate aminotransferase
BS	bone scan
BUN	blood urea nitrogen
CBC	complete blood cell (count)
CGMP	Current Good Manufacturing Practice
CMP	comprehensive metabolic panel
CR	complete response
CRC	Clinical Research Coordinator
CrCl	creatinine clearance
CRF	case report form
CT	computerized tomography
CTC	circulating tumor cell
CTCEA	Common Terminology Criteria for Adverse Events

List of Abbreviations

CTMS	Clinical Trial Management System
DKFZ	German Cancer Research Center of Heidelberg
DLT	Dose-limiting toxicity
DMT	dose-modifying toxicity
DOTA	1,4,7,10-tetraazacyclododecane-1,4,7,10-tetraacetic acid
DSMC	Data and Safety Monitoring Committee
DSMP	Data and Safety Monitoring Plan
DTPA	diethylene triamine pentaacetic acid
ECOG	Eastern Cooperative Oncology Group
FDA	Food and Drug Administration
Gbq	Gigabecquerel
Glu-urea	Glutamate-urea
F-18	Fluorine-18
^{68}Ga	Galium-68
GCP	Good Clinical Practice
Gy	Gray
HDFCCC	Helen Diller Family Comprehensive Cancer Center
HIPAA	Health Insurance Portability and Accountability Act (HIPAA)
ICF	informed consent form
ICH	International Conference on Harmonization
IND	investigational new drug application
INR	international normalized ratio
IRB	Institutional Review Board
IV	intravenous
LHRH	luteinizing hormone-releasing hormone
^{177}Lu	Lutetium-177
mCRPC	metastatic castration resistant prostate cancer
MR	magnetic resonance
MRI	magnetic resonance imaging
NCI	National Cancer Institute
PAP	prostatic acid phosphatase
PCWG3	Prostate Cancer Working Group 3

List of Abbreviations

PD-L1	Programmed death receptor ligand - 1
PET	positron emission tomography
PFS	progression free survival
PR	partial response
PRC	Protocol Review Committee
PSA	prostate specific antigen
PSMA	Prostate specific membrane antigen
RECIST	Response Evaluation Criteria in Solid Tumors
RLT	Radioligand therapy
SAE	serious adverse event
SUV	standardized uptake values
TCR	T cell receptor
ULN	upper limit of normal

1 Introduction

1.1 Background

Prostate cancer is the second leading cause of cancer death in men in the Western World (Siegel et al. 2015). While most men with localized prostate cancer are cured with treatment, men with recurrent or newly diagnosed metastatic disease have significant morbidity and mortality. In these patients, there is generally a rapid and favorable response to androgen deprivation therapy (ADT); however, in time, most men eventually develop hormone refractory disease, termed castration resistant prostate cancer (mCRPC).

Prior to 2010, docetaxel-based chemotherapy was the only treatment that demonstrated overall survival benefit in randomized trials for patients with mCRPC (Tannock et al. 2004, Petrylak et al. 2004, Berthold et al. 2008). In 2010, cabazitaxel, a novel taxane, gained FDA approval in mCRPC patients previously treated with docetaxel (Bahl et al. 2013). More recently, treatment options for mCRPC expanded to sipuleucel-T, an autologous cellular immunotherapy targeting PAP (Kantoff et al. 2010); abiraterone acetate and enzalutamide, novel androgen receptor (AR)-targeting agents which more completely ablate androgen signaling (de Bono et al. 2011, Fizazi et al. 2012, Ryan et al. 2013, Scher et al. 2012, Beer et al. 2014); and radium-223, a bone-targeting alpha emitter (Parker et al. 2013).

Despite these therapeutic advances, most men with mCRPC develop progressive disease, and many eventually succumb to their disease. Men with abiraterone and/or enzalutamide refractory mCRPC represents a particularly challenging subgroup who have limited effective treatment options outside of chemotherapy, which carries with it a significant risk of side effects. Therefore, there is a clinical need to develop novel therapeutics which is not only clinically efficacious, but also well tolerated in this often elderly, heavily pretreated patient population.

1.2 PSMA and PSMA ligands

Prostate-Specific Membrane Antigen (PSMA), also known as folate hydrolase I or glutamate carboxypeptidase II, is a cell surface protein that is expressed by normal prostate cells, and significantly overexpressed on most prostate cancer cells. Other normal tissues express low levels of PSMA, including the kidney, salivary glands, and small intestine (Afshar-Oromieh et al. 2013, Ahkhtar dt al 2012). In prostate cancer, PSMA expression has been correlated with disease grade and disease progression, with the highest expression levels seen in metastatic and castration resistant disease (Israeli et al. 1994, Wright et al. 1995, Wright et al. 1996).

Because PSMA is a type II transmembrane protein with glutamate-carboxypeptidase activity, efforts have been made to develop small-molecule ligands targeting its proteolytic domain for diagnostic and radiopharmaceutical purposes. ^{177}Lu-J591, a radiolabeled monoclonal antibody targeting the extracellular domain of PSMA, has shown moderate antitumor activity. However, the slow diffusion of antibodies and their long circulation time are associated with significant hematotoxicity (Tagawa et al. 2013). Small molecules—in contrast to antibodies—have relatively fast blood clearance resulting in a low background activity. Furthermore, after binding to PSMA, internalization of the small molecular ligand occurs via clathrin-coated pits and endocytosis (Rajasekaran et al. 2003). This leads to enhanced tumor uptake and retention, resulting in high image quality for diagnostic procedures and a high local dose for therapeutic applications.

1.3 RLT targeting PSMA is a promising treatment for mCRPC:

PSMA is also an optimal target for therapy because after binding to PSMA the ligand is internalized through clathrin-coated pits and endocytosis. The first attempt at PSMA-targeted RLT used an antibody (J591). Clinical results were limited with a PSMA fall of 50% seen in only 10% of patients while 47% of patients developed grade 4 thrombocytopenia. This initial stumble was subsequently followed by stunning results using small molecule urea-based agents. Using small molecules significantly reduced the associated marrow toxicity seen with antibodies due to the rapid clearance of blood pool activity, which allows for a marked improvement in the therapeutic window and therefore much higher doses can be safely administered. In 2015, a PSMA targeted radioligand (^{177}Lu-PSMA-617) was first reported as having the ability to target prostate cancer metastases for therapeutic intent. Like other theranostic agents, this compound can also be used to image metastatic lesions.

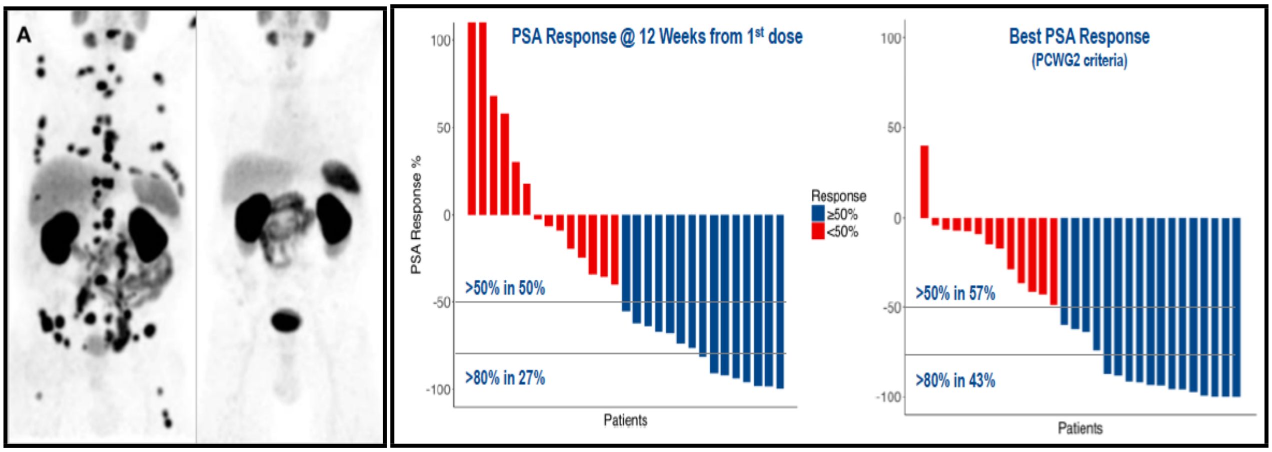

Subsequently numerous publications demonstrated efficacy of this treatment in CRPC. PSA declines of ≥ 50% are typically seen in >50% of heavily pretreated patients **(Figure above)** **(**Hofman et al. ESMO 2017**)**. Additionally near complete responses (radiographic and PSA) have been observed. It should be noted that ^{177}Lu-PSMA-617 is not the only urea-based small molecule that is in development for treating prostate cancer, but the approaches being studied in this application are broadly applicable to any RLT that targets PSMA. Importantly, tumor responses are rapid and frequently observed after just one dose of Lu-PSMA RLT.

Despite the significant anti-tumor activity with Lu-PSMA RLT, there are several significant limitations of this approach as monotherapy: 1) There is significant **heterogeneity of PSMA expression**, both in analysis of individual circulating tumor cells as well as diagnostic PSMA PET imaging of metastatic lesions across individual patients, and 2) Within PSMA-avid lesions, there is a **limited duration of response**. In a recently presented prospective phase 2 study of Lu-PSMA, despite enriching for patients with more homogenous level of PSMA uptake on baseline PET imaging, the median PSA progression-free survival by PCWG criteria was only 6.3 months (95% CI 4.8 – 8.3), and median overall survival only 12.7 months. Nearly half of the 30 patients enrolled received less than the pre-specified 4 doses of Lu-PSMA treatment, for both toxicity-related reasons (hematologic, salivary gland) as well as rapid disease progression following initial response. The reasons for rapid disease progression include baseline heterogeneity of PSMA expression among disseminated tumor cells, leading to diffuse bone marrow involvement in a subset of patients. There is a clear and unmet medical need to merge the rapid anti-tumor responses achieved by Lu-PSMA RLT with a treatment that can both sustain and spread response to PSMA-negative lesions without excess cumulative toxicity.

Immune checkpoint inhibition, by virtue of antigen spread induced by immunogenic cell death induced by antecedent radioligand treatment, may accomplish this goal.

1.4 Immune Checkpoint Inhibition Leads to Durable Responses in a Minority of mCRPC Patients

Single agent immune checkpoint inhibitor treatment, including therapies blocking PD-L1/PD-1 as well as CTLA-4 mediated signaling, have demonstrated that durable anti-tumor responses can be achieved in mCRPC. Immune checkpoint inhibitors including ipilimumab and pembrolizumab have led to durable tumor responses in a limited number of cases in mCRPC, but increasing the proportion of patients who respond to single agent checkpoint blockade has been challenging. Dr. Fong at UCSF led the first in human study of ipilimumab in mCRPC. Overall, the tumor immunogenicity, as reflected by mutational burden, tumor infiltrating lymphocytes, and tumor PD-L1 expression, are low in prostate tumors relative to other tumor types such as melanoma. In the KEYNOTE-028 study of pembrolizumab in mCRPC patients, the tissue screen positivity rate for PD-L1 expression in archival tumor tissue was less than 10%, and even in PD-L1 positive tumors, the objective response rate by RECIST 1.1 criteria was only 17%. However, in those patients who experienced a tumor response, there was durable regression of disease with median duration of response of over 15 months. For the majority of mCRPC patients who do not benefit from single agent immune checkpoint inhibitors, there is an unmet medical need to enhance tumor immunogenicity with priming therapy to enhance TILs, induce PD-L1 expression, and expand the T cell repertoire, thereby translating into a significant improvement in proportion of patients achieving durable tumor response.

1.5 Immunogenic Priming Effect of Radiation Treatment

Radiation treatment has long been recognized as a means to induce an anti-tumor response by the host immune system. The so-called ‘abscopal effect’, well described in prostate and other malignancies, is reflective of the ability of radiation treatment to induce antigen spread, thereby stimulating the host immune system to generate an anti-tumor response against distant sites of disease. More contemporary data provided a mechanistic link between radiation and immunogenic priming. Radiotherapy has the potential to enhance anti-tumor effects of immune checkpoint blockade by multiple mechanisms, including providing antigen presentation, releasing proinflammatory signals from dying tumor cells, and enhancing the diversity of TCR repertoire of intratumoral T cells. Preclinical mouse models have shown that the combination of RT with immunotherapies can enhance anti-tumor efficacy and improve long-term survival, including in glioma, melanoma and breast cancer.

Collectively, these data provide a strong link between radiotherapy-mediated induction of host anti-tumor immune response. The logical extension of these findings is that with greater the magnitude of immunogenic cell death, the greater the host immune response against the tumor, with the potential for spreading of host immune response. PSMA-targeted radiotherapy, therefore, is predicted to have a significantly higher immunogenic priming effect than focal radiation to a solitary metastasis, as well as greater effect than bone-only radioligands such as radium-223, which do not lead to cytoreduction in soft tissue metastases (node and/or visceral). These hypotheses will be clinically investigated in our proposed proof-of-concept phase 1b study.

1.6 Measures of Immunogenicity Predictive of Outcomes with Immune Checkpoint Inhibitors

Dr. Lawrence Fong (Co-Investigator) has pioneered methods to quantify the immunogenicity of systemic anti-cancer therapies and develop biomarkers predictive of long-term response to immune checkpoint inhibitors in mCRPC and other tumor types. Dr. Fong has pioneered efforts to develop biomarkers to predict for responsiveness to checkpoint blockade with ipilimumab. Use of these biomarkers prior to and early in the course of checkpoint inhibitor treatment may serve as a key biomarker to predict the immunogenicity of novel checkpoint inhibitor combinations applied in the mCRPC setting. The Fong laboratory used next-generation sequencing to measure the frequency of individual rearranged T cell receptor ß (TCRß) genes in mCRPC patients, characterizing their clonotypic diversity upon treatment with the anti-CTLA-4 antibody ipilimumab. Dr. Fong demonstrated that ipilimumab treatment increased TCR diversity and the repertoire of clonotypes continued to evolve over subsequent months of treatment **(Figure 4, above)**. Notably, improved overall survival was associated with maintenance of high-frequency clones at baseline, as opposed to expansion of clonotypic diversity, suggesting that pre-existing activated T cells are important in achieving prolonged response and improved survival with immune checkpoint blockade[46]. The logical extension of these findings, therefore, is to develop novel therapies that first prime the immune system, via generation of activated T cells with high TCR avidity, which may increase the proportion of durable response in mCRPC with the subsequent application of immune checkpoint inhibition.

1.7 Study Rationale

Despite encouraging data of ^{177}Lu-PSMA in men with advanced prostate cancer, the responses are limited in duration. Rather than attempt to continue men on treatment that may become ineffective or lead to excessive toxicity, we will utilize Lu-PSMA RLT in a novel way, via immunologic priming with subsequent pembrolizumab to maintain response in a durable fashion. In this Phase 1b dose escalation/expansion study we will garner the necessary safety and preliminary efficacy data to enable a decision whether to proceed to phase 2/3 study with this treatment combination.

1.7.1 Rationale for the Starting Dose and Proposed Dose Schedules of ^{177}Lu-PSMA-617 in Combination with Pembrolizumab

The proposed starting dose of ^{177}Lu-PSMA-617 will be 7.4 Gbq (+/- 10%), which is the recommended phase 2/3 dose of the compound as monotherapy. In the aforementioned prospective single arm phase 2 study of ^{177}Lu-PSMA-617 in 30 patients with mCRPC, the dose utilized was 8 Gbq (+/- 10%), which yielded acceptable safety and dosimetry results as outlined below:

Non haematological attributable to LuPSMA:

Toxicity	G1/2 (%)	G3/4 (%)
Dry mouth	63	0
Nausea*	50	0
Vomiting*	20	0
Fatigue	17	3
Dry eyes	7	0
Bone pain	7	3
Anorexia	7	0
Infusion related reactions	0	0
Renal toxicity	0	0

Haematotoxicity:

Toxicity	G1/2 (%) (baseline)	G1/2 (%) (any cause)	G3/4 (%) (any cause)	G3/4 (%) (LuPSMA)
Haemoglobin	80	73	23	7
Neutrophils**	0	40	10	7
Platelets	17	43	27	13

* transient and self-limiting within first 24 hours
** no episodes of febrile neutropenia

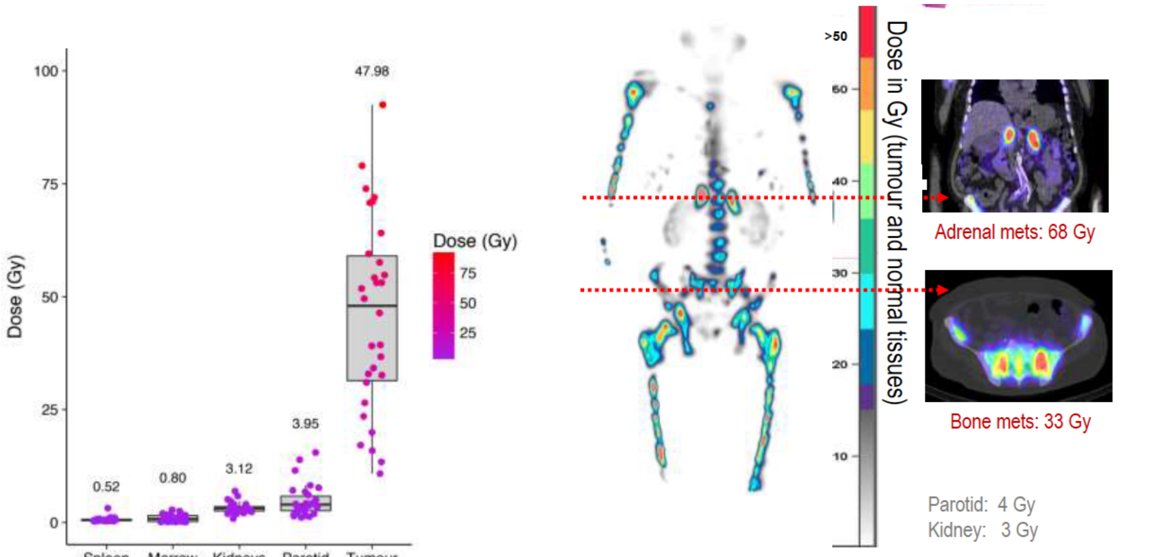

Voxel-based Monte-Carlo dosimetry from 4, 24 & 96hr qSPECT/CT (8GBq ^{177}Lu-PSMA617)

For the current study, we will investigate a single planned priming dose of ^{177}Lu-PSMA-617 at a starting dose of 7.4 GBq (+/- 10%), in combination with the FDA-approved dose and schedule of pembrolizumab of 200 mg IV every 21 days. Given the non-overlapping toxicities between these two agents, the safety profile of repeated ^{177}Lu-PSMA-617 dosing demonstrated in the prior phase 2 study, and the fact that we are planning a single dose of ^{177}Lu-PSMA-617, this is an acceptable starting dose level.

If the DLT frequency exceeds 33% during Cycles 1 and 2 for the first 6 patients enrolled on Part A of the study, a lower dose level (Dose Level -1) of ^{177}Lu-PSMA-617 (4 GBq +/- 10%) will be investigated for subsequent patients enrolled in Part A.

With respect to dose schedule, the majority of trials combining radiation-based treatment with immune checkpoint inhibition have utilized schedules in which the priming dose of radiation is given prior to or concurrently with initiation of immune checkpoint inhibition. However the optimal timing of radiation priming is not well established in prospective clinical trials, and rationale arguments support giving priming radiation before or after the first dose of immune checkpoint inhibition. In the current study, three schedules will be explored in Part A, with priming ^{177}Lu-PSMA-617 delivered prior to, concurrently, or following the first dose of pembrolizumab, respectively. The aggregate safety, efficacy, and immunologic parameters observed in Part A will be utilized to determine the optimal dose/schedule of ^{177}Lu-PSMA-617 in conjunction with pembrolizumab to be further evaluated in Part B of the study.

As of June 1, 2020, 14 patients have been enrolled in Part A of the study. There has been one dose-limiting toxicity (Grade 3 inflammatory arthritis in Schedule 2) observed to date. There have not been any other Grade ≥ 3 immune related adverse events outside of this DLT, nor any Grade ≥ 3 hematologic toxicities attributable to the priming dose of ^{177}Lu-PSMA-617. Encouraging preliminary evidence of efficacy has been observed with objective response rate by RECIST 1.1 criteria of 3/6 patients (50%), and the median duration of response has not been reached. Below shows a PSA plot of the two of the patients with ongoing objective + PSA90 responses to treatment.

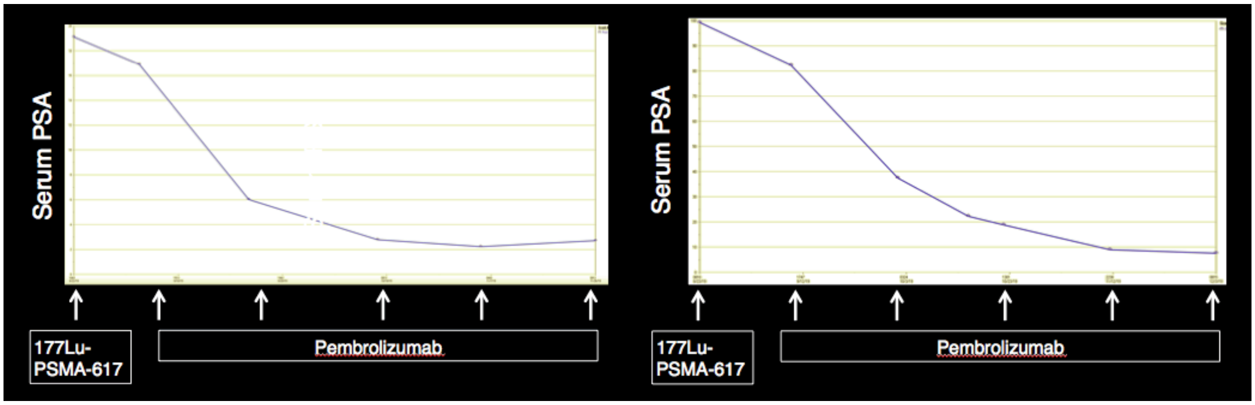

Given the encouraging preliminary efficacy and safety data, as well as to obtain additional correlative samples including paired tumor biopsies that may help to further identify patients more likely to respond to treatment, we will increase the pre-specified Dose Expansion sample size from 12 to 25 patients.

2 Objectives and Endpoints of the Study

2.1 Objectives

Primary Objectives

Part A: To determine the recommended phase 2 dose and schedule of ^{177}Lu-PSMA-617 in combination with pembrolizumab in patients with mCRPC.

Part B (Dose Expansion): To determine the objective response rate by RECIST 1.1 criteria.

Secondary Objectives

1. To characterize the safety profile of the combination.
2. To determine the median duration of response by RECIST 1.1 criteria.
3. To determine the proportion of patients who experience ≥ 50% decline from baseline in serum PSA.
4. To determine the median PSA progression-free survival.
5. To determine the median time to symptomatic skeletal related event.
6. To determine the 6 month radiographic progression-free survival rate and median radiographic progression-free survival.
7. To determine the median overall survival.

Correlative Objectives

1. To assess the lesion-specific response rate by baseline PSMA avidity on ^{68}Ga-PSMA-11 PET.
2. To quantify the change from baseline in T cell repertoire, circulating T cell subsets, tumor infiltrating lymphocytes, and tumor PD-L1 expression by immunohistochemistry after one priming dose of Lu-PSMA RLT.
3. To explore the relationship between timing of the ^{177}Lu-PSMA-617 priming dose with initiation of pembrolizumab with respect to immunologic, safety, and efficacy outcomes.
4. To descriptively characterize the patterns of uptake on ^{68}Ga-PSMA-11 PET at the time of disease progression.
5. To explore relationship between tumor genomic profile with clinical outcomes including response rate and progression-free survival.
6. To explore the relationship between tumor dosimetry with objective response.

2.2 Endpoints

2.2.1 Primary Endpoint

Part A: Safety profile of ^{177}Lu-PSMA-617 in combination with pembrolizumab as graded by CTCAE criteria.

Part B: Objective response rate by RECIST 1.1 criteria

2.2.2 Secondary Endpoints

1. Frequency and severity of adverse events as defined by CTCAE criteria.
2. Median duration of response by RECIST 1.1
3. PSA response rate
4. Radiographic progression-free survival rate at 6 months and median radiographic progression-free survival.
5. Overall survival

3 Study Design

3.1 Characteristics

This is a single-center Phase 1b open-label, study of PSMA-targeted radionuclide therapy with ^{177}Lu-PSMA-617 in combination with pembrolizumab in patients with mCRPC who have previously progressed on at least one prior androgen pathway inhibitor (e.g. abiraterone, enzalutamide, apalutamide). Patients must have at least three metastatic lesions which are PSMA-avid on baseline ^{68}Ga-PSMA-11 PET imaging.

In **Part A** of the study, patients will be enrolled in sequential fashion to one of treatment schedules with respect to timing of ^{177}Lu-PSMA-617 in relation to first dose of pembrolizumab (N = 6 evaluable patients per schedule):

Schedule 1) Priming dose of ^{177}Lu-PSMA-617 administered on Cycle 1 Day 1 followed by pembrolizumab 200 mg IV q 21 days starting Cycle 2 Day 1.

Schedule 2) Priming dose of ^{177}Lu-PSMA-617 administered concurrently with initiation of pembrolizumab on Cycle 1 Day 1.

Schedule 3) Priming dose of ^{177}Lu-PSMA-617 administered on Cycle 1 Day 1 following initiation of pembrolizumab on Cycle 1 Day -21.

The priming dose of ^{177}Lu-PSMA-617 will be 7.4 +/- 15% GBq, which is the recommended phase 3 dose of the agent. If the DLT frequency exceeds 33% including patients across the three schedules, Dose Level -1 of ^{177}Lu-PSMA-617 will be investigated (4 Gbq +/- 15%).

6 evaluable patients will be enrolled in each of the three cohorts in sequential fashion in Part A of the study. The DLT window will be Cycle 1 + Cycle 2. If the DLT frequency is ≥ 33% for a

particular cohort, that dosing schedule will be determined to be non-tolerable and the next schedule will be evaluated. In addition, if the DLT frequency attributable specifically to Lu-PSMA-617 is ≥ 33% after 6 or more evaluable patients are enrolled in Part A of the study, the dose of Lu-PSMA-617 will be reduced to Dose Level -1 (4 GBq +/- 15%) for the remainder of patients enrolled on study. If the DLT frequency attributable to Lu-PSMA-617 at Dose Level -1 is ≥ 33% after 6 or more patients are treated at Dose Level -1, study accrual will be halted.

Using the data obtained in Part A, the recommended phase 2 dose and schedule of ^{177}Lu-PSMA-617 will be determined by the DSMC and Principal Investigator using a combination of safety, efficacy, and immunologic effects of treatment from the first two cycles (and additional cycles of treatment when available).

In **Part B** of the study, an additional 25 patients will be enrolled at the recommended phase 2 dose and schedule to obtain additional safety, efficacy, and immunologic data pertaining to the treatment combination.

Number of Subjects

Approximately 43 patients will be enrolled over an accrual period of approximately 18 months (N ~ 18 patients in Part A; 25 patients in Part B).

3.2 Randomization

There is no randomization for the study.

During Part A of the study, patients will be enrolled in sequential fashion to one of three dosing schedules of ^{177}Lu-PSMA-617 as outlined above. 6 patients will be enrolled to Schedule 1, followed by 6 patients enrolled on Schedule 2, and then 6 patients will be enrolled on Schedule 3.

During Part B of the study, all patients enrolled will receive the recommended phase 2 dose and schedule of ^{177}Lu-PSMA-617 plus pembrolizumab.

3.3 Eligibility Criteria

Patients must have baseline evaluations performed prior to Cycle 1 Day 1 of treatment and must meet all inclusion and none of the exclusion criteria. In addition, the patient must be thoroughly informed about all aspects of the study, including the study visit schedule and required evaluations and all regulatory requirements for informed consent. The written informed consent must be obtained from the patient prior to enrollment. The following criteria apply to all patients enrolled onto the study unless otherwise specified.

Inclusion Criteria

Part A:

(1) The subject is able and willing to comply with study procedures and provide signed and dated informed consent.

(2) Male participants who are at least 18 years of age on the day of signing informed consent.

(3) Histologically confirmed prostate adenocarcinoma. *De novo* small cell neuroendocrine prostate cancer will not be allowed due to putative lower PSMA expression in this tumor subtype. Treatment-emergent small cell neuroendocrine prostate cancer detected in metastatic tumor biopsy is not an exclusion.

(4) A minimum of three PSMA-avid lesions on baseline ^{68}Ga-PSMA-11 PET, with positive lesions defined as those with SUVmax values greater than liver.

(5) Progressive metastatic castration-resistant prostate cancer by PCWG3 criteria at the time of study entry.

(6) Castrate level of serum testosterone at study entry (< 50 ng/dL). Patients without prior bilateral orchiectomy are required to remain on LHRH analogue treatment for duration of study

(7) Prior progression on at least one second generation androgen signaling inhibitor including abiraterone, apalutamide, darolutamide, and/or enzalutamide.

(8) Adequate hematologic, renal, and liver function, defined as:

- Absolute neutrophil count > 1.5 x 10^9/L
- Hemoglobin > 9.0 g/dL
- Platelet count > 100,000/microliter
- Serum creatinine ≤ 1.5 x ULN or estimated GFR > 50 ml/min by Cockroft-Gault or 24 hour urine collection.
- Total bilirubin ≤ 1.5 x ULN. In patients with known or suspected Gilbert's disease, direct bilirubin ≤ ULN.
- Aspartate aminotransferase and alanine aminotransferase ≤ 2.5 x ULN (≤ 5 x ULN in patients with liver metastases).

(9) No other systemic anti-cancer therapies administered other than LHRH analogue within 14 days, or 5 half-lives, whichever is shorter, prior to initiation of study treatment. Adverse events related to prior anti-cancer treatment other than LHRH analog treatment must have recovered to Grade ≤ 1 with the exception of any grade alopecia and grade ≤ 2 neuropathy.

(10) Patients must have an ECOG performance status of 0 or 1.

(11) Patients must use appropriate methods of contraception during study treatment and for at least 60 days after last study treatment.

- Patients who are sexually active should consider their female partner to be of childbearing potential if she has experienced menarche and is not postmenopausal (defined as amenorrhea > 24 consecutive months) or has not undergone successful surgical sterilization. Even women who

use contraceptive hormones (oral, implanted, or injected), an intrauterine device, or barrier methods (diaphragms, condoms, spermicide) should be considered to be of childbearing potential.

- Patients who have undergone vasectomy themselves should also be considered to be of childbearing potential.
- Acceptable methods of contraception include continuous total abstinence, or double-barrier method of birth control (e.g. condoms used with spermicide, or condoms used with oral contraceptives). Periodic abstinence and withdrawal are not acceptable methods of contraception.

(12) Patients must provide consent to comply to recommended radioprotection precautions during study

(13) Patients willing to undergo tumor biopsy and have at least one lesion safely accessible to tumor biopsy. Bone or soft tissue lesion is allowed.

(14) Measurable disease by RECIST 1.1 criteria

Exclusion Criteria

(1) Untreated brain metastases at study entry. Patients with previously treated brain metastases are eligible provided the following criteria are all met:

- Last treatment was > 28 days prior to C1D1
- No evidence of new/progressive brain metastases is observed on MRI obtained during Screening window
- Patient is clinically stable without requirement of steroid treatment for at least 14 days prior to first dose of study treatment.

(2) Receipt of prior PSMA-directed treatment (e.g. radiotherapy, immunotherapy, or antibody-drug conjugate).

(3) Prior enrollment on clinical study investigating Lu-PSMA-based radioligand therapy

(4) Prior treatment with radium-223 or other radioisotope for the treatment of prostate cancer.

(5) Has received prior radiotherapy within 2 weeks of start of study treatment. Participants must have recovered from all radiation-related toxicities, not require corticosteroids, and not have had radiation pneumonitis. A 1-week washout is permitted for palliative radiation (≤2 weeks of radiotherapy) to non-CNS disease.

(6) Receipt of prior pembrolizumab or another immune checkpoint inhibitor (e.g. nivolumab, ipilimumab).

(7) Is currently participating in or has participated in a study of an investigational agent or has used an investigational device within 4 weeks prior to the first dose of study treatment.

Note: Participants who have entered the follow-up phase of an investigational study may participate as long as it has been 4 weeks after the last dose of the previous investigational agent.

(8) Receipt of taxane chemotherapy applied in the castration-resistant setting. Prior receipt of taxane chemotherapy in the hormone-sensitive setting is allowed.

(9) Grade > 2 peripheral neuropathy at the time of study entry

(10) Has severe hypersensitivity (≥Grade 3) to pembrolizumab and/or any of its excipients.

(11) Has an active autoimmune disease that has required systemic treatment in the past 2 years (i.e., with use of disease modifying agents, corticosteroids or immunosuppressive drugs). Replacement therapy (e.g., thyroxine, insulin, or physiologic corticosteroid replacement therapy for adrenal or pituitary insufficiency) or treatment with drugs (e.g. neomercazol, carbamazole, etc.) that function to decrease the generation of thyroid hormone by a hyperfunctioning thyroid gland (e.g in Graves' disease) is not considered a form of systemic treatment of an autoimmune disease.

(12) Has a diagnosis of immunodeficiency or is receiving systemic steroid therapy at a prednisone equivalent dose of > 10 mg daily or other form of immunosuppressive therapy within 7 days prior to first dose of study drug.

(13) Has a history of (non-infectious) ≥ grade 2 pneumonitis/interstitial lung disease that required steroids within past 2 years or has current ≥ grade 1 pneumonitis/ interstitial lung disease at the time of study enrollment.

(14) Has received a live vaccine or live-attenuated vaccine within 30 days prior to the first dose of study drug. Administration of killed is allowed.

(15) Patients who because of age, general medical or psychiatric condition, or physiologic status cannot give valid informed consent.

(16) Has clinically significant cardiovascular disease including, but not limited to:

- Uncontrolled or any New York Heart Association Class 3 or 4 congestive heart failure.
- Uncontrolled angina, history of myocardial infarction, unstable angina or stroke within 6 months before study entry.
- Clinically significant arrhythmias not controlled by medication. Chronic rate controlled or paroxysmal atrial fibrillation/flutter is not an exclusion to study participation.

(17) Prior external beam radiation involving ≥ 25% of bone marrow or within 14 days of start of protocol therapy.

(18) Major surgery within 28 days of study treatment. Note: If participant received major surgery, they must have recovered adequately from the toxicity and/or complications from the intervention prior to starting study treatment.

(19) Has an active infection requiring systemic therapy

(20) Has a known history of HIV (screening not required).

(21) Has a known history of Hepatitis B (defined as Hepatitis B surface antigen [HBsAg] reactive) or known active Hepatitis C virus (defined as HCV RNA [qualitative] is detected) infection (screening not required)

(22) Has a known history of active TB (Bacillus Tuberculosis).

(23) Has known psychiatric or substance abuse disorders that would interfere with cooperation with the requirements of the trial.

(24) Any condition that, in the opinion of the Principal Investigator, would impair the patient's ability to comply with study procedures

(25) History of bleeding diathesis and not currently on anti-coagulation therapy that cannot be safely discontinued for the tumor biopsy procedure.

3.4 Duration of Study Treatment

Patients will be treated until disease progression per PCWG3/RECIST 1.1 criteria, confirmed by repeat scans performed ≥ 4 weeks later, unacceptable toxicity, or patient withdrawal, whichever occurs first.

Patients with PSA-only progression in absence of radiographic or unequivocal clinical progression should remain on study treatment.

3.5 Subject Replacement

All subjects who receive the priming dose of ^{177}Lu-PSMA treatment will be analyzed for study outcomes.

Subjects who withdraw from study participation prior to receiving dose of ^{177}Lu-PSMA-617 will be not be evaluable and will be replaced.

3.6 Study Timeline

3.6.1 Primary Completion

The study is anticipated to accrue 2-3 patients per month for an estimated accrual period of 21 months. The study is expected to reach completion for analysis of primary and secondary endpoints approximately 36 months from first patient enrolled.

4 Study Treatment

Treatment regimen

^{177}Lu-labeled PSMA is a PSMA-targeted radioligand that will be produced under Current Good Manufacturing Practice (CGMP) regulations.

In **Part A** of the study, patients will be enrolled in sequential fashion to one of three treatment schedules with respect to timing of ^{177}Lu-PSMA-617 in relation to first dose of pembrolizumab (6 evaluable patients per schedule):

Schedule 1) Priming dose of ^{177}Lu-PSMA-617 administered on Cycle 1 Day 1 followed by pembrolizumab on Cycle 2 Day 1.

Schedule 2) Priming dose of ^{177}Lu-PSMA-617 administered concurrently with initiation of pembrolizumab on Cycle 1 Day 1.

Schedule 3) Priming dose of ^{177}Lu-PSMA-617 administered on Cycle 1 Day 1 following initiation of pembrolizumab on Cycle 1 Day -21.

Pembrolizumab will be given at the FDA approved dose of 200 mg via IV every 3 weeks.

Dose-limiting toxicity data, additional safety data beyond Cycle 2 where available, along with efficacy and pharmacodynamic data, will be utilized to determine the recommended phase 2 dose and schedule of ^{177}Lu-PSMA-617 in combination with pembrolizumab to be utilized in Part B of the study.

4.1 ^{177}Lu-PSMA-617

4.1.1 Chemical and physical characteristics

^{177}Lu-PSMA-617 contains a radionuclide (^{177}Lu) chelated to PSMA, a PSMA ligand consisting of a Glu-urea motif and DOTA chelator connected by a lipophilic linker.

Lutetium (^{177}Lu) decays to stable hafnium (^{177}Hf) with a half-life of 6.7 days, by emitting β_- radiation with a maximum energy of 0.498 MeV and photonic radiations (γ) of 0.208 MeV (11.0%) and 0.113 MeV (6.4%). The main radiations are detailed in **Table 1**, and physical decay summarized in **Table 2**.

Radiation	Energy (keV)	Iβ%	Iγ%
β-	176.5	12.2	-
β-	248.1	0.05	-
β-	384.9	9.1	-
β-	497.8	78.6	-
γ	71.6	-	0.15

γ	112.9	-	6.40
γ	136.7	-	0.05
γ	208.4	-	11.0
γ	249.7	-	0.21
γ	321.3	-	0.22

Table 1. ^{177}Lu main radiation.

Hours	Fraction Remaining	Hours	Fraction Remaining
0	1.000	48 (2 days)	0.813
1	0.996	72 (3 days)	0.733
2	0.991	168 (7 days)	0.485
5	0.979	336 (14 days)	0.235
10	0.958	720 (30 days)	0.045
24 (1 day)	0.902	1080 (45 days)	0.010

Table 2. Physical decay chart for ^{177}Lu.

4.1.2 Drug preparation

Administration procedures should be carried out in a way to minimize risk of contamination from the medicinal product and accidental irradiation of the operators. It is necessary to wear waterproof gloves and suitable effective protection when handling ^{177}Lu-PSMA-617.

Flow valves should be manipulated aseptically when connecting them to the infusion system.

Any unused medicinal product or waste material should be disposed of in accordance with local and federal laws.

The solution should be inspected visually prior to use, and only clear colorless to yellowish solutions free of visible particles should be used. The visual inspection of the solution should be performed under a shielded screen for radioprotection purposes.

The amount of radioactivity in the radiopharmaceutical vial must be measured with an appropriate and calibrated device prior to infusion in order to confirm that the actual amount of radioactivity to be administered is equal to the planned amount at the infusion time.

4.1.3 Patient preparation

Patient should be encouraged to drink a sufficient amount of water necessary to urinate every hour on the day of infusion and the day after. Patient should be encouraged to defecate every day and to use laxative if needed.

The doctor will explain to the patient the general recommendations and precautions that should be followed to limit radiation exposure to third parties.

To avoid treatment-related nausea and vomiting, it is recommended to inject an intravenous bolus of 5-HT3 receptor antagonist (e.g. ondansetron 8mg) 30 minutes before the start of ^{177}Lu-PSMA infusion.

4.1.4 Concomitant hydration (renal protection)

Concomitant infusion of intravenous fluids is required for renal protection. This infusion should start 30 minutes before the start of ^{177}Lu-PSMA-617. 500 – 1000 mL of normal saline should be administered between 250-500 mLs per hour. During the administration of IV fluids, the patient should be encouraged to urinate as frequently as possible.

4.1.5 Method of administration

^{177}Lu-PSMA is a ready to use single dose vial.

^{177}Lu-PSMA must be administered by slow intravenous infusion, over a 20 to 30-minute time span (see Appendix 4 for administration method examples).

^{177}Lu-PSMA must not be injected as a bolus.

The following table summarizes the required procedure.

Table 5. ^{177}Lu-PSMA administration procedure

**Product to inject**	**Start time (hr)**<br>**(+/- 30 min)**	**Infusion rate* (mL/h)**	**Duration**
5-HT3 receptor antagonist (e.g. ondansetron 8mg)**	0	Bolus	-
Hydration (normal saline)	0	250—550	-
^{177}Lu-PSMA	0.5	-	20-30 minutes

* Infusion rates may be reduced at the discretion of the physician.
** Additional prophylactic antiemetic regimen may be given at the discretion of the treating physician as clinically indicated (e.g. Prochlorperazine, Aprepitant, Lorazepam).

After the administration is completed, the infusion line and ^{177}Lu-PSMA vial must be immediately flushed with at least 25 mL of sodium chloride 9 mg/mL solution for injection, unless the infusion method used already requires NaCl to flow through the vial and the line.

4.1.6 Dose Limiting Toxicities and Criteria for Dose De-Escalation

The starting dose level of ^{177}Lu-PSMA-617 is 7.4 GBq (+/- 15%).

Patients will be assessed for dose-limiting toxicities during Cycle 1 and 2 for patients enrolled in Part A of the study, defined as any of the following AEs occurring during Cycle 1 or 2:

- Any non-hematologic treatment-related AE ≥ Grade 3, with the exceptions of Grade 3 nausea, vomiting, diarrhea, constipation, fever, fatigue, skin rash, or non-clinically significant laboratory abnormality that resolves to Grade ≤ 2 within 72 hours with optimal medical management.
- Grade 4 thrombocytopenia, or Grade 3 thrombocytopenia with Grade > 1 bleeding or requirement for platelet transfusion.
- Grade 4 neutropenia lasting for > 5 consecutive days
- Grade ≥ 3 febrile neutropenia.

6 evaluable patients will be enrolled in each of the three cohorts in sequential fashion in Part A of the study. If the DLT frequency is ≥ 33% for a particular cohort, that dosing schedule will be determined to be non-tolerable and the next schedule will be evaluated. In addition, if the DLT frequency attributable specifically to Lu-PSMA-617 is ≥ 33% after 6 or more evaluable patients are enrolled in Part A of the study, accrual will be temporarily halted and the dose of Lu-PSMA-617 will be reduced to Dose Level -1 (4 GBq +/- 15%) for the remainder of patients enrolled on study. If the DLT frequency attributable to Lu-PSMA-617 at Dose Level -1 is ≥ 33% after 6 or more patients are treated at Dose Level -1, study accrual will be halted.

Patients who discontinue study for reasons other than treatment related toxicity during prior to the completion of two cycles of therapy, or those who discontinue study prior to receiving priming dose of ^{177}Lu-PSMA-617, will be considered inevaluable for purposes of DLT determination.

4.1.7 Contraindications to ^{177}Lu-PSMA-617

Hypersensitivity to the active substance or to any of the excipients.

4.1.8 Adverse reactions to ^{177}Lu-PSMA-617

- Nausea
- Fatigue
- Xerostomia
- Hypogeusia
- Anorexia
- Bone pain

- Dry eyes
- Thrombocytopenia
- Neutropenia
- Anemia

4.1.9 Drug interactions

There are no known drug interactions with ^{177}Lu-PSMA-617.

4.1.10 Warnings and precautions

General warnings and precautions
Given the tolerance profile of ^{177}Lu-PSMA, it is not recommended to start a treatment in the following cases:

- Previous external beam radiotherapy involving more than 25% of the bone marrow
- Renal failure with serum creatinine > 1.5 mg/dL or estimated creatinine clearance < 50 mL/min
- Impaired hematological function with either Hb < 9 g/dL, platelets < 100 x 10^9/L, or ANC < 1.5 x 10^9/L

Radiation exposure
See **Appendix 4 and 5** for full details regarding protection from radiation exposure.

It is recommended that the patient urinate as much as possible during this period. Urine and feces should be disposed according to the regulation. The patient can leave the controlled area or the hospital only when radiation exposure to third parties does not exceed regulatory thresholds.

As long as there is no accidental contamination, such as from the leakage of the infusion system or because of urinary incontinence, radioactivity contamination is not expected on the patient's skin. However, it is recommended that when conducting standard care or exams with medical devices or other instruments which contact the skin (e.g. ECG), basic protection measures be observed such as wearing gloves, installing the material/electrode before the start of radiopharmaceutical infusion, changing the material/electrode after measurement, and eventually monitoring the radioactivity of equipment after use.

At the time of release, patients are given written instructions (**Appendix 5**) which outline the precautions the patient must take to minimize radiation exposure to people around them.

Men of childbearing potential
Ionizing radiations of ^{177}Lu-PSMA-617 may potentially have temporary toxic effects on male gonads. Due to the presence of the radionuclide in the product, men of childbearing potential must refrain from childbearing by using effective contraception methods during treatment with ^{177}Lu-PSMA and for 2 months after last dose.

Fertility

Fertility can be temporarily affected in men due to radiation damage to Sertoli cells, as evidenced by a decrease of inhibin-B and a concomitant increase of FSH (Teunissen et al., 2009). Fertility is usually restored 12 to 18 months after treatment. Cryopreservation of sperm might be offered to male patients before treatment.

4.1.11 Overdosage

Overdose is unlikely with ^{177}Lu-PSMA-617 as this medicinal product is supplied as a “single dose” and “ready to use” product containing a predefined amount of radioactivity.

In the event of administration of a radiation overdose with ^{177}Lu-PSMA:

- If not clinically contraindicated, hydration should be reinforced during the first 48 hours after infusion. Frequent bladder voiding must also be suggested to the patient. This will contribute to reducing the radiation dose to the patient by increasing the elimination of the radionuclide from the body
- If possible, an estimate of the radiation dose given to the patient should be performed
- Forced diuresis might also be considered
- For the next 10 weeks, the following should be carried out every week:
    - Hematologic monitoring: white blood cells, platelets, and hemoglobin
    - Blood chemistry monitoring: serum creatinine and liver functions.

4.1.12 Packaging and Labeling

^{177}Lu-PSMA-617 will be prepared, packaged, labeled, and released under Good Manufacturing Practice (GMP) guidelines, ICH Good Clinical Practice (GCP) guidelines, and applicable local laws/regulations.

4.1.13 Handling of Medication

^{177}Lu-PSMA-617 must be administered at the investigational site.

The medication must be handled and administered only by qualified/authorized personnel and must be prepared in accordance with pharmaceutical quality requirements, and radiation safety regulations for ^{177}Lu-PSMA-617.

4.2 Pembrolizumab

The investigator shall take responsibility for and shall take all steps to maintain appropriate records and ensure appropriate supply, storage, handling, distribution and usage of investigational product in accordance with the protocol and any applicable laws and regulations.

Pembrolizumab will be provided by Merck as summarized in the Table below:

Product Descriptions

Product Name & Potency	Dosage Form
Pembrolizumab 100 mg/ 4mL	Solution for Injection

4.3 Packaging and Labeling Information

Supplies will be labeled in accordance with regulatory requirements.

4.4 Clinical Supplies Disclosure

This trial is open-label; therefore, the participant, the trial site personnel, the Sponsor and/or designee are not blinded to treatment. Drug identity (name, strength) is included in the label text; random code/disclosure envelopes or lists are not provided.

4.5 Storage and Handling Requirements

Clinical supplies must be stored in a secure, limited-access location under the storage conditions specified on the label.

Receipt and dispensing of trial medication must be recorded by an authorized person at the trial site.

Clinical supplies may not be used for any purpose other than that stated in the protocol.

4.6 Returns and Reconciliation

The investigator is responsible for keeping accurate records of the clinical supplies received from Merck or designee, the amount dispensed to and returned by the participants and the amount remaining at the conclusion of the trial.

Upon completion or termination of the study, all unused and/or partially used investigational product will be destroyed at the site per institutional policy. It is the Investigator's responsibility to arrange for disposal of all empty containers, provided that procedures for proper disposal have been established according to applicable federal, state, local and institutional guidelines and procedures, and provided that appropriate records of disposal are kept.

4.7 Administration

See manufacturer's guidelines as described in the package insert.

4.8 Dose modification for Pembrolizumab

- No dose reductions of pembrolizumab will be permitted. Immune related adverse events requiring more than a 12 week interruption of pembrolizumab should discontinue study treatment (as outlined below in Table 5.3) but remain in follow up until the time of disease progression by RECIST 1.1 criteria or receipt of non-protocol therapy.

- Adverse events other than those listed below in the Table below should be attributed to the specific study medication whenever possible (Please see Section 6.3.2.5 Attribution of Adverse Events for more information).

- Pembrolizumab-related Grade 4 AEs requires permanent discontinuation of study treatment.

- For intolerable treatment-related Grade 2 AEs, pembrolizumab may be held per investigator discretion.

The following dose modification rules will be used with respect to potential toxicity. Toxicity will be assessed according to the NCI Common Terminology Criteria for Adverse Events.

Dose Interruptions/Discontinuation for Specific Adverse Events Related to Pembrolizumab

General instructions:

1. Severe and life-threatening irAEs should be treated with IV corticosteroids followed by oral steroids. Other immunosuppressive treatment should begin if the irAEs are not controlled by corticosteroids.
2. Study intervention must be permanently discontinued if the irAE does not resolve or the corticosteroid dose is not ≤10 mg/day within 12 weeks of the last study intervention treatment.
3. The corticosteroid taper should begin when the irAE is ≤ Grade 1 and continue at least 4 weeks.
4. If study intervention has been withheld, study intervention may resume after the irAE decreased to ≤ Grade 1 after corticosteroid taper.

irAEs	Toxicity Grade (CTCAE v5.0)	Action With Pembrolizumab	Corticosteroid and/or Other Therapies	Monitoring and Follow-up
Pneumonitis	Grade 2	Withhold	• Administer corticosteroids (initial dose of 1 to 2 mg/kg prednisone or equivalent) followed by taper<br>• Add prophylactic antibiotics for opportunistic infections	• Monitor participants for signs and symptoms of pneumonitis<br>• Evaluate participants with suspected pneumonitis with radiographic imaging and initiate corticosteroid treatment
	Recurrent Grade 2, Grade 3 or 4	Permanently discontinue		
Diarrhea/Colitis	Grade 2 or 3	Withhold	• Administer corticosteroids (initial dose of 1 to 2 mg/kg prednisone or equivalent) followed by taper	• Monitor participants for signs and symptoms of enterocolitis (ie, diarrhea, abdominal pain, blood or mucus in stool with or without fever) and of bowel perforation (ie, peritoneal signs

	Recurrent Grade 3 or Grade 4	Permanently discontinue		and ileus)<br>• Participants with ≥Grade 2 diarrhea suspecting colitis should consider GI consultation and performing endoscopy to rule out colitis<br>• Participants with diarrhea/colitis should be advised to drink liberal quantities of clear fluids. If sufficient oral fluid intake is not feasible, fluid and electrolytes should be substituted via IV infusion
AST or ALT Elevation or Increased Bilirubin	Grade 2 [a]	Withhold	• Administer corticosteroids (initial dose of 0.5 to 1 mg/kg prednisone or equivalent) followed by taper	• Monitor with liver function tests (consider weekly or more frequently until liver enzyme value returned to baseline or is stable)
	Grade 3 [b] or 4 [c]	Permanently discontinue	• Administer corticosteroids (initial dose of 1 to 2 mg/kg prednisone or equivalent) followed by taper	
T1DM or Hyperglycemia	New onset T1DM or Grade 3 or 4 hyperglycemia associated with evidence of β-cell failure	Withhold [d]	• Initiate insulin replacement therapy for participants with T1DM<br>• Administer antihyperglycemic in participants with hyperglycemia	• Monitor participants for hyperglycemia or other signs and symptoms of diabetes
Hypophysitis	Grade 2	Withhold	• Administer corticosteroids and initiate	• Monitor for signs and symptoms of hypophysitis

	Grade 3 or 4	Withhold or permanently discontinue [d]	hormonal replacements as clinically indicated	(including hypopituitarism and adrenal insufficiency)
Hyperthyroidism	Grade 2	Continue	• Treat with nonselective beta-blockers (eg, propranolol) or thionamides as appropriate	• Monitor for signs and symptoms of thyroid disorders
	Grade 3 or 4	Withhold or permanently discontinue [d]		
Hypothyroidism	Grade 2, 3 or 4	Continue	• Initiate thyroid replacement hormones (eg, levothyroxine or liothyronine) per standard of care	• Monitor for signs and symptoms of thyroid disorders
Nephritis: grading according to increased creatinine or acute kidney injury	Grade 2	Withhold	• Administer corticosteroids (prednisone 1 to 2 mg/kg or equivalent) followed by taper	• Monitor changes of renal function
	Grade 3 or 4	Permanently discontinue		
Neurological Toxicities	Grade 2	Withhold	• Based on severity of AE administer corticosteroids	• Ensure adequate evaluation to confirm etiology and/or exclude other causes
	Grade 3 or 4	Permanently discontinue		
Myocarditis	Grade 1	Withhold	• Based on severity of AE administer corticosteroids	• Ensure adequate evaluation to confirm etiology and/or exclude other causes
	Grade 2, 3 or 4	Permanently discontinue		
Exfoliative Dermatologic Conditions	Suspected SJS, TEN, or DRESS	Withhold	• Based on severity of AE administer	• Ensure adequate evaluation to confirm etiology

	Confirmed SJS, TEN, or DRESS	Permanently discontinue	corticosteroids	or exclude other causes
All Other irAEs	Persistent Grade 2	Withhold	• Based on severity of AE administer corticosteroids	• Ensure adequate evaluation to confirm etiology or exclude other causes
	Grade 3	Withhold or discontinue based on the event [e]		
	Recurrent Grade 3 or Grade 4	Permanently discontinue		

AE(s)=adverse event(s); ALT= alanine aminotransferase; AST=aspartate aminotransferase; CTCAE=Common Terminology Criteria for Adverse Events; DRESS=Drug Rash with Eosinophilia and Systemic Symptom; GI=gastrointestinal; IO=immuno-oncology; ir=immune related; IV=intravenous; SJS=Stevens-Johnson Syndrome; T1DM=type 1 diabetes mellitus; TEN=Toxic Epidermal Necrolysis; ULN=upper limit of normal.

Note: Non-irAE will be managed as appropriate, following clinical practice recommendations.

[a] AST/ALT: >3.0 to5.0 x ULN if baseline normal; >3.0 to 5.0 x baseline, if baseline abnormal; bilirubin:>1.5 to 3.0 x ULN if baseline normal; >1.5 to 3.0 x baseline if baseline abnormal

[b] AST/ALT: >5.0 to 20.0 x ULN, if baseline normal; >5.0 to 20.0 x baseline, if baseline abnormal; bilirubin:>3.0 to 10.0 x ULN if baseline normal; >3.0 to 10.0 x baseline if baseline abnormal

[c] AST/ALT: >20.0 x ULN, if baseline normal; >20.0 x baseline, if baseline abnormal; bilirubin: >10.0 x ULN if baseline normal; >10.0 x baseline if baseline abnormal

[d] The decision to withhold or permanently discontinue pembrolizumab is at the discretion of the investigator or treating physician. If control achieved or ≤ Grade 2, pembrolizumab may be resumed.

[e] Events that require discontinuation include, but are not limited to: encephalitis and other clinically important irAEs (e.g. vasculitis and schlerosing cholangitis).

Supportive Care Measures and Concomitant Medications

Patients who have not had prior bilateral orchiectomy must continue LHRH analogue treatment to maintain castrate level of testosterone during the course of study treatment.

The use of bone-modifying agents (e.g. zoledronic acid, denosumab) is permitted as clinically indicated.

The use of growth factors (e.g. G-CSF) is allowed as clinically indicated for the treatment of Grade ≥ 3 cytopenias.

The use of any other systemic anti-cancer therapies other than outlined in the protocol is prohibited during the course of study treatment.

Focal radiation treatment to symptomatic metastases following C1D1 of study will require discontinuation from study.

Suggested supportive care measures for the management of AEs with potential immunologic etiology are outlined below. Where appropriate, these guidelines include the use of oral or intravenous treatment with corticosteroids as well as additional anti-inflammatory agents if symptoms do not improve with administration of corticosteroids. Note that several courses of steroid tapering may be necessary as symptoms may worsen when the steroid dose is decreased. For each AE, attempts should be made to rule out other causes such as metastatic disease or bacterial or viral infection, which might require additional supportive care. The treatment guidelines are intended to be applied when the investigator determines the events to be related to pembrolizumab.

It may be necessary to perform conditional procedures such as bronchoscopy, endoscopy, or skin photography as part of the evaluation of the event.

- **Pneumonitis**:
    - For **Grade 2 events**, treat with systemic corticosteroids (e.g. oral prednisone 1 mg/kg or equivalent). When symptoms improve to Grade 1 or less, steroid taper should be started and continued over no less than 4 weeks.
    - For **Grade 3-4 events**, immediately treat with IV steroids (e.g. solumedrol 1-2 mg/kg every 6-8 hours). Administer additional anti-inflammatory measures, as needed. When symptoms improve to Grade 1 or less, steroid taper should be started and continued over no less than 4 weeks.
    - Add prophylactic antibiotics for opportunistic infections in the case of prolonged steroid administration.
- **Diarrhea/Colitis**:

Subjects should be carefully monitored for signs and symptoms of enterocolitis (such as diarrhea, abdominal pain, blood or mucus in stool, with or without fever) and of bowel perforation (such as peritoneal signs and ileus).

- All subjects who experience diarrhea/colitis should be advised to drink liberal quantities of clear fluids. If sufficient oral fluid intake is not feasible, fluid and electrolytes should be administered via IV infusion. For Grade 2 or higher diarrhea, consider gastroenterology consultation and endoscopy to confirm or rule out colitis.
- For **Grade 2 diarrhea/colitis,** administer oral corticosteroids (e.g. oral prednisone 1 mg/kg or equivalent). When symptoms improve to Grade 1 or less, steroid taper should be started and continued over no less than 4 weeks.
- For **Grade 3 or 4 diarrhea/colitis,** treat with IV steroids (e.g. solumedrol 1-2 mg/kg every 6-8 hours) followed by high dose oral steroids. When symptoms improve to Grade 1 or less, steroid taper should be started and continued over no less than 4 weeks.

- **Type 1 diabetes mellitus (if new onset, including diabetic ketoacidosis [DKA]) or ≥ Grade 3 Hyperglycemia, if associated with ketosis (ketonuria) or metabolic acidosis (DKA)**
  - For **T1DM** or **Grade 3-4** Hyperglycemia
    - Insulin replacement therapy is recommended for Type I diabetes mellitus and for Grade 3-4 hyperglycemia associated with metabolic acidosis or ketonuria.
    - Evaluate subjects with serum glucose and a metabolic panel, urine ketones, glycosylated hemoglobin, and C-peptide.

- **Hypophysitis**:
  - For **Grade 2** events, treat with corticosteroids (e.g oral prednisone 1 mg/kg/day). When symptoms improve to Grade 1 or less, steroid taper should be started and continued over no less than 4 weeks. Replacement of appropriate hormones may be required as the steroid dose is tapered.
  - For **Grade 3-4** events, treat with an initial dose of IV corticosteroids (e.g solumedrol 1-2 mg/kg every 6-8 hours) for 24-48 hours, followed by high dose oral corticosteroids. When symptoms improve to Grade 1 or less, steroid taper should be started and continued over no less than 4 weeks. Replacement of appropriate hormones may be required as the steroid dose is tapered.

- **Hyperthyroidism or Hypothyroidism**:

Thyroid disorders can occur at any time during treatment. Monitor subjects for changes in thyroid function (at the start of treatment, periodically during treatment, and as indicated based on clinical evaluation) and for clinical signs and symptoms of thyroid disorders.

- **Grade 2** hyperthyroidism events and **Grade 2-4** hypothyroidism:

        - In hyperthyroidism, non-selective beta-blockers (e.g., propranolol) are suggested as initial therapy.
        - In hypothyroidism, thyroid hormone replacement therapy, with levothyroxine or liothyronine, is indicated per SOC.
    - **Grade 3-4** hyperthyroidism
        - Treat with an initial dose of IV corticosteroids followed by oral corticosteroids. When symptoms improve to Grade 1 or less, steroid taper should be started and continued over no less than 4 weeks. Replacement of appropriate hormones may be required as the steroid dose is tapered.

- **Hepatic**:
    - For **Grade 2** events, monitor liver function tests more frequently until returned to baseline values (consider weekly).
        - Treat with IV or oral corticosteroids (e.g. prednisone 1 mg/kg/day or equivalent).
    - For **Grade 3-4** events, treat with IV corticosteroids for 24 to 48 hours (e.g. solumedrol 1-2 mg/kg every 6-8 hours), followed by transition to oral high dose steroids (e.g. prednisone 1 mg/kg/day or equivalent).
    - When symptoms improve to Grade 1 or less, a steroid taper should be started and continued over no less than 4 weeks.

- **Renal Failure or Nephritis**:
    - For **Grade 2** events, treat with oral corticosteroids (e.g. prednisone 1 mg/kg/day).
    - For **Grade 3-4** events, treat with IV corticosteroids (e.g. solumedrol 1-2 mg/kg every 6-8 hours) for 24-48 hours followed by transition to oral high dose steroid (e.g. prednisone 1 mg/kg/day or equivalent).
    - When symptoms improve to Grade 1 or less, steroid taper should be started and continued over no less than 4 weeks.

- **Management of Infusion Reactions**: Signs and symptoms usually develop during or shortly after drug infusion and generally resolve completely within 24 hours of completion of infusion.

The table below shows treatment guidelines for subjects who experience an infusion reaction associated with administration of pembrolizumab.

Infusion Reaction Treatment Guidelines

**NCI CTCAE Grade**	**Treatment**	**Premedication at subsequent dosing**
Grade 1<br><br>Mild reaction; infusion interruption not indicated; intervention not indicated	Increase monitoring of vital signs as medically indicated until the subject is deemed medically stable in the opinion of the investigator.	None
Grade 2<br><br>Requires infusion interruption but responds promptly to symptomatic treatment (e.g., antihistamines, NSAIDS, narcotics, IV fluids); prophylactic medications indicated for < =24 hrs	**Stop Infusion and monitor symptoms.** Additional appropriate medical therapy may include but is not limited to:<br><br>IV fluids Antihistamines NSAIDS<br><br>Acetaminophen Narcotics<br><br>Increase monitoring of vital signs as medically indicated until the subject is deemed medically stable in the opinion of the investigator.<br><br>If symptoms resolve within one hour of stopping drug infusion, the infusion may be restarted at 50% of the original infusion rate (e.g., from 100 mL/hr to 50 mL/hr). Otherwise dosing will be held until symptoms resolve and the subject should be premedicated for the next scheduled dose.	Subject may be premedicated 1.5h (± 30 minutes) prior to infusion of pembrolizumab (MK-3475) with:<br><br>Diphenhydramine 50 mg po (or equivalent dose of antihistamine).<br><br>Acetaminophen 500-1000 mg po (or equivalent dose of antipyretic).

NCI CTCAE Grade	Treatment	Premedication at subsequent dosing
Grades 3 or 4<br><br>Grade 3:<br><br>Prolonged (i.e., not rapidly responsive to symptomatic medication and/or brief interruption of infusion); recurrence of symptoms following initial improvement; hospitalization indicated for other clinical sequelae (e.g., renal impairment, pulmonary infiltrates)<br><br>Grade 4:<br><br>Life-threatening; pressor or ventilatory support indicated	**Stop Infusion.**<br><br>Additional appropriate medical therapy may include but is not limited to:<br><br>IV fluids Antihistamines NSAIDS<br><br>Acetaminophen Narcotics Oxygen Pressors Corticosteroids Epinephrine<br><br>Increase monitoring of vital signs as medically indicated until the subject is deemed medically stable in the opinion of the investigator.<br><br>Hospitalization may be indicated.<br><br>**Subject is permanently discontinued from further trial treatment administration.**	No subsequent dosing
Appropriate resuscitation equipment should be available in the room and a physician readily available during the period of drug administration.		

5 Study Procedures

A written, signed, informed consent form (ICF) and a Health Insurance Portability and Accountability Act (HIPAA) authorization must be obtained before any study-specific assessments are initiated. A copy of the signed ICF will be given to the subject and a copy will be filed in the medical record. The original will be kept on file with the study records.

The Study Calendar summarizes the trial procedures to be performed at each visit according to the assigned Treatment Schedule (Schedule 1, Schedule 2, or Schedule 3, respectively). Screening assessments must be performed within 28 days prior to the initiation of protocol therapy. Any results falling outside of the reference ranges may be repeated at the discretion of the investigator. All on-study procedures are allowed a window of ± 3 days unless otherwise noted. Treatment or visit delays for public holidays or weather conditions do not constitute a protocol violation.

Individual trial procedures are described in the study schedule below. It may be necessary to perform these trial procedures at unscheduled time points if deemed clinically necessary by the investigator.

Furthermore, additional evaluations/testing may be deemed necessary by the investigator for reasons related to subject safety. In some cases, such evaluation/testing may be potentially sensitive in nature (e.g. HIV, hepatitis C), and thus local regulations may require that additional informed consent be obtained from the subject. In these cases, such evaluations/testing will be performed in accordance with those regulations.

5.1 Discontinuation of Therapy

Study treatment will be discontinued at the first occurrence of one of the following:

- Radiographic progression by RECIST 1.1/PCWG3 criteria, confirmed by repeat scans performed at least 4 weeks following first scans showing progression.
- Unequivocal clinical progression
  Note: Patients without clinical or radiographic evidence of disease progression, with PSA-only progression, should continue to receive protocol therapy.
- Unacceptable toxicity (as described in section 4.8)
- Recurrent Grade 2 pneumonitis
- Patient withdrawal from study
- Investigator withdrawal of patient due to concerns for patient compliance with study protocol procedures
- Receipt of non-protocol systemic anti-prostate cancer therapy
- Death
- Completion of 35 treatments (approximately 2 years) with pembrolizumab
  Note: The number of treatments is calculated starting with the first dose. Participants who stop the combination or pembrolizumab after receiving 35 doses may be eligible for retreatment if they progress after stopping study treatment provided they meet the requirements detailed below. Participants may be retreated in the Second Course Phase (Retreatment) for up to an additional 17 cycles (approximately 1 year).

Second Course of Pembrolizumab *

All participants who stop study treatment with SD or better may be eligible for up to an additional 17 cycles (approximately 1 year) of pembrolizumab treatment if they progress after stopping study treatment from the initial treatment phase. This retreatment is termed the Second Course Phase of this study and is only available if the study remains open and the participant meets the following conditions:

Either

- Stopped initial treatment with study treatment after attaining an investigator-determined confirmed CR based on RECIST 1.1, and
  - Was treated with at least 8 cycles of study treatment before discontinuing treatment, and
  - Received at least 2 treatments with pembrolizumab beyond the date when the initial CR was declared

OR

- Had SD, PR, or CR and stopped study treatment after completion of 35 administrations (approximately 2 years) of study treatment for reasons other than disease progression or intolerability

AND

- Experienced an investigator-determined radiographic disease progression by RECIST 1.1 after stopping initial treatment, and
  - No new anticancer treatment was administered after the last dose of study treatment, and
  - The participant meets all of the safety parameters listed in the inclusion criteria and none of the safety parameters listed in the exclusion criteria, and
  - The study is ongoing

An objective response or disease progression that occurs during the Second Course Phase for a participant will not be counted as an event for the primary analysis of either endpoint in this study.

**Note: patients must have measurable disease at the start of protocol treatment to be eligible for this provision.*

5.2 Study Calendar: Schedule 1 (^{177}Lu-PSMA-617 prior to Pembrolizumab)

	Screening (Day -28 to -1 unless otherwise noted)	Treatment					End of Treatment	Safety Follow up	
			Cycle 1			Cycle 2+ (+/- 3 days unless otherwise noted)		30 Days (+/- 7 days) post-last dose	90 Days (+/- 14 days) post last dose
		D1 (-3 days)	D2	D8 (+/- 1 day)	D15 (+/- 1 day)	D1 (+/- 3 days)	+ 21 days		
**Informed consent**	x								
**Medical history**	x								
**Baseline conditions**	x								
**Concurrent medications**	x	x		x	x	x	x	x	x
**Height**	x								
**Weight**	x	x		x	x	x	x	x	x
**ECOG Performance status**	x	x		x	x	x	x	x	x
**Physical exam and vital signs**	x	x		x	x	x	x	x	x
**CBC and Serum Chemistry Panel**[1,2]	x	x		x	x	x[3]	x	x	x
**Thyroid function tests**[2,4]	x					x	x	x	x
**PSA**[2]	x	x				x	x	x	x
**Testosterone**	x								
**^{68}Ga-PSMA-11 PET/CT or PET/MR**	x[5]						x		
**Disease assessment**	x					x	x		

---

1 Chemistry panel to include: sodium, potassium, chloride, bicarbonate, BUN/creatinine, glucose, calcium, total bilirubin, AST, ALT, alkaline phosphatase

2 Labs may be collected within a window of up to 3 days prior to each dose of pembrolizumab.

3 CBC + differential will be measured on C1D1, C1D8, C1D15, C2D1, C2D8, C2D15, Day 1 of C3+, and additionally as clinically indicated.

4 TSH (with reflex free T3/T4) testing to be performed during Screening, Cycle 2 Day 1, and Day 1 of every other cycle thereafter.

5 Allow for a 12-week window for the screening PSMA PET scan. PSMA PET at End of Treatment is optional.

	Screening (Day -28 to -1 unless otherwise noted)		Treatment				End of Treatment	Safety Follow up	
			Cycle 1			Cycle 2+ (+/- 3 days unless otherwise noted)		30 Days (+/- 7 days) post-last dose	90 Days (+/- 14 days) post last dose
		D1 (-3 days)	D2	D8 (+/- 1 day)	D15 (+/- 1 day)	D1 (+/- 3 days)	+ 21 days		
**(CT/MR, BS)**[6]									
**Tumor Biopsy**[7]	x					x			
**Whole blood for immune correlatives**[8]		x				x	x		
**^{177}Lu-PSMA-617 infusion**		x							
**SPECT/CT Dosimetry**[9]			X						
**Normal Saline**		x							
**Anti-emetic pre-medication**		x							
**Pembrolizumab infusion**						x			
**AE assessment**		x		x	x	x	x	x	x

---

6 Tumor assessment will be performed with CT chest/and/pelvis + whole body bone scan during Screening, C4D1 (-14d), C7D1 (-14d), and Day 1 (-14d) of every 4 cycles thereafter. If disease progression by RECIST 1.1/PCWG3, confirmatory scans are required ≥ 4 weeks following first scan showing progression.

7 Tumor biopsy will be performed during Screening and within 7 days preceding C2D1 visit. Screening biopsy must be performed prior to initiation of study treatment but is not subject to 28-day screening window. Bone biopsies are acceptable if no soft tissue lesion is amenable for biopsy. The same lesion should be selected for the follow up biopsy whenever feasible.

8 Whole blood (40 mL in Na-Heparin green top tube) will be collected on C1D1 (pre-dose), C2D1 (pre-dose), C3D1 (pre-dose), on Day 1 of every 3 cycles thereafter (e.g. C6D1, C9D1, etc), and at the time of disease progression

9 SPECT/CT dosimetry scan is optional.

5.3 Study Calendar – Schedule 2 (Concurrent Dosing)

	Screening (Day -28 to -1 unless otherwise noted)	Treatment					End of Treatment	Safety Follow up	
		Cycle 1				Cycle 2+ (+/- 3 days unless otherwise noted)		30 Days (+/- 7 days) post-last dose	90 Days (+/- 14 days) post last dose
		D1 (-3 days)	D2	D8 (+/- 1 day)	D15 (+/- 1 day)	D1 (+/- 3 days)	+ 21 days		
**Informed consent**	x								
**Medical history**	x								
**Baseline conditions**	x								
**Concurrent medications**	x	x		x	x	x		x	x
**Height**	x								
**Weight**	x	x		x	x	x	x	x	x
**ECOG Performance status**	x	x		x	x	x	x	x	x
**Physical exam and vital signs**	x	x		x	x	x	x	x	x
**CBC and Serum Chemistry Panel**[1,2]	x	x		x	x	x[3]	x	x	x
**Thyroid function tests**[2,4]	x					x	x	x	x
**PSA**[2]	x	x				x	x	x	x
**Testosterone**	x								
**^{68}Ga-PSMA-11 PET/CT or PET/MR**	x[5]						x		
**Disease assessment (CT/MR,**	x					x	x		

[1] Chemistry panel to include: sodium, potassium, chloride, bicarbonate, BUN/creatinine, glucose, calcium, total bilirubin, AST, ALT, alkaline phosphatase

[2] Labs may be collected within a window of up to 3 days prior to each dosing of pembrolizumab.

[3] CBC + differential will be measured on C1D1, C1D8, C1D15, C2D1, C2D8, C2D15, Day 1 of C3+, and additionally as clinically indicated.

[4] TSH (with reflex free T3/T4) testing to be performed during Screening, Cycle 1 Day 1, Cycle 2 Day 1, and Day 1 of every other cycle thereafter.

[5] Allow for a 12-week window for the screening PSMA PET scan. PSMA PET scan at End of Treatment is optional.

	Screening (Day -28 to -1 unless otherwise noted)	Treatment					End of Treatment	Safety Follow up	
		Cycle 1				Cycle 2+ (+/- 3 days unless otherwise noted)		30 Days (+/- 7 days) post-last dose	90 Days (+/- 14 days) post last dose
		D1 (-3 days)	D2	D8 (+/- 1 day)	D15 (+/- 1 day)	D1 (+/- 3 days)	+ 21 days		
**BS)**[6]									
**Tumor Biopsy**[7]	x					x			
**Whole blood for immune correlatives**[8]		x				x	x		
**^{177}Lu-PSMA-617 infusion**		x							
**SPECT/CT Dosimetry**[9]			X						
**Normal Saline**		x							
**Anti-emetic pre-medication**		x							
**Pembrolizumab infusion**		x				x			
**AE assessment**		x		x	x	x	x	x	x

6 Tumor assessment will be performed with CT chest/and/pelvis + whole body bone scan during Screening, C4D1 (-14d), C7D1 (-14d), and Day 1 (-14d) of every 4 cycles thereafter. If disease progression by RECIST 1.1/PCWG3, confirmatory scans are required ≥ 4 weeks following first scan showing progression.

7 Tumor biopsy will be performed during Screening and within 7 days preceding C2D1 visit. Screening biopsy must be performed prior to initiation of study treatment but is not subject to 28-day screening window. Bone biopsies are acceptable if no soft tissue lesion is amenable for biopsy. The same lesion should be selected for the follow up biopsy whenever feasible.

8 Whole blood (40 mL in Na-Heparin green top tube) will be collected on C1D1 (pre-dose), C2D1 (pre-dose), C3D1 (pre-dose), on Day 1 of every 3 cycles thereafter (e.g. C6D1, C9D1, etc), and at the time of disease progression.

9 SPECT/CT dosimetry scan is optional.

5.4 Study Calendar – Schedule 3 (^{177}Lu-PSMA-617 following Pembrolizumab)

	Screening (Day -49 to -21 unless otherwise noted)	Treatment						End of Treatment	Safety Follow up	
			Cycle 1				Cycle 2+ (+/- 3 days unless otherwise noted)		30 Days (+/- 7 days) post-last dose	90 Days (+/- 14 days) post last dose
		D-21 (-3 days)	D1 (- 3 days)	D2	D8 (+/- 1 day)	D15 (+/- 1 day)	D1 (+/- 3 days)	+ 21 days		
**Informed consent**	x									
**Medical history**	x									
**Baseline conditions**	x									
**Concurrent medications**	x	x	x		x	x	x		x	x
**Height**	x									
**Weight**	x	x	x		x	x	x	x	x	x
**ECOG Performance status**	x	x	x		x	x	x	x	x	x
**Physical exam and vital signs**	x	x	x		x	x	x	x	x	x
**CBC and Serum Chemistry Panel**[1,2]	x	x	x		x	x	x[3]	x	x	x
**Thyroid function tests**[2,4]	x	x					x	x	x	x
**PSA**[2]	x	x	x				x	x	x	x
**Testosterone**	x									
**^{68}Ga-PSMA-11 PET/CT or PET/MR**[5]	x[5]							x		

1 Chemistry panel to include: sodium, potassium, chloride, bicarbonate, BUN/creatinine, glucose, calcium, total bilirubin, AST, ALT, alkaline phosphatase

2 Labs may be collected within a window of up to 3 days prior to each dosing of pembrolizumab.

3 CBC + differential will be measured on C1D1, C1D8, C1D15, C2D1, C2D8, C2D15, Day 1 of C3+, and additionally as clinically indicated.

4 TSH (with reflex free T3/T4) testing to be performed during Screening, Cycle 1 Day 1, Cycle 2 Day 1, and Day 1 of every other cycle thereafter.

5 Allow for a 12-week window for the screening PSMA PET scan. PSMA PET scan at End of Treatment is optional.

**Disease assessment (CT/MR, BS)[6]**	x						x	x		
**Tumor Biopsy[7]**	x						x			
**Whole blood for immune correlatives[8]**		x	x				x	x		
**^{177}Lu-PSMA-617 infusion**			x							
**SPECT/CT Dosimetry[9]**				X						
**Normal Saline**			x							
**Anti-emetic pre-medication**			x							
**Pembrolizumab infusion**		x	x				x			
**AE assessment**		x	x		x	x	x	x	x	x

6 Tumor assessment will be performed with CT chest/and/pelvis + whole body bone scan during Screening, C4D1 (-14d), C7D1 (-14d), and Day 1 (-14d) of every 4 cycles thereafter. If disease progression by RECIST 1.1/PCWG3, confirmatory scans are required ≥ 4 weeks following first scan showing progression.

7 Tumor biopsy will be performed during Screening and within 14 days preceding C3D1 visit. Screening biopsy must be performed prior to initiation of study treatment but is not subject to 28-day screening window. Bone biopsies are acceptable if no soft tissue lesion is amenable for biopsy. The same lesion should be selected for the follow up biopsy whenever feasible. PSMA-avid lesion should be prioritized for biopsy; non-PSMA avid lesion may be used if no PSMA-avid lesion is accessible.

8 Whole blood (40 mL in Na-Heparin green top tube) will be collected on C1D-21 (pre-dose), C1D1 (pre-dose), C2D1 (pre-dose), C3D1 (pre-dose), on Day 1 of every 3 cycles thereafter (e.g. C6D1, C9D1, etc), and at the time of disease progression.

9 SPECT/CT dosimetry scan is optional.

6 Reporting and Documentation of Results

6.1 Evaluation of efficacy (or Activity)

6.1.1 Definitions

Evaluable for toxicity

All patients will be evaluable for toxicity from the time of the first study drug administration.

6.2 Evaluation of Safety

Analyses will be performed for all patients receiving the radiotracer. The study will use the CTCAE v4.0 for reporting of adverse events.

Any patient with Grade ≥3 hematologic toxicity will be followed with weekly CBC with differential until resolved to Grade 2 or lower.

6.3 Definition of Adverse Events

6.3.1 Adverse Event

An adverse event (also known as an adverse experience) is defined as any untoward medical occurrence associated with the use of a drug in humans, whether or not considered drug related. More specifically, an adverse event (can be any unfavorable and unintended sign (e.g., an abnormal laboratory finding), symptom, or disease temporally associated with the use of a drug, without any judgment about causality. An adverse event can arise from any use of the drug (e.g., off-label use, use in combination with another drug) and from any route of administration, formulation, or dose, including an overdose.

6.3.2 Adverse Reaction

An adverse reaction is defined as any adverse event caused by the use of a drug. Adverse reactions are a subset of all suspected adverse reactions for which there is reason to conclude that the drug caused the event.

6.3.2.1 Suspected

A suspected adverse reaction is defined as any adverse event for which there is a reasonable possibility that the drug caused the adverse event. For the purposes of IND safety reporting, "reasonable possibility" indicates that there is evidence to suggest a causal relationship between the drug and the adverse event. A suspected adverse reaction implies a lesser degree of certainty about causality than an adverse reaction.

6.3.2.2 Unexpected

An adverse event or suspected adverse reaction is considered *unexpected* if it is not listed in the investigator brochure or package insert(s), or is not listed at the specificity or severity that has been observed, or, if an investigator brochure is not required or available, is not consistent with the risk information described in the general investigational plan or elsewhere in the current application.

"Unexpected," as used in this definition, also refers to adverse events or suspected adverse reactions that are mentioned in the investigator brochure as occurring with a class of drugs or as anticipated from the pharmacological properties of the drug, but are not specifically mentioned as occurring with the particular drug under investigation.

Adverse events that would be anticipated to occur as part of the disease process are considered *unexpected* for the purposes of reporting because they would not be listed in the investigator brochure. For example, a certain number of non-acute deaths in a cancer trial would be anticipated as an outcome of the underlying disease, but such deaths would generally not be listed as a suspected adverse reaction in the investigator brochure.

6.3.2.3 Serious

An adverse event or suspected adverse reaction is considered *serious* if, in the view of either the investigator or sponsor, it results in any of the following outcomes:

- Death
- Life-threatening adverse event
- Inpatient hospitalization or prolongation of existing hospitalization
- A persistent or significant incapacity or substantial disruption of the ability to conduct normal life function
- Congenital anomaly/birth defect
- Is a new cancer
- Is associated with an overdose

Important medical events that may not result in death, are life-threatening, or require hospitalization may be considered serious when, based upon appropriate medical judgment, they may jeopardize the patient or subject and may require medical or surgical intervention to prevent one of the outcomes listed in this definition. Examples of such medical events include allergic bronchospasm requiring intensive treatment in an emergency room or at home, blood dyscrasias or convulsions that do not result in inpatient hospitalization, or the development of drug dependency or drug abuse.

6.3.2.4 Life-threatening

An adverse event or suspected adverse reaction is considered life-threatening if, in the view of either the investigator or sponsor, its occurrence places the patient or subject at immediate risk of death. It does not include an adverse event or suspected adverse reaction that, had it occurred in a more severe form, might have caused death.

6.3.2.5 Attribution of Adverse Events

The toxicity profiles of the two investigational agents are largely non-overlapping, which should facilitate adverse event attribution to the specific agent. Hematologic toxicity will be attributed to Lu-PSMA-617 unless there is clear evidence of autoimmune cytopenia. Xerostomia will be attributed to Lu-PSMA-617 unless clear evidence of other autoimmune sequelae. Conversely, immune-related AEs such as colitis, pneumonitis, transaminitis will be attributed to pembrolizumab. In cases where attribution to a specific agent is not possible, the timing of onset in relation to timing of initiation of Lu-PSMA and pembrolizumab will be utilized. In cases where adverse event attribution to a single agent is not possible, attribution will be to study regimen

and dose modification criteria for both agents will be utilized. The PI will have the final determination of adverse event attribution that is reported.

AEs associated with pembrolizumab exposure, including coadministration with additional compounds, may represent an immunologic aetiology. These immune-related AEs (irAEs) may occur shortly after the first dose or several months after the last dose of pembrolizumab/combination treatment and may affect more than one body system simultaneously. Therefore, early recognition and initiation of treatment is critical to reduce complications. Based on existing clinical study data, most irAEs were reversible and could be managed with interruptions of pembrolizumab/combination treatment, administration of corticosteroids and/or other supportive care. For suspected irAEs, ensure adequate evaluation to confirm etiology or exclude other causes. Additional procedures or tests such as bronchoscopy, endoscopy, skin biopsy may be included as part of the evaluation. Dose modification and toxicity management guidelines for irAEs associated with pembrolizumab/combination treatment are provided in AE Table.

Attribution of Toxicity:

When study interventions are administered in combination, attribution of an adverse event to a single component is likely to be difficult. Therefore, while the investigator may attribute a toxicity event [to the combination, to ^{177}Lu-PSMA-617 alone or to pembrolizumab alone, for adverse events listed in AE Table 6, both interventions must be held according to the criteria in AE Table Dose Modification and Toxicity Management Guidelines for Immune-Related Adverse Events Associated with Pembrolizumab.

Holding Study Interventions:

When study interventions are administered in combination, if the AE is considered immune-related, both interventions should be held according to recommended dose modifications.

Restarting Study Interventions:

Participants may not have any dose modifications (no change in dose or schedule) of pembrolizumab in this study, as described in AE Table.

- If the toxicity does not resolve or the criteria for resuming treatment are not met, the participant must be discontinued from all study interventions.
- If the toxicities do resolve and conditions are aligned with what is defined in AE Table, the combination of ^{177}Lu-PSMA-617 and pembrolizumab may be restarted at the discretion of the investigator. In these cases where the toxicity is attributed to the combination or to ^{177}Lu-PSMA-617 alone, re-initiation of pembrolizumab as a monotherapy may be considered at the principal investigator's discretion .

6.4 Recording of an Adverse Event

All grade 3 and above adverse events will be recorded using the NCI CTCAE v4.0. The Investigator will assign attribution of the possible association of the event with use of the investigational drug.

Relationship	Attribution	Description
Unrelated to investigational drug/intervention	Unrelated	The AE *is clearly NOT related* to the intervention
	Unlikely	The AE *is doubtfully related* to the intervention
Related to investigational drug/intervention	Possible	The AE *may be related* to the intervention
	Probable	The AE *is likely related* to the intervention
	Definite	The AE *is clearly related* to the intervention

Signs or symptoms reported as adverse events will be graded and recorded by the Investigator according to the CTCAE. When specific adverse events are not listed in the CTCAE they will be graded by the Investigator as *none*, *mild*, *moderate* or *severe* according to the following grades and definitions:

Grade 0 No AE (or within normal limits)

Grade 1 Mild; asymptomatic or mild symptoms; clinical or diagnostic observations only; intervention not indicated

Grade 2 Moderate; minimal, local, or noninvasive intervention (e.g., packing, cautery) indicated; limiting age-appropriate instrumental activities of daily living (ADL)

Grade 3: Severe or medically significant but not immediately life-threatening; hospitalization or prolongation of hospitalization indicated; disabling; limiting self-care ADL

Grade 4: Life-threatening consequences; urgent intervention indicated

Grade 5: Death related to AE

6.5 Follow-up of Adverse Events

All adverse events will be followed with appropriate medical management until resolved. Patients removed from study for unacceptable adverse events will be followed until resolution or stabilization of the adverse event. For selected adverse events for which administration of the investigational drug was stopped, a re-challenge of the subject with the investigational drug may be conducted if considered both safe and ethical by the Investigator.

6.6 Expedited Reporting

Reporting to the Data and Safety Monitoring Committee

If a death occurs during the treatment phase of the study or within 30 days after the last administration of the study drug(s) and it is determined to be related either to the study drug(s) or to a study procedure, the Investigator or his/her designee must notify the DSMC Chair (or qualified alternate) within 1 business day of knowledge of the event. The contact may be by phone or e-mail.

Reporting to UCSF Institutional Review Board

The Principal Investigator must report events meeting the UCSF IRB definition of "Unanticipated Problem" (UP) within 10 business days of his/her awareness of the event.

Expedited Reporting to the Food and Drug Administration

If the study is being conducted under an IND, the Sponsor-Investigator is responsible for determining whether or not the suspected adverse reaction meets the criteria for expedited reporting in accordance with Federal Regulations (21 CFR §312.32).

The Investigator must report in an IND safety report any suspected adverse reaction that is both serious and unexpected. The Sponsor-Investigator needs to ensure that the event meets all three definitions (Section 7.3):

- Suspected adverse reaction
- Unexpected
- Serious

If the adverse event does not meet all three of the definitions, it should not be submitted as an expedited IND safety report.

The timeline for submitting an IND safety report to FDA is no later than **15 calendar days** after the Investigator determines that the suspected adverse reaction qualifies for reporting (21 CFR 312.32(c)(1)).

Any unexpected fatal or life-threatening suspected adverse reaction will be reported to FDA no later than **7 calendar days** after the Investigator's initial receipt of the information (21 CFR 312.32(c)(2)).

Any relevant additional information that pertains to a previously submitted IND safety report will be submitted to FDA as a Follow-up IND Safety Report without delay, as soon as the information is available (21 CFR 312.32(d)(2)).

Expedited Reporting to Merck

For the time period beginning at treatment allocation/randomization through 90 days following cessation of treatment, or 30 days following cessation of treatment if the participant initiates new anticancer therapy, whichever is earlier, any serious adverse event, or follow up to a serious adverse event, including death due to any cause whether or not related to the Merck product, must be reported within 2 working days to Merck Global Safety.

Additionally, any serious adverse event, considered by an investigator who is a qualified physician to be related to Merck product that is brought to the attention of the investigator at any time following consent through the end of the specified safety follow-up period specified in the paragraph above, or at any time outside of the time period specified in the previous paragraph also must be reported immediately to Merck Global Safety.

All participants with serious adverse events must be followed up for outcome.

SAE reports and any other relevant safety information are to be forwarded to the Merck Global Safety facsimile number: +1-215-661-6229

A copy of all 15 Day Reports and Annual Progress Reports is submitted as required by FDA. Investigators will cross reference this submission according to local regulations to the Merck

Investigational Compound Number (IND, CSA, etc.) at the time of submission. Additionally investigators will submit a copy of these reports to Merck & Co., Inc. (Attn: Worldwide Product Safety; FAX 215-661-6229) at the time of submission to FDA.

Expedited Reporting to PrimeVigilance

All AEs, SAEs, and SUSARs, related to ^{177}Lu-PSMA-617 will be reported to:

PrimeVigilance
The Surrey Research Park
26-28 Frederick Sanger Road
Guildford, Surrey GU2 7YD
Telephone: +44(0) 1483.566.462
Facsimile: 1.800.886.0743
Email: endocyte@primevigilance.com
SAEs and SUSARs must be reported to PrimeVigilance within 2 working days.

Definition of an Overdose for This Protocol and Reporting of Overdose to the Sponsor and to Merck

For purposes of this study, an overdose of pembrolizumab will be defined as any dose of 1,000 mg or greater (≥5 times the indicated dose). No specific information is available on the treatment of overdose of pembrolizumab. In the event of overdose, the participant should be observed closely for signs of toxicity. Appropriate supportive treatment should be provided if clinically indicated.

If an adverse event(s) is associated with ("results from") the overdose of a Merck product, the adverse event(s) is reported as a serious adverse event, even if no other seriousness criteria are met.

If a dose of Merck's product meeting the protocol definition of overdose is taken without any associated clinical symptoms or abnormal laboratory results, the overdose is reported as a non-serious Event of Clinical Interest (ECI), using the terminology "accidental or intentional overdose without adverse effect."

All reports of overdose with and without an adverse event must be reported within 24 hours to the Sponsor and within 2 working days hours to Merck Global Safety. (Attn: Worldwide Product Safety; FAX 215-661-6229)

Events of Clinical Interest

Selected non-serious and serious adverse events are also known as Events of Clinical Interest (ECI) and must be reported within 2 working days to Merck Global Safety. (Attn: Worldwide Product Safety; FAX 215-661-6229).

For the time period beginning when the consent form is signed until treatment allocation/randomization, any ECI, or follow up to an ECI, that occurs to any participant must be reported within 2 working days to Merck Global Safety if it causes the participant to be excluded from the trial, or is the result of a protocol-specified intervention, including but not limited to washout or discontinuation of usual therapy, diet, placebo treatment or a procedure.

For the time period beginning at treatment allocation/randomization through 90 days following cessation of treatment, or 30 days following cessation of treatment if the participant initiates new anticancer therapy, whichever is earlier, any ECI, or follow up to an ECI, whether or not related to Merck product, must be reported within 2 working days to Merck Global Safety.

Events of clinical interest for this trial include:

1. an overdose of Merck product, as defined above, that is not associated with clinical symptoms or abnormal laboratory results.

2. an elevated AST or ALT lab value that is greater than or equal to 3X the upper limit of normal and an elevated total bilirubin lab value that is greater than or equal to 2X the upper limit of normal and, at the same time, an alkaline phosphatase lab value that is less than 2X the upper limit of normal, as determined by way of protocol-specified laboratory testing or unscheduled laboratory testing.*

*Note: These criteria are based upon available regulatory guidance documents. The purpose of the criteria is to specify a threshold of abnormal hepatic tests that may require an additional evaluation for an underlying etiology.

7 Measurement of Response

Response and progression will be evaluated in this study using the international criteria proposed by the Response Evaluation Criteria in Solid Tumors (RECIST) 1.1 criteria, with use of PCWG criteria for assessment of new/pre-existing bone lesions. Changes in only the longest diameter (unidimensional measurement- LD) of the tumor lesions are used in the RECIST criteria.

Note: lesions are either measurable or non-measurable using the criteria provided below. The term “evaluable” in reference to measurability will not be used because it does not provide additional meaning or accuracy. All measurements should be taken and recorded in metric notation using a ruler or calipers. All baseline evaluations should be performed as closely as possible to the beginning of treatment and never more than 30 days before the beginning of the treatment. The same method of assessment and the same technique should be used to characterize each identified and reported lesion at baseline and during follow-up. Imaging-based evaluation is preferred to evaluation by clinical examination when both methods have been used to assess the antitumor effect of a treatment.

Clinical lesions. Clinical lesions will only be considered measurable when they are superficial (e.g., skin nodules and palpable lymph nodes). In the case of skin lesions, documentation by color photography, including a ruler to estimate the size of the lesion, is recommended.

Chest x-ray. Lesions on chest x-ray are acceptable as measurable lesions when they are clearly defined and surrounded by aerated lung. However, CT is preferable.

Conventional CT and MRI. These techniques should be performed with cuts of 10 mm or less in slice thickness contiguously. Spiral CT should be performed using a 5 mm contiguous reconstruction algorithm. This applies to tumors of the chest, abdomen, and pelvis. Head and neck tumors and those of extremities usually require specific protocols.

7.1 Measurable disease/ Target lesions

All measurable lesions (lesions that can be accurately measured in at least one dimension [longest diameter to be recorded] as ≥10mm with spiral CT) up to a maximum of 2 lesions per organ and 5 lesions total, representative of all involved organs, should be identified as target lesions and recorded and measured at baseline. Target lesions should be selected on the basis of their size (lesions with the longest diameter) and the suitability for accurate repeated measurements (either by imaging techniques or clinically). A sum of the longest diameter (LD) for all target lesions will be calculated and reported as the baseline sum LD. The baseline sum LD will be used as a reference by which to characterize the objective tumor response.

Lymph node metastases must measure 1.5 cm or greater in short axis diameter to be considered target lesions, while other target lesions must measure 1 cm or greater (with spiral CT scans).17

Complete Response (CR):	Disappearance of all target lesions
Partial Response (PR):	At least a 30% decrease in the sum of the longest diameter (LD) of target lesions, taking as reference the baseline sum LD
Progressive Disease (PD):	At least a 20% increase in the sum of the LD of target lesions, taking as reference the smallest sum LD recorded since the treatment started (including baseline LD), or the appearance of one or more new lesions
Stable Disease (SD):	Neither sufficient shrinkage to qualify for PR nor sufficient increase to qualify for PD, taking as reference the smallest sum LD since the treatment started (including

7.2 Evaluation of non-target lesions

Complete Response (CR):	Disappearance of all non-target lesions and normalization of tumor marker level
Incomplete Response/ Stable Disease (SD):	Persistence of one or more non-target lesion(s), and/or maintenance of tumor marker level above the normal limits
Progressive Disease (PD):	Appearance of one or more new lesions, and/or unequivocal progression of existing non-target lesions

Although a clear progression of non-target lesions only is exceptional, in such circumstances, the opinion of the treating physician should prevail and the progression status should be confirmed later on by the study chair.

7.3 Evaluation of best overall response

The best overall response is the best response recorded from the start of the treatment until disease progression/recurrence (taking as reference for progressive disease the smallest measurements recorded since the treatment started, including baseline; see table below). The patient's best response assignment will depend on the achievement of both measurement and confirmation criteria.

7.4 Confirmation

To be assigned a status of PR or CR, changes in tumor measurements must be confirmed by repeat studies no less than 4 weeks after the criteria for response are first met. In the case of SD, follow-up measurements must have met the SD criteria at least once after study entry at a minimum of 12 weeks after study entry.

7.5 Duration of Overall Response

The duration of overall response is measured from the time measurement criteria are met for CR/PR (whichever is first recorded) until the first date that recurrent or progressive disease is objectively documented (taking as reference for progressive disease the smallest measurements recorded since the treatment started). The duration of overall complete response is measured from the time measurement criteria are first met for CR until the first date that recurrent disease is objectively documented.

Target Lesions	Non-Target Lesions	New Lesions	Response
CR	CR	No	CR
CR	Incomplete response/SD	No	PR
PR	Non-PD	No	PR
SD	Non-PD	No	SD
PD	Any	Yes or No	PD
Any	PD	Yes or No	PD
Any	Any	Yes	PD

7.6 Evaluation of non-measurable bone disease per PCWG Criteria

Bone scans obtained after the baseline evaluation will be used to evaluate post-treatment changes. Bone scans obtained will be evaluated as either “no new lesions” or “new lesions” on the tumor measurement forms.

a. For the first scheduled reassessment: New lesions at the first scheduled evaluation (9 weeks) will require a confirmatory bone scan 6 or more weeks later. If no new lesions are observed on the confirmatory bone scan, study therapy is continued. If additional new lesions are observed, then the patient has experienced progression. Progression in this situation is dated as the time of the first reassessment scan.

b. For subsequent scheduled reassessments: If no new lesions are observed, study therapy will continue. If new lesions are observed, this is evidence of disease progression. Date of progression is the date at which the scan was obtained.

7.7 Post-treatment PSA Changes

All patients will be evaluated for PSA decline. Patients with disease that is not measurable will be eligible for this study and will be assessed for response based on changes in PSA and serial bone scans (if appropriate). The baseline serum PSA must be at least 2 ng/mL. Patients who show PSA increases will not be evaluated for PSA progression prior to 12 weeks of study therapy.

a. 30% and 50% PSA Decline: PSA decline of at least 30% and 50%, respectively, from baseline confirmed by a second measurement at least 3 weeks later. The reference for these declines should be a PSA measured within 2 weeks prior to starting therapy.

b. PSA Progression: Prostate Cancer Working Group 2 (PCWG2) Criteria will be reported. PSA progression occurs when the PSA has increased 25% or greater above nadir and an absolute increase of 2 ng/mL or more from the nadir is documented. Where no decline is observed, PSA progression similarly occurs when a 25% increase from baseline value along with an increase in absolute value of 2 ng/mL or more. Patients will receive a minimum of 12 weeks of therapy prior to being evaluable for this endpoint. PSA progression (without evidence of progression on scans) will not be criteria for discontinuation of study therapy.

c. PSA Response Duration: The PSA response duration commences on the date of the first 50% decline in PSA. The response duration ends when the PSA value increases by 25% above the nadir, provided that the increase in the absolute-value PSA level is at least 5 ng/mL or back to baseline, whichever is lower.

d. Progressive Disease by PSA (as defined by PSA Progression, above)

e. Time to PSA Progression: The start of the time to PSA progression is the day treatment is initiated. The end date is the date of the first PSA rise over the determined PSA PD value.

7.8 Progressive disease (PD)

Progressive disease will be defined by any one of the following:

1. Appearance of new metastatic lesions outside the bone
2. New metastatic lesions on bone scan confirmed as described above
3. Development of an indication for radiotherapy while on treatment
4. Unequivocal progression of non-target lesions
5. Global deterioration of health status requiring discontinuation of treatment without objective evidence of disease progression

Note that PSA progression (as defined above) alone does not meet the criteria for progressive disease.

8 Statistical Considerations

8.1 Sample Size Justification

The sample size determination is based on the primary endpoints of the study, to determine the maximally tolerated and recommended phase 2 dose and schedule of the combination. 6 patients will be enrolled at each of the three planned cohorts in Part A of the study. Therefore, up to 18 evaluable patients total will be enrolled in Part A.

In Part B, the primary efficacy endpoint is objective response rate by RECIST 1.1 criteria. A sample size of N = 25 patients is chosen to provide a preliminary estimate of the response rate with the recommended phase 2 dose and schedule of ^{177}Lu-PSMA-617 in combination with pembrolizumab. Using a historical control objective response rate with pembrolizumab monotherapy in mCRPC of 10% (e.g. KEYNOTE-028 study, in press Annals of Oncology), and an alternative hypothesis of ORR = 40%, a sample size of 25 patients provides 93% power to detect a difference of this magnitude in ORR with a bi-directional level of significance of 0.05.

8.2 Accrual

The estimated accrual period is 21 months, and total study duration (first patient enrolled to last patient off study) is 36 months.

8.3 Interim Analyses

Patients in Part A will be evaluated for dose-limiting toxicities during the first two cycles of treatment as outlined above in Section 7.1.

In Part B, an interim safety analysis will be performed after 6 patients are enrolled. If the DLT frequency exceeds 33%, study accrual will be halted and alternative dose and schedule of Lu-PSMA-617 may be evaluated based on the prior results of Part A of the study.

8.4 Analytic Plan

8.4.1 Analytic Plan for Primary Endpoints

Part A:

The recommended phase 2 dose and schedule of ^{177}Lu-PSMA-617 will be determined from the aggregate of the safety and efficacy data observed.

Part B:

Objective response is defined as the best response (complete response or partial response) defined by RECIST v1.1, from the start of treatment until disease progression or recurrence, where the reference for progressive disease is the smallest measurements recorded from start of treatment. To be assigned a status of objective response (CR or PR), changes in tumor measurements must be confirmed by repeat scans no less than 4 weeks after the criteria for

response are first met. The proportion of patients with objective response will be descriptively reported with 95% confidence interval.

8.4.2 Analytic Plan for Secondary Endpoints

Median duration of response

Median duration of response will be estimated using Kaplan-Meier product limit method, starting with the date of first scan indicating response until loss of response or progression, or death, whichever occurs first.

PSA response rate

The proportion of patients who achieve a greater than 50% decline from baseline PSA drawn on C1D1, at any point in the treatment course, will be descriptively reported along with 95% confidence interval.

PSA progression free survival

PSA progression will be defined using PCWG3 criteria. Median PSA progression free survival will be estimated using the Kaplan-Meier method. Duration will be measured from date of first treatment to date of first PSA meeting criteria for PSA progression.

Time to symptomatic skeletal related event

Symptomatic skeletal related event is defined as the first occurrence of one or more of the following: symptomatic fracture, surgery or radiation to bone, or spinal cord compression. The median time to symptomatic skeletal related event will be determined using Kaplan-Meier method.

Safety
The incidence and severity of adverse events related to study treatment will be descriptively reported using CTCAE v4.0.

Radiographic progression free survival and 6-month rPFS rate:
Radiographic progression of disease will be defined by RECIST v1.1 and PCWG3 criteria. For the first scheduled reassessment by bone scan (at Cycle 2), new bone lesions will require a confirmatory bone scan 6 or more weeks later. Median PFS will be estimated using the Kaplan-Meier method. Durations will be measured from the first day of study treatment (Cycle 1) to the first date of radiographic progression or death, whichever occurs first. Patients who discontinue study treatment for toxicity, withdraw from the study, or have PSA-only progression will be censored at the date of the last radiographic tumor assessment. Patients who discontinue treatment for clinical progression or deterioration will be included in the analysis.

Overall survival

Median overall survival and 95% confidence interval will be estimated using the Kaplan-Meier method. Duration will be measured from first date of study therapy to date of death from any cause.

8.4.3 Analytic Plan for Correlative Endpoints

Lesion-specific response rate by baseline PSMA avidity on ^{68}Ga-PSMA-11 PET:

All target lesions on baseline cross-sectional imaging will be assessed for response (e.g. > 30% reduction in longest diameter) on subsequent imaging during the course of study. The median baseline uptake on Ga-PSMA-11 PET between responding versus non-responding lesions will be compared using Mann-Whitney test. In addition, Spearman's rank correlation will be used to compare baseline lesion uptake on Ga-PSMA-11 PET with percent change from baseline in longest tumor diameter on subsequent cross-sectional imaging on study.

Percent change from baseline in T cell repertoire, circulating T cell subsets, tumor infiltrating lymphocytes, and tumor PD-L1 expression by immunohistochemistry after one priming dose of Lu-PSMA RLT:

T cell repertoire will be estimated using Morasita's distance as previously described. Quantification of circulating T cell subsets and TILs will be performed using flow cytometry and immunohistochemical analysis of tumor tissue and whole blood, respectively. PD-L1 expression will be analyzed as the percentage of tumor cells with positive surface expression. Correlative testing may be performed at outside laboratory facilities (i.e. Strata Oncology). The percent change from baseline in these parameters will be reported using descriptive statistics (e.g. median, range, standard deviation). In an exploratory fashion, the study cohort will be dichotomized by those with percent change from baseline above and below median and efficacy outcomes will be compared between dichotomized subgroups using log-rank test for time-to-event endpoints (e.g. rPFS, duration of response) and Fisher's exact test for categorical variables (e.g. objective response rate, 6-month rPFS rate, PSA50 response rate).

Genomic Sequencing

We will utilize only CLIA-certified laboratories (e.g. Strata Oncology) to identify genetic mutations within mCRPC tumors to provide genetic information that can be returned to patient to potentially inform treatment decisions. Certain genomic sequencing will be performed by Strata Oncology. Strata Oncology is a precision oncology company that utilizes the StrataNGS assay, which is a 90-gene targeted assay that detects actionable genetic alterations (standard of care markers and clinical trial eligibility markers). The assay sequences DNA and RNA from FFPE slides or blocks for specific single nucleotide variants, multi-nucleotide variants, small insertions and deletions, gene fusions, exon skipping mutations, copy number changes, and de novo deleterious mutations in tumor suppressor genes. The StrataNGS assay will also measure expression levels for some immuno-oncology biomarkers that are not yet reported clinically, this data will be available to Strata for correlative analyses and future test expansion.

Tests are ordered online by physicians through a secure Physician Portal, and the patient's leftover tumor material is shipped to the laboratory (e.g. Strata Oncology) for testing. Positive and negative genomic alterations are reported and associated FDA-approved therapies and clinical trials are noted. The UCSF treating physician will discuss the results with the patient (including matched drug trials and approved therapies). Participant information (name, DOB, MRN, subject ID) will be provided to the testing laboratory to ensure that patients continue to have access to their genetic sequencing data should they decide to seek care elsewhere besides UCSF. Laboratories performing testing will not contact patients at any point during or

after study participation. Sample blocks sent to the testing laboratories will be returned to UCSF and will not be stored at these laboratories for future testing. Consenting to participate in this study does not enroll the participant in Strata's separate observational study – The Strata Trial (NCT03061305). Participants would need to sign a separate consent form for Strata Oncology to use the data for any other purpose other than what is specified in this protocol.

Association Between Tumor Dosimetry with Response

Patients will undergo optional SPECT/CT dosimetry scan on C1D2, approximately 24 hours after completion of priming dose of 177Lu-PSMA-617. The median tumor dosimetry on SPECT/CT between responding versus non-responding lesions will be compared using Mann-Whitney test. In addition, Spearman's rank correlation will be used to compare tumor dosimetry on SPECT/CT with percent change from baseline in longest tumor diameter on subsequent cross-sectional imaging on study.

9 Study Management

9.1 Pre-study Documentation

This study will be conducted in accordance with the ethical principles that have their origin in the Declaration of Helsinki as stated in 21 CFR §312.120(c)(4); consistent with GCP and all applicable regulatory requirements.

Before initiating this trial, the Investigator will have written and dated approval from the Institutional Review Board for the protocol, written informed consent form, subject recruitment materials, and any other written information to be provided to subjects before any protocol related procedures are performed on any subjects.

The clinical investigation will not begin until either FDA has determined that the study under the Investigational Drug Application (IND) is allowed to proceed or the Investigator has received a letter from FDA stating that the study is exempt from IND requirements.

The Investigator must comply with the applicable regulations in Title 21 of the Code of Federal Regulations (21 CFR §50, §54, and §312), GCP/ICH guidelines, and all applicable regulatory requirements. The IRB must comply with the regulations in 21 CFR §56 and applicable regulatory requirements.

9.2 Institutional Review Board Approval

The protocol, the proposed informed consent form, and all forms of participant information related to the study (e.g. advertisements used to recruit participants) will be reviewed and approved by the UCSF Institutional Review Board. Prior to obtaining IRB approval, the protocol must be approved by the Helen Diller Family Comprehensive Cancer Center Site Committee and by the Protocol Review Committee (PRC). The initial protocol and all protocol amendments must be approved by the IRB prior to implementation.

9.3 Informed Consent

All participants must be provided a consent form describing the study with sufficient information for each participant to make an informed decision regarding their participation. Participants must sign the IRB-approved informed consent form prior to participation in any study specific

procedure. The participant must receive a copy of the signed and dated consent document. The original signed copy of the consent document must be retained in the medical record or research file.

9.4 Changes in the Protocol

Once the protocol has been approved by the IRB, any changes to the protocol must be documented in the form of an amendment. The amendment must be signed by the Investigator and approved by PRC and the IRB prior to implementation.

If it becomes necessary to alter the protocol to eliminate an immediate hazard to patients, an amendment may be implemented prior to IRB approval. In this circumstance, however, the Investigator must then notify the IRB in writing within five (5) working days after implementation. The Study Chair and the UCSF study team will be responsible for updating any participating sites.

10 Protection of Human Subjects

10.1 Protection from Unnecessary Harm

Each clinical site is responsible for protecting all subjects involved in human experimentation. This is accomplished through the IRB mechanism and the process of informed consent. The IRB reviews all proposed studies involving human experimentation and ensures that the subject's rights and welfare are protected and that the potential benefits and/or the importance of the knowledge to be gained outweigh the risks to the individual. The IRB also reviews the informed consent document associated with each study in order to ensure that the consent document accurately and clearly communicates the nature of the research to be done and its associated risks and benefits.

10.2 Protection of Privacy

Patients will be informed of the extent to which their confidential health information generated from this study may be used for research purposes. Following this discussion, they will be asked to sign the HIPAA form and informed consent documents. The original signed document will become part of the patient's medical records, and each patient will receive a copy of the signed document. The use and disclosure of protected health information will be limited to the individuals described in the informed consent document.

References

1. Afshar-Oromieh A, Malcher A, Eder M, et al. PET imaging with a [68Ga]gallium-labelled PSMA ligand for the diagnosis of prostate cancer: Biodistribution in humans and first evaluation of tumor lesions. Eur J Nucl Med Mol Imaging 2013;40:486-95.

2. Afshar-Oromieh A, Avtzi E, Giesel FL, et al. The diagnostic value of PET/CT imaging with the 68Ga-labeled PSMA ligand HBED-CC in the diagnosis of recurrent prostate cancer. Eur J Nucl Med Mol Imaging 2015;42(2):197-209.

3. Afshar-Oromieh A, Zechmann CM, Malcher A, et al. Comparison of PET imaging with a (68)Ga-labelled PSMA ligand and (18)F-choline-based PET/CT for the diagnosis of recurrent prostate cancer. Eur J Nucl Med Mol Imaging 2014;41:11–20.

4. Agarwal KK, Singla S, Arora G, et al. 177Lu-EDTMP for palliation of pain from bone metastases in patients with prostate and breast cancer: a phase II study. Eur J Nucl Med Mol Imaging 2015;42:79-88.

5. Ahkhtar NH, Orrin P, Saran A, et al. Prostate-specific membrane antigen-based therapeutics. Adv Urol 2012;973820.

6. Bahl A, Oudard S, Tombal B, et al. Impact of cabazitaxel on 2-year survival and palliation of tumour-related pain in men with metastatic castration-resistant prostate cancer treated in the TROPIC trial. Ann Oncol 2013;24(9):2402–2408.

7. Banerjee S, Pillai MRA, Knapp FF. Lutetium-177 therapeutic radiopharmaceuticals: linking chemistry, radiochemistry and practical applications. Chem Rev 2015;115:2934-74.

8. Beer TM, Armstrong AJ, Rathkopf DE, et al. Enzalutamide in metastatic prostate cancer before chemotherapy. N Engl J Med 2014;371:424–433.

9. Benesova M, Bauder-Wust U, Schafer M, et al. Linker modification strategies to control the prostate-specific membrane antigen (PSMA)-targeting and pharmacokinetic properties of DOTA-conjugated PSMA inhibitors. J Med Chem 216;59:1761-1775.

10. Benesova M, Schafer M, Bauder-Wust U, et al. Preclinical evaluation of a tailor-made DOTA-conjugated PSMA inhibitor with optimized linker moiety for imaging and endoradiotherapy of prostate cancer. J Nucl Med 2015;56:914-920.

11. Berthold DR, Pond GR, Soban F, et al. Docetaxel plus prednisone or mitoxantrone plus prednisone for advanced prostate cancer: updated survival in the TAX 327 study. J Clin Oncol 2008;26:242–245.

12. Castellucci P, Ceci F, Graziani T, et al. Early biochemical relapse after radical prostatectomy: which prostate cancer patients may benefit from a restaging 11C-Choline PET/CT scan before salvage radiation therapy? J Nucl Med. 2014;55:1424–1429.

13. Danthala M, Kallur KG, Prashant GR, et al. 177Lu-DOTATATE therapy in patients with

neuroendocrine tumours: 5 years' experience from a tertiary cancer centre in India. Eur J Nucl Med Mol Imaging 2014;41:1319-26.

14. Das T, Pillai MRA. Options to meet the future global demand of radionuclides for radionuclide therapy. Nucl Med Bio 2013;40:23-32.

15. de Bono JS, Logothetis CJ, Molina A, et al. Abiraterone and increased survival in metastatic prostate cancer. N Engl J Med 2011;364:1995–2005.

16. Delker A, Fendler WP, Kratochwil C, et al: Dosimetry for 177Lu-DKFZ-PSMA-617: A new radiopharmaceutical for the treatment of metastatic prostate cancer. Eur J Ncl Med Mol Imaging 2016;43:42-51.

17. Eiber M, Maurer T, Souvatzoglou M, et al. Evaluation of hybrid Ga-PSMA ligand PET/CT in 248 patients with biochemical recurrence after radical prostatectomy. J Nucl Med 2015;56:668-674.

18. Fizazi K, Scher HL, Molina A, et al. Abiraterone acetate for treatment of metastatic castration-resistant prostate cancer: final OS analysis of the COU-AA-301 randomised, double-blind, placebo controlled phase 3 study. Lancet Oncol 2012;13:983–992.

19. Goumi E, Canovas C, Goncalves V, et al. (R)-NODAGA-PSMA: A versatile precursor for radiometal labeling and nuclear imaging of PSMA-positive tumors. PLoS One 2015;10:e0145755.

    Heck MM, Retz M, D'Alessandria C, et al. Systemic radioligand therapy with (177)Lu labeled prostate specific membrane antigen ligand for imaging and therapy in patients with metastatic castration resistant prostate cancer. J Urol 2016;196:382-91.

20. Hohberg M, Eschner W, Schmidt M, et al. Represent organs at risk for radionuclide therapy of prostate cancer with [177Lu]DKFZ-PSMA-617. Mol Imaging Bio 2016;18:437-45.

21. Israeli RS, Powell CT, Corr JG, et al. Expression of the prostate-specific membrane antigen. Cancer Res 1994;54:1807-1811.

22. Kabasakal L, AbuQbeitah M, Aygun A, et al. Pre-therapeutic dosimetry of normal organs and tissues of 177Lu-PSMA-617 prostate-specific membrane antigen (PSMA) inhibitor in patients with castration-resistant prostate cancer. Eur J Nucl Med Mol Imaging 2015;42:1976-1983.

23. Kantoff PW, Higano CS, Shore ND, et al. Sipuleucel-T immunotherapy for castrate-resistant prostate cancer. N Eng J Med 2010;363:411–422.

24. Kratchwil C, Giesel FL, Eder M, et al. [177Lu]Lutetium-labelled PSMA ligand -induced remission in a patient with metastatic prostate cancer. Eur J Nucl Med Mol Imaging 2015;42:987-988.

25. Kratochwil C, Giesel FL, Stefanova M, et al. PSMA-targeted radionuclide therapy of metastatic castration-resistant prostate cacner with Lu-177 labeled PSMA-617. J Nucl Med 2016;57:1170-1176.

26. Kwekkeboom DJ, Bakker WH, Kooij PP, et al. [177Lu-DOTAOTyr3]octreotate: comparison with [111In-DTPAo]octreotide in patients. Eur J Nucl Med 2001;28:1319-25.

27. Parker C, Nilsson S, Heinrich D, et al. Alpha emitter radium-223 and survival in metastatic prostate cancer. N Engl J Med 2013;369:213-223.

28. Petrylak D, Tangen CM, Hussain MH, et al. Docetaxel and estramustine compared with mitoxantrone and prednisone in hormone refractory prostate cancer. N Engl J Med 2004;351:1513–1520.

29. Pillai MRA, Chakraborty S, Das T, et al. Production logistics of ^{177}Lu for radionuclide therapy. Appl Radiat Isot 2003;59:109-18.

30. Rajasekaran SA, Anilkumar G, Oshima E, et al. A novel cytoplasmic tail MXXXL motif mediates the internalization of prostate-specific membrane antigen. Mol Biol Cell 2003;14:4835-4845.

31. Ryan CJ, Smith MR, de Bono JS, et al. Abiraterone in metastatic prostate cancer without previous chemotherapy. N Engl J Med 2013;368:138–148.

32. Scher HL, Fizazi K, Saad F, et al. Increased survival with enzalutamide in prostate cancer after chemotherapy. N Engl J Med 2012;367:1187–1197.

33. Shakespeare TP. Effects of prostate-specific membrane antigen positron emission tomography on the decision-making of radiation oncologists. Radiat Oncol 2015;10:233.

34. Siegel RL, Miller KD, Jemal A. Cancer Statistics, 2015. CA Cancer J Clin 2015;65:5–29.

35. Sterzing F, Kratochwil C, Fiedler H, et al. 68Ga-PSMA-11 PET/CT: A new technique with high potential for the radiotherapeutic management of prostate cancer patients. Eur J Nucl Med Mol Imaging 2016;43:34-41.

Tagawa ST, Milowsky MI, Morris M, et al. Phase II study of lutetium-177-labeled anti-prostatic-specific membrane antigen monoclonal antibody J591 for metastatic castration-resistant prostate cancer. Clin Cancer Res 2013;19:5182-5191.

36. Tannock IF, dewit R, Berry WR, et al. Docetaxel plus prednisone or mitoxantrone plus prednisone for advanced prostate cancer. N Engl J Med 2004;351:1502–1512.

37. Thapa P, Nikam D, Das T, et al. Clinical Evaluation of efficacy and safety of 177Lu-EDTMP in patients with painful skeletal metastasis: a multiparametric comparison with 153Sm-EDTMP on equidose basis. J Nucl Med 2015;56:1513-1519.

38. van Leeuwen PJ, Stricker P, Hruby G, et al. 68Ga-Prosate-specific membrane antigen has a high detection rate of prostate cancer recurrence outside the prostatic fossa in patients being considered for salvage radiation treatment. BJU Int 2016;117:732-739.

39. Wright GL, Jr, Haley C, Beckett ML, et al. Expression of prostate-specific membrane antigen in normal, benign, and malignant prostate tissues. Urol Oncol 1995;1:18-28.

40. Wright GL, Jr, Grob BM, Haley C, et al. Upregulation of prostate-specific membrane antigen after androgen-deprivation therapy. Urology 1996;48:326-334.

41. Zaknun JJ, Bodei L, Mueller-Brand J, et al. The joint IAEA, EANM, and SNMMI practical guidance on peptide receptor radionuclide therapy (PRRNT) in neuroendocrine tumors. Eur J Nucl Med Mol Imaging 2013;40:800-816.

Appendix 1 Performance Status Criteria

ECOG Performance Status Scale		Karnofsky Performance Scale	
Grade	Descriptions	Percent	Description
0	Normal activity<br>Fully active, able to carry on all pre-disease performance without restriction	100	Normal, no complaints, no evidence of disease
		90	Able to carry on normal activity; minor signs or symptoms of disease
1	Symptoms, but ambulatory<br>Restricted in physically strenuous activity, but ambulatory and able to carry out work of a light or sedentary nature (*e.g.*, light housework, office work)	80	Normal activity with effort; some signs or symptoms of disease
		70	Cares for self, unable to carry on normal activity or to do active work
2	In bed < 50% of the time<br>Ambulatory and capable of all self-care, but unable to carry out any work activities<br>Up and about more than 50% of waking hours	60	Requires occasional assistance, but is able to care for most of his/her needs
		50	Requires considerable assistance and frequent medical care
3	In bed > 50% of the time<br>Capable of only limited self-care, confined to bed or chair more than 50% of waking hours	40	Disabled, requires special care and assistance
		30	Severely disabled, hospitalization indicated<br>Death not imminent
4	100% bedridden<br>Completely disabled<br>Cannot carry on any self-care<br>Totally confined to bed or chair	20	Very sick, hospitalization indicated<br>Death not imminent
		10	Moribund, fatal processes progressing rapidly
5	Dead	0	Dead

Appendix 2 Data and Safety Monitoring Plan for a Phase 1 Study

The UCSF Helen Diller Family Comprehensive Cancer Center (HDFCCC) Data and Safety Monitoring Committee (DSMC) is responsible for monitoring data quality and subject safety for all HDFCCC institutional clinical studies. A summary of DSMC activities for this study include:

- Review of subject data
- Review of suspected adverse reactions considered "serious"
- Monitoring every six months (depending on study accrual)
- Minimum of a yearly regulatory audit

Monitoring and Reporting Guidelines

Investigators will conduct continuous review of data and subject safety and discuss each subject's treatment at monthly Site Committee meetings. These discussions are documented in the Site Committee meeting minutes. The discussion will include the number of subjects, significant toxicities in accordance with the protocol, and observed responses.

All institutional Phase 2 or 3 studies are designated with a moderate risk assessment. The data is monitored twice per year with twenty percent of the subjects monitored (or at least three subjects if the calculated value is less than three).

<u>Adverse Event Review and Monitoring</u>

All grade(s) 3-5 adverse events, whether or not unexpected, and whether or not considered to be associated with the use of the study drug, will be entered into OnCore®, UCSF's Clinical Trial Management System.

All grade(s) 3-5 adverse events entered into OnCore® will be reviewed on a monthly basis at the Site Committee meetings. The Site Committee will review and discuss the selected toxicity, the toxicity grade, and the attribution of relationship of the adverse event to the administration of the study drug(s).

In addition, all suspected adverse reactions considered "serious" entered into OnCore®, will be reviewed and monitored by the Data and Safety Monitoring Committee on an ongoing basis and discussed at DSMC meetings, which take place every six weeks.

If a death occurs during the treatment phase of the study or within 30 days after the last administration of the study drug(s) and it is determined to be related either to the study drug(s) or to a study procedure, the Investigator or his/her designee must notify the DSMC Chair within **1 business day** of knowledge of this event. The contact may be by phone or e-mail.

<u>Increase in Adverse Event Rates</u>

If an increase in the frequency of Grade 3 or 4 adverse events (above the rate reported in the Investigator Brochure or package insert) is noted in the study, a report should be submitted to the DSMC at the time the increased rate is identified. The report will indicate if the incidence of adverse events observed in the study is above the range stated in the Investigator Brochure or package insert.

If at any time the Investigator stops enrollment or stops the study due to safety issues, the DSMC Chair and DSMC Manager must be notified within 1 business day via e-mail. The DSMC must receive a formal letter within 10 business days and the IRB must be notified.

The decision to proceed to Part B of the study must be approved by DSMC.

Data and Safety Monitoring Committee Contacts:

DSMC Chair: Robin Kate Kelley, MD
Phone: 415-353-9888
Email: Katie.Kelley@ucsf.edu
Address: UCSF
San Francisco, CA 94158

DSMC Monitors
Box 0128
UCSF Helen Diller Family
Comprehensive Cancer Center
San Francisco, CA 94143

Appendix 3 UCSF Policy/Procedure for Required Regulatory Documents for a UCSF Investigator-Initiated Oncology Clinical Trials with an Investigator held Investigational New Drug (IND)

Purpose

This policy defines the required Regulatory Documents for Single Site and Multicenter Investigator Initiated Oncology Clinical Trials at the Helen Diller Family Comprehensive Cancer Center (HDFCCC) where the Principal Investigator (PI) holds the IND.

Background

The International Conference on Harmonization (ICH) Good Clinical Practices (GCP) Guidelines define Essential Regulatory Documents as those documents which individually and collectively permit evaluation of the conduct of a trial and the quality of data produced. These documents serve to demonstrate compliance with standards of GCP and with all applicable regulatory requirements. Filing essential documents in a timely manner can greatly assist in the successful management of a clinical trial.

The Regulatory Documents will consist of electronic files in both iMedRIS and OnCore®, as well as paper files in the Regulatory Binders for both the Coordinating Site and the Participating Site(s) in the HDFCCC Investigator Initiated Oncology Clinical Trials.

Procedures

1. HDFCCC Essential Regulatory Documents

Documents Filed in iMedRIS:

- IRB approvals for initial submission of application, all modifications, and continuing annual renewals
- Current and prior approved protocol versions with signed protocol signature page(s)
- IRB approval letters and Informed Consent Form(s) (ICF)
- Current and prior versions of the Investigator Brochure (IB).
- Serious Adverse Event Reporting
- Protocol Violations and Single Patient Exception (SPE) Reports to IRB with supporting fax documentation

Documents Filed in OnCore®:

- Package Insert (if the study drug is commercial) or Investigator Brochure
- Protocol Review Committee (PRC) approved protocols, protocol amendments and Summary of Changes (SOC)
- Patient handouts
- Screening/enrollment log
- Data and Safety Monitoring Committee (DSMC) monitoring reports
- OnCore® Case Report Form (CRF) completion manual

Documents Filed in Regulatory Binder:

- Completed Food and Drug Administration (FDA) 1572 document with Principal Investigator's signature
- For all Principal Investigators and Sub-Investigators listed on the FDA 1572, will need Financial Disclosure Forms, CVs, MD Licenses, Drug Enforcement Agency (DEA) Licenses, and Staff Training Documents (i.e. Collaborative Institute Training Initiative (CITI), etc.)
- Site Initiation Visit (SIV) minutes and correspondence with participating site(s).
- As applicable, approvals for Biosafety Committee, Radiation Committee, and Infusion Center
- Serious Adverse Event (SAE) reports to IRB and sponsor.
- MedWatch reporting to FDA and sponsor
- Delegation of Authority Form
- Drug Destruction Standard Operating Procedure (SOP)
- For all laboratories listed on the FDA 1572, will need CLIA certifications, CAP certifications, lab licenses, CVs of Lab Directors, and laboratory reference ranges

Appendix 4 Radioprotection Precautions for Patients Treated with ^{177}Lu-PSMA

TECHNICAL DATA

The principle characteristics of lutetium-177 (^{177}Lu) are specified in Table 1 (from *Les principales caractéristiques du 177 lutetium (^{177}Lu)* and *Guide Pratique Radionucléides & Radioprotection*, D. Delacroix, J.P. Guerre, P. Leblanc, EDP Sciences, 2004).

$^{177}_{71}$Lu Période : 6,71 jours Activité massique : 4,07.10^{15} Bq.g^{-1} Groupe de risque : 4

Lutétium - 177

Principales émissions

	Gamma / X		Beta (Emax)		Electrons		Alpha	
	E (keV)	%	E (keV)	%	E (keV)	%	E (keV)	%
E1	55	5	208	11	6	9		
E2	113	6	384	9	48	5		
E3	208	11	497	79	110	9		
% omis		5		< 1		4		

Seuils d'exemption

Quantité en Bq	1.10^{7}
Concentration en Bq.g^{-1}	1.10^{3}

Transport (Bq)

A1	3.10^{13}
A2	7.10^{11}

Table 1. Characteristics of ^{177}Lu.

^{177}Lu is generally produced using enriched ^{176}Lu placed in a reactor, following a reaction of the type [^{176}Lu n,γ ^{177}Lu]. This reaction results in the production of a small amount of ^{177}Lu meta-stable (^{177m}Lu) which varies according to sources between 10^{-5} and 4×10^{-4} per MBq of ^{177}Lu produced. The characteristics of this radionuclide are given in Table 2.

Période : 160,4 jours Activité massique : 1,69.10^{14} Bq.g^{-1} Groupe de risque : 3* $^{177m}_{71}$Lu/$^{177}_{71}$Lu
Descendant : Embr. = 0,215 ; T = 6,71 jours

Lutétium - 177m / Lutétium - 177

Principales émissions

	Gamma / X		Beta (Emax)		Electrons		Alpha	
	E (keV)	%	E (keV)	%	E (keV)	%	E (keV)	%
E1	328	18	152	79	5	60		
E2	379	28	384	2	45	51		
E3	419	20	497	17	95	41		
% omis		450		12		60		

Seuils d'exemption

Quantité en Bq	-
Concentration en Bq.g^{-1}	-

Transport (Bq)

A1	-
A2	-

Table 2. Characteristics of ^{177m}Lu.

RECOMMENDATIONS

Based on radiation exposure calculations employing whole body clearance data (Wehrmann C et al, 2007) and exposure rates at one meter at 24 h (7.5 ± 3.6 µSv/h, Dpt. of Nucl. Med. Erasmus MC Rotterdam), patients may be treated on an outpatient basis for an administration of ^{177}Lu-PSMA. As a precaution, it is recommended that patients be kept in radiation isolation for a period of 4–5 hours following administration, and be allowed to urinate during that time, and before release. This precaution is deemed prudent because this is the primary method of elimination. At the time of release, the patient is given written instructions which summarize the precautions to take, in order to keep the exposure to others below regulatory limits.

Appendix 5 Recommended Precautions for Patients Treated with ^{177}Lu-PSMA

Below are recommendations for radiation safety when administered 177Lu-PSMA-617. The local radiation safety officer will meet with each patient and develop a plan to minimize the potential exposure. In accordance with the medical staff, you have agreed to receive a treatment using a radioactive medicine. The efficacy of this medicine in treating your tumor is due to the radioactivity; but this is also the reason why it is necessary to follow certain precautions in order to limit the exposure of the people around you.

Considering current knowledge and experience in the field and the physical and radiopharmaceutical properties of the radiopharmaceutical, it is estimated that the health risks to your family members and the general public are low. However, you must adhere to the following rules to maximize the safety of other persons. These rules are the result of many years of experience in the use of radioactivity in medicine, and they include recommendations issued by international organizations.

1. **General rule**

You must avoid close contact with people who live with you, and should try to keep a distance of at least one meter for 2 to 3 days after you receive each treatment.

2. **Use of toilets**

Toilets must be used in a seated position, even for men. It is absolutely necessary to use toilet paper each time. It is also important to wash your hands to avoid contaminating the door handles. It is strongly recommended to move your bowels every day and use a laxative if necessary. Furthermore, drink frequently and try to urinate every hour on the day you received treatment and on the two following days. Follow your doctor's advice on how much fluid to drink.

3. **Contact with children less than 10 years old**

It is strongly recommended to limit contact with children who are less than 10 years old for 7 days after the administration.

4. **The spouse and people in the family circle**

It is strongly advised to sleep in separate beds at a distance of at least 1 meter. Sexual activity is not advised for 7 days after each treatment.

Potential consequences do not justify additional particular recommendation concerning elderly persons.

Contact with pregnant women should follow the same restrictions as recommended for children less than 10 years old.

5. **Contraception and pregnancy**

Due to the radionuclide part of this radiopharmaceutical, men of child-bearing potential must refrain from procreation by using effective contraceptive methods during the treatment and for 2 months after.

6. **People who need extra assistance**

People who are confined to the bed or have reduced mobility will preferably receive assistance by a care provider. It is recommended that when providing assistance in the bathroom, the care provider wear disposable gloves for 2-3 days after each administration. In the case of the use of special medical equipment such as catheters, colostomy bags bedpan, water nozzle, or anything that could be contaminated by your body fluids, they must be emptied immediately in the toilet and then cleaned. If anyone helps you clean up vomit, blood, urine, or stool they should wear plastic gloves; the gloves should then be put in the specified trash plastic bag (according to the recommendation stated in Section 8 below).

7. **Dishes and bathroom accessories**

For the first two days after your treatment wipes and/or toilet paper must be flushed down the toilet. Always wash your hands well after using the toilet.

It is strongly recommended to shower every day for at least the first 7 days after your treatment.

Try to flush any tissues or any other items that contain anything from your body, such as blood, urine and feces down the toilet (at least for two days after administration). Items that cannot be flushed, such as menstrual pads and bandages, must be placed in specified plastic trash bags (according to the recommendation stated in Section 8).

Wash your underwear, pajamas, sheets and any clothes that contain sweat, blood or urine separately from the laundry of others in your household. Wash your items two or three times; use a standard washing machine; you do not need to use bleach and do not need extra rinses.

8. **Trash recommendations**

Keep the specified plastic trash bags separate from other trash. Keep the bags away from children and animals.

A member of the medical staff will tell you how and when to get rid of the specified plastic trash bag. You may be asked to bring the bag back to your treatment facility, or, after 70 days, the bag may be removed as other household waste.

9. **Professional activities**

Impact of the treatment on the ability to conduct professional activities has not been studied. Your general condition and the possible adverse reactions to treatment will be taken into account by your doctor to assess this ability.

10. **Hospitalization**

In the case that an unplanned hospitalization occurs, it is important to notify your doctor.

11. **Emergency care**

Carry your discharge letter with you at all times for at least 3 months after treatment with ^{177}Lu-PSMA.

If for any reason you require emergency medical assistance during the first week after your treatment, you should inform the medical providers about the nature, the date and the dose of your radioactive treatment.

12. **Travel**

Keep the discharge letter with you whenever you are travelling for at least 3 months after treatment.

13. **Summary of the most important recommended precautions after each ^{177}Lu-PSMA administration**

	Restriction to be followed after each administration of ^{177}Lu-PSMA	Number of days
**Day-time restrictions**	You may return to work after	2 to 3 days
	Avoid close contract (less than 1 meter) with children (less than 10 years old) and pregnant woman for	7 days
	Avoid extended time in public places for	2 to 3 days
**Night-time restrictions**	Sleep in a separate bed (separated by at least 1 meter) for	2 to 3 days
	Sleep in a separate bed from infants or children for	7 days
	Sleep in a separate bedroom from pregnant partners for	15 days

Appendix 6 National Cancer Institute Common Terminology Criteria for Adverse Events

Refer to the following website for complete CTCAE guide, version 4.0.3.

https://evs.nci.nih.gov/ftp1/CTCAE/CTCAE_4.03_2010-06-14_QuickReference_5x7.pdf
